# ERV3-MLT1 provides cis-regulatory elements for human placental functioning and are commonly dysregulated in human-specific preeclampsia

**DOI:** 10.1186/s13059-025-03821-1

**Published:** 2025-11-05

**Authors:** Rabia Anwar, Amit Pande, Manvendra Singh, Zhi Huang, Eve Hallett, Yiran Xie, Alexandra Gellhaus, Florian Herse, Olivia Nonn, Martin Gauster, Tamás Raskó, Matthias Selbach, Stefan Verlohren, Anne Cathrine Staff, Ralf Dechend, Ulrich Pecks, Sandra M. Blois, Laurence D. Hurst, Zsuzsanna Izsvák

**Affiliations:** 1 The Max Delbrück Center for Molecular Medicine in the Helmholtz Association (MDC), Robert-Rössle-Straße 10, 13125 Berlin, Germany; 2 University Health Network, Toronto, Canada; 3 Imagine Institute, Université Paris Cité, INSERM UMR 1163, 75015 Paris, France; 4 Institut Necker Enfants Malades (INEM), Université Paris Cité, INSERM UMR 1151, 75015 Paris, France; 5 The Milner Centre for Evolution, Department of Life Sciences, University of Bath, Bath, BA2 7AY UK; 6 Department of Obstetrics and Fetal Medicine, University Medical Center Hamburg-Eppendorf, Hamburg, Germany; 7 Department of Gynecology and Obstetrics, University Hospital Essen, Essen, Germany; 8 Experimental and Clinical Research Center, a joint cooperation between the Max Delbrück Center for Molecular Medicine and the Charité, Universitätsmedizin Berlin, 13125 Berlin, Germany; 9 Division of Cell Biology, Histology and Embryology, Gottfried Schatz Research Center, Medical University of Graz, Graz, Austria; 10 Medizinische Klinik Und Poliklinik II Lehrstuhl Für Zelluläre Immuntherapie Würzburg, Universitätsklinikum Würzburg, 97078 Würzburg, Germany; 11 Faculty of Medicine, University of Oslo, Oslo, Norway; 12 Division of Obstetrics and Gynaecology, Oslo University Hospital, Oslo, Norway; 13 Department of Cardiology and Nephrology, HELIOS Klinikum Berlin Buch, 13125 Berlin, Germany; 14 Maternal Health and Midwifery, Ludwig Maximillians University, Würzburg, Germany; 15 Department of Obstetrics, University Hospital of Würzburg, Germany Universitätsklinikum Würzburg, Würzburg, Germany

## Abstract

**Background:**

Owing to their transcription factor binding sites, endogenous retroviruses (ERVs) can act as cis-regulatory-elements (CREs). By invading genomes in waves, ERVs offer a substrate for lineage-specific adaptations but also, when dysregulated, for lineage-specific disorders. Their role as CREs in rapid placental evolution, and in the human-specific placental disorder preeclampsia, may thus provide a paradigmatic exemplar. Here then we systematically identify ERV-derived CREs controlling human placental gene expression with dysregulation in preeclampsia.

**Results:**

We identify 87 ERV-derived CREs located upstream of genes expressed in the placenta. A subset of nine, all belonging to the ERV3-MLT1/2 families and dating to the mouse–human common ancestor, are consistently dysregulated in trophoblasts from preeclampsia samples. Of the nine ERV3-MLT1-linked genes dysregulated in preeclampsia, five are novel candidates, while four were previously associated with preeclampsia, though their ERV-based regulation was not recognized. Focusing on EPS8L1, we predict enhancer activity of upstream MLT1(G1) and validate using reporter assay and genome editing. This vertebrate-specific gene is expressed in progenitor cytotrophoblasts and syncytiotrophoblasts and is overexpressed in preeclampsia, correlating with preeclampsia biomarkers and is not elevated in related pregnancy disorders. A soluble form of EPS8L1 is detectable in maternal plasma as early as between 24 weeks of gestation. EPS8L1 knockout in trophoblast in vitro is lethal, and its overexpression alters trophoblast behaviors characteristic of preeclampsia.

**Conclusions:**

We conclude that ERV3-MLT1functions as a trophoblast-specific CRE for several human genes and may be dysregulated in preeclampsia. As EPS8L1 has a form in maternal circulation, it may have utility in diagnostics.

**Supplementary Information:**

The online version contains supplementary material available at 10.1186/s13059-025-03821-1.

## Background

Most of the human genome consists of remnants of dead mobile elements, including transposable elements and endogenous retroviruses (ERVs). While this genomic “bloating” can be attributed in part to weaker purifying selection in populations with low effective size [1], mobile elements are nonetheless frequently co-opted into the functional genome. This recruitment occurs either because of their inherent capacity to function as cis-regulatory elements (CREs), or through the repurposing of components of their gene bodies for host biological functions [2].

One reason mobile elements often serve as CREs lies in the evolutionary pressure on them to hijack host transcription factors (TFs) to drive their own expression (and hence their propagation) [3]. This selects for mobile elements with TF binding capacity, biasing the genomic pool toward sequences with latent or active cis-regulatory potential. ERVs, which make up approximately 8% of the human genome and are the focus of this study, exemplify this. Their long terminal repeats (LTRs) frequently function as platforms for TF binding and can act as tissue-specific regulatory elements (CREs) (commonly promoters or enhancers) for host genes [4].

From a phylogenetic perspective, ERVs tend to invade in waves, extensively colonizing vertebrate genomes [5]. This property results in a dynamic and lineage-specific ERV landscape, making them a common source of lineage-specific transcripts (functional or spurious). Indeed, ERVs and other transposable elements contribute to 85% of primate-specific and over 20% of all human TF binding sites [6]. Their potential to be co-opted by the host genome makes them strong candidates for driving lineage-specific regulatory innovations. Conversely, their dysregulation may also underlie lineage-specific disorders.

In this context, the role of ERVs in placental biology and in the etiology of preeclampsia (PE) offers a compelling example. Not only is the placenta an unusually fast-evolving tissue [7] but PE is classically considered a human-specific disease [8], although there are sporadic reports of preeclampsia-like symptoms in chimpanzees [9] and gorillas [10], suggesting it may be restricted to great apes [11].

A substantial body of research supports the involvement of ERVs in placental evolution [12–19], with several reviews summarizing their functional co-option in this context [20–23]. The best-known example is Syncytin-1 (ERVWE1), derived from the HERV-W family, which is essential for trophoblast fusion during placental development [24]. Additional ERV-derived genes have been co-opted to suppress maternal immune responses and protect the fetus from viral infection [25–27]. ERVs also appear to underpin parallel placental evolution across species. For example, the primate-specific ERV1-MER21A element drives CYP19 expression in humans [4], while analogous ERV-derived promoters are found in bovine and ovine Cyp19 genes [28]. Recognition of the roles of ERVs in placental function and pathology has led to calls for prioritizing this research area [5].

Here, we aim to provide a systematic analysis of ERVs acting as CREs in human placental development and PE. Several features make ERV-derived sequences especially likely to play functional roles in this context. First, although silenced by epigenetic mechanisms in most somatic tissues, the placenta exhibits a more permissive epigenetic landscape [29], supporting widespread transcription of mobile element-derived sequences [30], possibly contributing to its rapid evolution [14]. Second, given that PE has an epigenetic component [31] and ERVs are highly responsive to epigenetic perturbations [32], ERVs represent plausible regulatory elements disrupted in the disorder. Furthermore, the regulation of imprinted genes and ERVs relies on similar epigenetic mechanisms [33], and since imprinting errors have been linked to PE [31], this raises the possibility that dysregulated ERVs may also contribute to the pathogenesis of PE.

Understanding the role of ERVs in PE is also clinically important. PE remains a leading cause of maternal and fetal morbidity [8], yet its etiology is poorly understood, and treatments are limited (primarily removal of the placenta). Classically defined as new-onset maternal hypertension with or without proteinuria after 20 weeks’ gestation, PE can now also be diagnosed based on other markers of organ dysfunction [34]. Despite etiological heterogeneity, PE consistently involves placental stress, impaired trophoblast invasion, and inadequate remodeling of spiral arteries [35]. In the canonical two-stage model [36], abnormal placentation leads to oxidative stress and dysregulated angiogenesis (reduced VEGF and PlGF, elevated sFLT1 and sENG) culminating in maternal endothelial dysfunction [37]. This stress is further exacerbated by inflammatory cytokine imbalances [38]. However, antioxidant treatments have failed to prevent PE [39], underscoring the need for a deeper understanding of its root causes. Prophylactic low-dose aspirin is currently recommended for high-risk pregnancies [34].

To better understand ERV activity as CREs in normal and pathological placentation, and to inform both etiological models and potential diagnostic tools, we set out to identify ERV-derived functional CREs with placenta-specific expression that are dysregulated in PE. Our premise is that if a given ERV-derived CRE is active in normal placentation, becomes dysregulated in PE, and is associated with a relevant phenotype, then it likely plays a biologically significant role in human placental development.

We developed a deep learning pipeline to systematically identify ERV-derived CREs with placental expression, yielding 87 candidates. Among these, we identified nine genes consistently dysregulated in PE, four of which have been previously associated with the disease [40], and five that are novel. All are regulated by members of the ERV3-MLT1 family. Notably, ERV-mediated regulation of the previously known genes had not been recognized. These genes tend to have expression specific to trophoblast.

To explore potential causal involvement of these candidates in PE, we focused on the least characterized gene, *EPS8L1* (*Epidermal Growth Factor Receptor Pathway Substrate 8 Like 1*), which has no known function. Using an in vitro model of PE with stable EPS8L1 overexpression (OE-EPS8L1) in trophoblasts, we performed high-throughput and functional assays. These revealed that oxidative stress and features of placental insufficiency arise from its dysregulation. EPS8L1 knockout proved lethal in these cells. As a secreted protein with PE-specific expression that correlates with known PE markers, EPS8L1 shows promise as a potential biomarker for diagnosing early-onset PE.

## Results

### Identification of 87 putatively ERV controlled trophoblast expressed genes

**Fig. 1:**
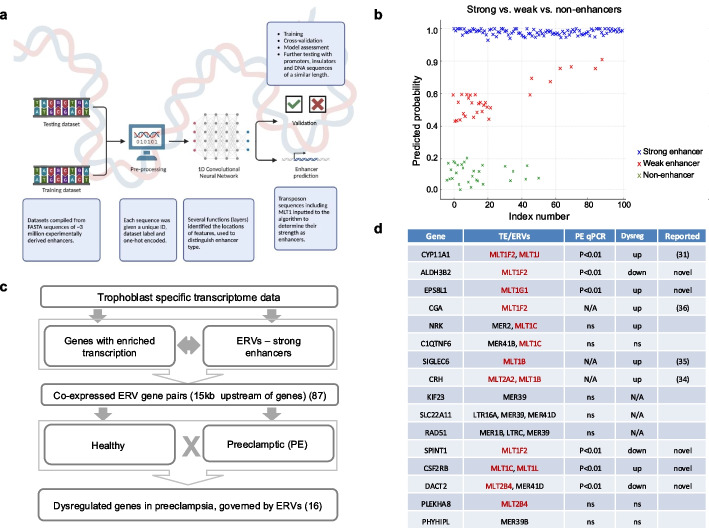
Identification of TE/ERV-driven dysregulated genes in pre-eclampsia. **a** Deep learning model workflow. The 1D CNN was trained on experimentally derived enhancers (see Methods and Additional file 7). The model calculates how ERV-derived transposons behave as enhancers, based on their DNA sequences alone. **b** Probability scores are used to classify ERV-derived sequences as “strong” enhancers, “weak” enhancers, or “non-enhancers.” (For the list of the classified ERVs, see Additional file 1: Table S1.) The *X*-axis displays an *index number*, a unique identifier assigned to each HERV sequence in our dataset. This index corresponds to the sequential order in which the sequences were input into the model, allowing each sequence to be individually traced back to its source or original genomic position. The *Y*-axis shows the probability score generated by the CNN, which determines the enhancer classification of each sequence. **c** Workflow to identify genes dysregulated in PE driven by trophoblast-specific ERV-derived enhancers. **d** Primary characterization of the shortlisted 16 genes using our strategy to identify genes significantly dysregulated in PE, driven by trophoblast specific TE-derived enhancers. Oslo cohort, control (*n* = 27), early-onset PE (*n* = 24), late-onset PE (*n* = 23). Note (i) that all TEs are ERVs; (ii) the enrichment (12/16) of MLT1/2-derived enhancers driving PE dysregulated genes (red); (iii) the five novel PE candidate genes. Column-PE qPCR summarizes qPCR validation (see also Fig. 2a). N/A, not applicable; ns, not significant; dysreg, dysregulated in PE

We began by systematically identifying functional ERV-derived enhancers in human trophoblast cells. To achieve this, we analyzed trophoblast ChIP-seq data, focusing on regulatory ERVs previously shown to be expressed in this cell type [17, 41]. For promoters and enhancers, we expected enrichments for H3K4me3 and H3K4me1/H3K27ac, respectively. We used a 1D Convolutional Neural Network (1D CNN) to calculate whether TE/ERVs behave as enhancers, based on their sequence attributes (Fig. 1a). The model calculates a probability score to classify sequences as “strong,” “weak,” or “non-enhancers” [42–44] (Fig. 1b and Additional files 1 and 2: Table S1, [43]), of which we identify 314, 11, and 20 respectively.

Following the hypothesis that ERV-derived regulatory sequences are co-opted as tissue specific enhancers [14], we sought to identify those ERV-derived sequences that potentially play a role regulating neighboring genes in trophoblasts determining whether there was a correlation between “strong” ERV enhancer with the expression of their neighboring genes. To find these “strong” ERV-gene pairs, the expression of the neighboring genes was compared with the expression of the ERVs located within a 15-kb region upstream of the transcription start site (TSS) of the genes (Fig. 1c). This identified 87 potential ERV enhancer-gene pairs (Additional file 2: Table S2).

### Identification of 9 ERV controlled genes dysregulated in PE

To identify PE candidate pairs, we filtered out from the 87 pairs those that were differentially expressed (in any direction) in the PE transcriptomes: Microarray data from Oslo cohort (25 placenta samples from PE pregnancies and 23 samples from healthy pregnancies) [31] and RNA-seq of primary human trophoblast cells (from placenta of 10 early-onset PE patients versus 8 healthy women) [45]. This identified 16 of the 87 ERV-associated gene pairs that were also dysregulated in our PE transcriptome (Fig. 1c). We noted a relative enrichment of MLT1/2 ERVs (12 out of 16) in our shortlist (Fig. 1d). Our analyses of histone marks, P300 occupancy, and chromatin accessibility revealed that while many trophoblast-expressed ERVs are predicted to function as both enhancers and promoters, the MLT families expressed in trophoblasts are predominantly predicted to act as enhancers rather than promoters (Additional file 4: Fig. S1). Of these 16 genes, four (Fig. 1d), were previously described as being PE dysregulated (although not known to be ERV-associated dysregulation), namely Cytochrome P450 Family 11 Subfamily A Member 1 (*CYP11A1*), Chorionic Gonadotropin Alpha (*CGA*), Corticotropin-Releasing Hormone (CRH), and Sialic Acid Binding Ig Like Lectin 6 (*SIGLEC6*) [40, 46–48].

**Fig. 2:**
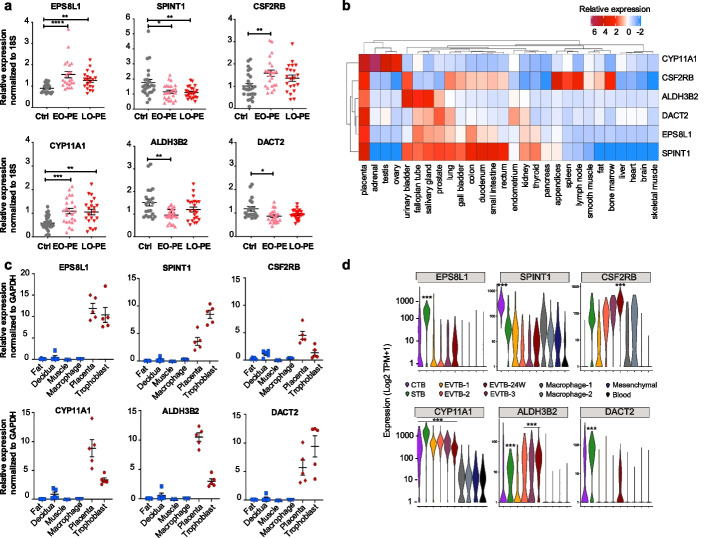
Dysregulation of the six short-listed ERV-driven trophoblast-specific genes in pre-eclampsia. **a** qPCR (normalized to 18s) confirmed increased mRNA levels of *CYP11A1*, *EPS8L1* and *CSF2RB* and downregulated expression of *SPINT1*, *ALDH3B2*, and *DACT2* in early-onset PE placental samples of Oslo-cohort compared with healthy controls (mean ± SEM; Control *n* = 27; Early-onset (EO)-PE *n* = 24; Late-onset (LO)-PE: *n* = 23) (*P ≤ 0.05, **P ≤ 0.01, ***P ≤ 0.001, ****P ≤ 0.0001; Kruskal–Wallis test; Dunn’s multiple comparison or one-way ANOVA; Dunnett’s multiple comparison test). **b** Analysis of pilot GTEx data [49] for the five novel candidate genes (*EPS8L1*, *CSF2RB*, *SPINT1*, *ALDH3B2*, and *DACT2)* and *CYP11A1*. The heatmap shows the relative expression of the candidate genes in the different human tissues. Note that the candidate genes are highly expressed in the placenta. **c** qPCR (normalized to GAPDH) confirmed higher transcript levels of *CYP11A1*, *EPS8L1*, *CSF2RB*, *SPINT1*, *ALDH3B2*, and *DACT2* in placental tissue and trophoblast cells compared with other pregnancy-related tissues. Transcripts of *CYP11A1*, *CSF2RB*, and *ALDH3B2* were also detected in decidua but to a lesser extent (*n* = 5 in each group, mean ± SEM, *P* ≤ 0.01; Kruskal–Wallis test; Dunn’s multiple comparison test or one-way ANOVA; Dunnett’s multiple comparison test). **d** Expression of individual candidate genes in the single-cell transcriptome of human 8 and 24 week (W) placental tissue [50]. Violin plots showing the distribution of gene expression in single cells (Log2 Transcripts per million, TPM + 1) across trophoblast lines (CTB: Cytotrophoblast_8W, STB: Syncytiotrophoblast_8W, EVTB: Extravillous trophoblast_8W, distinct populations—EVTB1-3: 8 W and 24 W, Macrophages-1 and Hofbauer cells (Macrophage-2), Mesenchymal cells, Blood). Note: The indicated candidate genes are significantly (***FDR < 2e − 7) upregulated in either one or all trophoblast lines compared to the other cell types

To test the differential expression of the identified ERV-regulated candidate genes in a broader range of PE patients, we performed qPCR on human placental tissue samples from the Oslo Cohort. We analyzed the expression of the previously reported CYP11A1 and the twelve new candidate genes. Of the twelve, qPCR confirmed significant dysregulation of five genes (*ALDH3B2, CSF2RB*, *DACT2*, *EPS8L1*, and *SPINT1*) (Fig. 2a) in early-stage PE patients compared to healthy samples. *CYP11A1*, *CSF2RB* (Colony Stimulating Factor 2 Receptor Beta), and *EPS8L1* were upregulated while ALDH3B2 (aldehyde dehydrogenase 3 B2), *DACT2* (Dishevelled Binding Antagonist of Beta Catenin), and *SPINT1* (Serine Peptidase Inhibitor Kunitz 1) were downregulated (Fig. 2a). Differential gene expression of *C1QTNF6*, *KIF23*, *RAD51*, *PLEKHA8*, *NRK*, *PHYHIPL*, and *SLC22A11* was not significant (Additional file 4: Fig. S2), leaving 9 candidate genes as having a role in PE, four previously defined (*CYP11A1*, *CGA*, *CRH*, and *SIGLEC6*) and five novel (*ALDH3B2*, *CSF2RB*, *SPINT1*, *DACT2*, and *EPS8L1*).

### ERV-associated genes dysregulated in preeclampsia have placenta-specific expression

Are CYP11A1 and the five novel shortlisted PE dysregulated genes (PE genes thereafter) placental-specific in their expression? The merged expression of the six PE genes was the highest in normal placental tissue compared with 38 other human body tissues and GTEx (Fig. 2b), suggesting their placenta-specificity. To confirm this, we performed qPCR on human pregnancy-associated tissue samples: maternal muscle, fat, decidua, macrophages, placental tissue, and primary human trophoblast. This confirmed the enriched expression of the six genes in the placental tissue and primary human trophoblast cells (Fig. 2c). Transcripts of *ALDH3B2*, *CSF2RB*, and *CYP11A1* were also detected in the decidua but at lower levels (Fig. 2c).

Data mining of single- cell (sc) RNA-seq data of human placental tissue from 8th and 24th weeks of pregnancy (first and second trimester, respectively) [50] (GSE89497) finds that expression of all six genes was enriched in at least one of the sub-types of trophoblasts. *CYP11A1*, *DACT2*, and *EPS8L1* are expressed most abundantly in the syncytiotrophoblast (STBs) of the first trimester (8 week). Expression of SPINT1, CYP11A1, and CSF2RB were expressed in most of the trophoblast sub-types, whereas expression of EPS8L1, DACT2, and ALDH3B2 was more specific (Fig. 2d).

### MLT1 derived trophoblast-specific enhancers regulate PE candidate genes

**Fig. 3:**
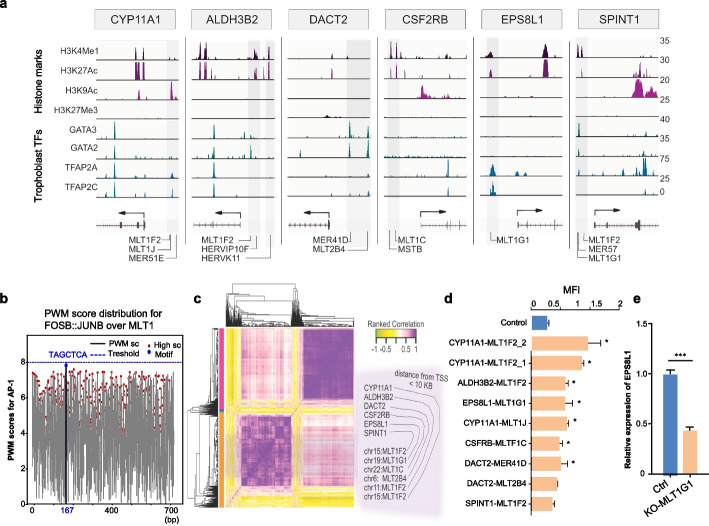
Functional validation of the ERV-derived enhancers driving the six shortlisted genes. **a** Retrovirus LTRs with active histone marks in trophoblast function as alternative regulatory elements of the six selected genes. Integrative Genome Visualization (IGV) shows raw ChIP-seq signals of various histone modifications and transcription factors (TFs) around the transcriptional start sites (TSSs, +/− 15 KB of TSSs) of the selected genes. ChIP-seq signal in trophoblast cells for active enhancers (e.g., H3K4Me1, H3K27Ac, and H3K9Ac [19, 55]), Polycomb repressor (e.g., H3K27Me3 [55]), and master TFs determining trophoblast lineages (e.g., GATA2, GATA3, TFAP2A, and TFAP2C [52]) visualized to characterize the regulatory regions of the six genes. Arrows indicate the TSSs and the direction of transcription of each gene. Shaded boxes mark the repeat masked endogenous retroviral long terminal repeat (ERV-LTR) sequences with active chromatin status around the genes. **b** Distribution of position weight matrix (PWM) scores for FOSB::JUNB (AP-1) in the MLT1 reference sequence from Repbase. The *x*-axis represents the position (base pair) within the sequence, while the *y*-axis indicates the PWM score. The dotted line signifies the minimum threshold score (8) for a significant motif match. The red dots represent positions in the sequence where the PWM score exceeds this threshold, indicating potential binding sites. These positions represent regions with strong similarity to the transcription factor’s binding preferences, though not necessarily exact matches to the canonical “TGACTCA” motif. Scores below the threshold indicate sequences with mismatches or gaps relative to the optimal binding site. **c** Characterization of the six ERV-gene pairs. The heatmap displays ranked Spearman’s correlation coefficients between the expression levels of transposons and protein-coding genes, inferred from RNA-seq data across healthy and PE placenta samples. The pairwise correlation matrix was clustered using *k*-means clustering (Euclidean distance), and the resulting cluster assignments are indicated by the color bars. The six candidate genes and their putative ERV-derived CREs are highlighted, as they co-occur within the same correlation network (also refer the Fig. 3a). **d** Determination of the functional enhancer potential of the ERV-LTR sequences identified as potential alternative regulatory sequences of the candidate genes. Bar-plot showing the Median Florescence Intensity (MFI) values calculated by normalizing the GFP-reporter signal with the mCherry signal (internal control) for the nine LTR constructs (shown in orange) as compared to control (construct w/o LTR, shown in blue) (*n* = 4; mean ± SEM; *P ≤ 0.05; Unpaired *t*-test). **e** Normalized (to GAPDH) mRNA expression levels of EPS8L1 in homozygous MLT1(G1) deletion colonies (CRISPR/Cas9). Data represent the mean ± SEM from three biological replicates, each with three technical replicates (*n*= 3, ***P ≤ 0.001; Unpaired *t-*test)

To consider the *cis-*regulatory effect of ERV-derived sequences 5’ upstream of the six PE genes, we mined ChIP-seq data generated in differentiated trophoblasts [51]. In addition to histone mark analysis (H3K27Ac, H3K4Me1, H3K27Me3) of these six genomic loci, we determined transcription factor binding for a set of selected transcription factors (GATA3, GATA2, TFAP2A, and TFAP2C), known to regulate early trophoblast progenitor differentiation [52, 53] (Fig. 3a). We also analyzed CUT&Tag data for JUN, JUND transcription factors [17], and the position weight matrix (PWM) scores for FOS::JUN, the subunits of the activator protein-1 (AP-1) complex (Fig. 3b and S3a) in the MLT1 reference sequence (RepBase). AP-1 is implicated in enhancer binding and regulating trophoblast differentiation and invasion [53, 54]. We found at least one of the four trophectoderm-specific TFs showed binding enrichment along with active enhancer marks (Fig. 3a), suggesting that these ERVs function as cell type-specific enhancers for their downstream neighboring genes, as suggested by their co-expression patterns (Fig. 3c). The enrichment of JUN::FOS (AP-1 complex) (Fig. 3b) suggests that these upstream regulatory sequences of the candidate genes are in global transcriptional regulatory networks targeted by the AP-1 complex.

To demonstrate the functionality of the ERV-derived sequences as enhancers located upstream of the six PE genes, an in vitro GFP reporter assay was established in BeWo cells. The ERV sequences were PCR-amplified from human placental genomic DNA and cloned upstream of the minimal CAGs promoter sequence (Fig. 3c). To dissect the multiple ERVs upstream of *CYP11A1* and *DACT2* genes, we generated individual reporter constructs. While the enhancer assay revealed an ERV1-MER41D-driven reporter signal at the *DACT2* locus, the highest reporter signals were for two ERV3-MLT1(F2) elements, both amplified from the upstream region of *CYP11A1* (Fig. 3d and Additional file 4: FigS2b). The *CYP11A1* locus has a third MLT1 sequence (variant MLT1(J)) tested positive in the assay, suggesting that trophoblast-specific gene expression of this gene is tuned by different subfamilies of ERV-derived enhancers (Fig. 3d). In addition, we observe reporter signals for MLT1(F2), (G1), and (1C) variants located at the ALDH3B2, EPS8L1, and CSF2RB loci, respectively. We also found MLT1(F2) and MLT1(B) upstream of the previously described PE-dysregulated genes CGA, CRH, and SIGLEC6. This approach revealed an enrichment of ERV-derived enhancers upstream of PE genes of the ERV3-MLT1 family. Finally, we knocked out the MLT1(G1) element with CRISPR/Cas9 editing in BeWo cells (Fig. S4a-c, Additional file 5). The qPCR analysis supports the role of MLT1G1 as a key regulator of *EPS8L1* transcription and suggests that its deletion directly affects gene expression (Fig. 3e).

**Fig. 4:**
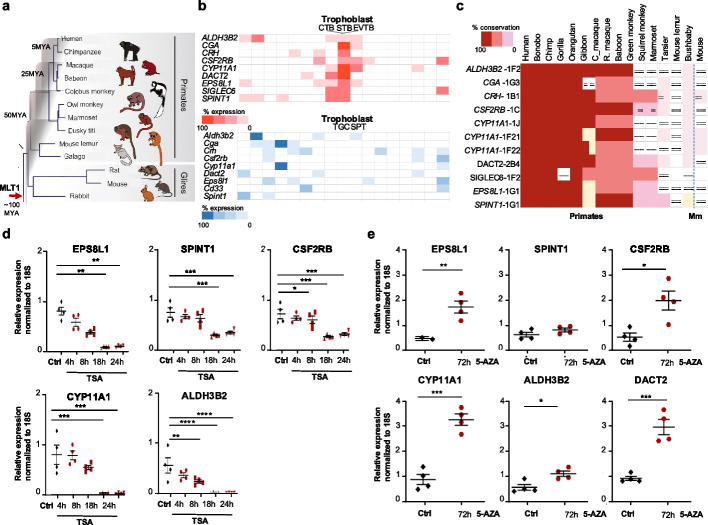
ERV3-MLT1 confers trophoblast-specific expression to the selected PE genes in humans and makes them sensitive to epigenetic changes. **a** The ancient MLT1 retrovirus was integrated around ~ 100 MYA before the primate/rodent split and generated multiple subfamilies throughout primate evolution. **b** Cell type specificity of gene expression of the nine shortlisted genes in human (top) and mice (bottom). Note that the shortlisted nine PE candidate genes exhibit trophoblast-specific expression in human, whereas their mouse orthologues have low specificity and expressed in multiple cell types. Cytotrophoblast (CTB), Syntiotrophoblast (STB), Extravillous trophoblast (EVTB). Trophoblast giant cells (TGC), Spongiotrophoblast (SPT). For the list of investigated cell types see also “Methods” and Additional file 13. Note that the *SIGLEC6* in mouse is called *Cd33*. **c** The heatmap shows the evolutionary conservation analysis of the MLT1/2 copies inserted upstream of the shortlisted PE dysregulated genes in 15 primate genomes and in mice (Mm). The analysis is based on the MultiZ alignment generated by the UCSC Genome Browser Group. The red intensity reflects the degree of sequence conservation relative to the human genome, while white indicates no detectable MLT1/2 LTR sequence in the corresponding species. Alignment gaps were interpreted following UCSC Genome Browser guidelines, to account for lineage-specific events or evolutionary divergence. Alignment gaps were interpreted following UCSC Genome Browser guidelines, to account for lineage-specific events or evolutionary divergence: *Single line gap*: No bases are aligned in the comparator species. This may reflect a lineage-specific insertion in the human genome or a deletion in the comparator species. *Double line gap*: Bases are present in the comparator species but are unalignable. This may result from significant evolutionary divergence or independent indels between the aligned blocks. *Beige colored gap*: Missing sequence in the genome assembly, as indicated by UCSC alignment tracks. See also Additional file 14. Note that (i) the MLT1/2 elements were inserted at different points during evolution; (ii) none of the MLT1/2 LTR sequences (0/11) are detectable in mouse lemur, whereas (iii) 8/11 loci are found in bushbaby, a pattern inconsistent with the expected phylogeny; (iv) 5/11 loci are not fixed in gibbon. These findings may reflect either low genome assembly quality (false negatives) or lineage-specific integration events; (v) 3/11 loci are also detectable in mouse, suggesting deeper evolutionary conservation for these elements. (vi) MLT1(G1), located upstream of *EPS8L1*, is detectable in marmoset but absent in more distantly related species, suggesting a relatively recent insertion event, rather than incomplete detection due to technical limitations. The coordinates of the analyzed MLT1/2 LTRs and the corresponding alignments are now included in Additional file 14. **d** qPCR analysis of the PE dysregulated genes (identified in this study) following Trichostatin A (TSA) treatment in trophoblast cells (*n* = 4; mean ± SEM, *P ≤ 0.05, **P ≤ 0.01, ***P ≤ 0.001, ****P ≤ 0.0001; one-way ANOVA; Dunnett’s multiple comparison test or Kruskal–Wallis test; Dunn’s multiple comparison test). Note that DACT2 expression levels were not detectable in the treated cells (not shown). **e** qPCR analysis of the PE dysregulated genes (identified in this study) following 5-AZA-2 deoxicytidine (5-AZA-dC) treatment in trophoblast cells (*n* = 4; mean ± SEM, *P* ≤ 0.05, **P ≤ 0.01, ***P ≤ 0.001; Unpaired *t*-test)

This family was integrated/fixed prior to the rodent-primate split (Fig. 4a) and subsequently gave rise to several subfamilies [56]. Do the MLT1 regulated genes thus have similar expression in mice, with the CRE having a conserved functionality? While all the nine PE genes (4 reported and 5 novel) have trophoblast specific expression in humans (Figs. 2c,d and 4b), they exhibit different expression patterns in mice (Fig. 4b and Additional file 6). Assuming that MLT1-derived enhancers confer cell type-specific expression to these genes, we analyzed the phylogenetic conservation of the MLT1/2 loci upstream of the nine genes (11 loci). The analysis revealed that most of the MLT1/2 copies in question have a patchy conservation in primates (Fig. 4c and Additional file 7).

### Epigenetic disturbances dysregulate MLT1-governed gene expression

The observed dysregulation of multiple MLT1-regulated genes in PE suggests that epigenetic disturbances may contribute to the genome-wide changes in ERV-regulated gene expression. To investigate this possibility, we first analyzed CpG methylation at enhancer regions using bisulphite sequencing in placental genomic DNA from PE patients and healthy controls (Additional file 8). However, due to the highly repetitive and homologous nature of MLT1 elements, we encountered technical limitations in achieving specific and efficient amplification from bisulphite-treated genomic DNA. Consequently, we were only able to reliably amplify and sequence a single CpG site located within the MLT1F2-2 element upstream of CYP11A1, which contains one CpG dinucleotide within the amplified region. This site was demethylated in PE placentas but remained methylated in healthy controls (Additional file 4: Fig. S5).

To complement this limited locus-specific methylation analysis, we assessed the functional impact of epigenetic modifiers in BeWo cells. Specifically, we treated the cells with 5-Aza-2′-deoxycytidine (5-AZA-dC), a DNA demethylating agent, and Trichostatin A (TSA), a histone deacetylase inhibitor. We then measured the expression levels of six PE-dysregulated genes by qPCR. Treatment with 5-AZA-dC significantly upregulated CYP11A1, EPS8L1, DACT2, and CSF2RB, while TSA treatment consistently downregulated all six tested ERV-derived enhancer-driven PE genes (Fig. 4d and e). These findings are consistent with, but do not conclusively demonstrate, the hypothesis that epigenetic dysregulation contributes to aberrant ERV-driven gene expression in PE.

### The PE dysregulated EPS8L1 is a trophoblast-specific paralog of the Epidermal Growth Factor Receptor 8 (EPS8)

Four of the five novel PE dysregulated genes are relatively well-characterized and implicated in various biological processes, including detoxification (ALDH3B2), interleukin signalling (CSF2RB), regulation of the proteolytic activation of the hepatocyte growth factor (HGF) in injured tissues (SPINT1), and developmental signalling (DACT2). In contrast, the function of the dysregulated EPS8-Like 1 (EPS8L1) gene is unknown.

Based on ~ 50% amino acid identity EPS8L1 is a paralog of EPS8 (Epidermal Growth Factor Receptor Pathway Substrate 8), frequently associated with cancer (i.e., Ref [57]). The EPS8 and EPS8L1 genomic loci differ with respect to a primate-specific MLT1(G1) element located upstream of the EPS8L1 gene (Fig. 4c). This particular MLT1(G1) copy is not highly conserved, and orthologous genomic regions harboring MLT1(G1) upstream of the EPS8L1 gene can only be identified in marmosets (Fig. 4c). EPS8 is predominantly expressed in the uterus, arteries, and fat cells (GTEx portal, [49]), but not in the placenta/trophoblast, and is not dysregulated in PE patients (Additional file 4: Fig. S2). To determine whether EPS8L1 activity might be causally involved in PE we provide both omic and experimental analyses.

### EPS8L1 is trophoblast-specific and interacts with signalling and stress response networks

**Fig. 5:**
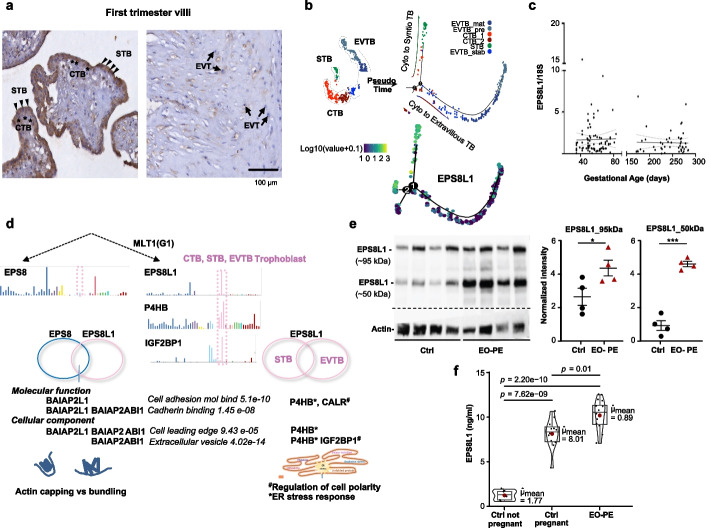
EPS8L1 is trophoblast-specific and is expressed throughout gestation. **a** Immunohistochemical staining on first trimester villi and term placenta (left and right panels, respectively) shows the expression and localization of EPS8L1 in the syncytiotrophoblast (STB), villous cytotrophoblast (CTB), and extravillous trophoblast (EVTB) were depicted with black triangles, asterisk, and arrows, respectively. Scale bar = 100 µm. Trophoblast-specific expression of EPS8L1. The arrows show the expression EPS8L1 in cytotrophoblast cells (CTB), syncytiotrophoblast (STB), extravillous trophoblast cells (EVTBs). **b** Pseudotime feature plot illustrating the expression of EPS8L1 along the differentiation trajectory of the three major trophoblast lineages (CTB, STB, EVTB). Upper panel: the three major cell types CTB, STB, and EVTB from the trophoblast lineages are shown by Monocle2 single-cell trajectory analysis and cells are ordered along an artificial temporal continuum using the top 1000 differentially expressed genes between them. Each dot is a single cell and each color is coding for their corresponding lineage. The values shown are the pseudotime values generated by Monocle2. Lower panel: Feature plot based on the above plot shows the expression dynamic of EPS8L1. Cells ranging from blue (low expression) to yellow (high expression) of EPS8L1 are representing the gain of EPS8L1 expression pattern during the lineage progression of STB. Note that EPS8L1 is primarily expressed in CTB cells that are committed to the STB lineage and further it marks the STB. **c** Placental EPS8L1 expression over gestation. qPCR analysis to detect EPS8L1 from early gestation (*n* = 92) and term (*n* = 49) placentas derived from healthy pregnancies (Graz-Aachen cohort, no self-reported medical history, no smoking habit, > 18 years old). Note that the simple linear regression for expression levels to gestational age was not significant from zero (linear regression line and confidence band shown, alpha was set at *P* = 0.05). **d** Functional diversification of EPS8 family member EPS8 (left) and EPS8l1 (right) due to the MLT1(G1) enhancer integrated upstream of EPS8L1. Cell type-specific transcription of selected genes (adopted from Human Protein Atlas). Trophoblast-specific transcription is boxed (dashed pink). Common protein–protein interacting (PPI) partners identified from the overlapping proteomes of (Left) EPS8 and EPS8L1 (blue and pink circles); (Right) EPS8L1 between STB and EVTB (pink circles). (Bottom) Ontology analysis of selected genes. Note that while EPS8 is predominantly expressed in glandular epithelial cells, EPS8L1 is highly expressed in trophoblast cells. In this trophoblast niche (where EPS8 shows minimal expression), EPS8L1 forms PPI with specific co-factors that are themselves enriched in the trophoblast, suggesting niche-specific functional specialization. Shared PPI partners between EPS8 and EPS8L1 are involved in actin cupping and bundling. Specific PPI partners of EPS8L1 are involved in (^*^) *Regulation of cell polarity* and (^#^) *ER stress response*. **e** Western blot analysis reveals the upregulation of two protein isoforms of EPS8L1 in the placenta from early-onset (EO-PE) (*n* = 4; triangles, Oslo cohort) compared to controls (*n* = 4, circles). (Right panel) Quantification of the EPS8L1 isoforms in EO-PE samples. The relative amounts were determined as the ratio of each isoform band relative to the loading control (actin) (mean ± SEM; *P ≤ 0.05, ***P ≤ 0.001; Unpaired *t*-test). **f** Quantification of the secreted EPS8L1 isoforms in EO-PE samples. Enzyme-linked immunosorbent assay (ELISA) detects the upregulation of the secreted EPS8L1 protein in the plasma of EO-PE patients (*n* = 12; Manchester cohort). The matching controls derived from 24 to 28^th^ week of gestation (*n* = 12). In addition, blood samples were collected from healthy, non-pregnant women (*n* = 6; NPF cohort) to confirm that EPS8L1 expression is placental. Each sample was tested in duplicate. Data are presented as mean ± SEM and were analyzed with the Pairwise test: Games-Howell. Bars shown: significant. Note the low expression of EPS8L1 in non-pregnant samples

Functionally, EPS8 is known to link growth factor stimulation to actin reorganization [58]. To start addressing whether EPS8L1 may similarly function in trophoblast, we used immuno-histochemical staining on healthy human placental tissue at first trimester placental villi (Fig. 5a). In agreement with single-cell transcriptome data (Fig. 2d), EPS8L1 expression was detectable in various trophoblast cell types (e.g., CTBs, EVTBs, STB) with predominant expression in STBs (Fig. 5a). Other tissues and cells (e.g., fat, blood, muscle, and macrophages) showed very little expression of EPS8L1, and no expression was detected in the maternal decidua (Fig. 2c,d). Pseudotime analysis of the single-cell transcriptome of the human placenta revealed that EPS8L1 is expressed in CTBs and, after differentiation, primarily in STBs and only sporadically in EVTBs (Fig. 5b). qPCR on human placental samples (*n* = 141) revealed that EPS8L1 is expressed extensively through pregnancy (Fig. 5c). We conclude that EPS8L1 is extensively expressed through pregnancy.

How, if at all, might EPS8L1 affect cell type-specific protein–protein interactions (PPIs)? As EPS8L1 is primarily expressed in STB, but also detectable in extravillous trophoblast (EVTB), we determined its interactome in cultured cell lines (both BeWo, STB-type and SGHPL-4, EVTB-type) (Additional file 4: Fig S6 and Additional file 9). We expressed a HA-tagged version of EPS8L1 (Additional file 4: Fig. S9) and performed mass spectrometry analysis following HA-based affinity capture. This identified 30 interacting proteins in BeWo cells and 106 proteins in SGHPL-4 cells, with an overlap of 12 proteins (-Log (*P*-value > 1.25 and see Supplementary Methods for further filtering) (Additional file 8: Table S3). We also compared the data of EPS8L1 with the interactome of its paralog EPS8 (BioGrid).

In the overlapping interactome of EPS8L1 and its paralog EPS8 are the signal transducers ABI1 (ABl interactor 1) and BAIAP2 (BAR/IMD Domain Containing Adaptor Protein 2) (molecular function: cadherin binding), indicating a conserved signal adaptor function (Fig. 5d, Additional file 8: Table S3). The presence of these signal transducers in the interactome of EPS8L1 suggests that, similar to EPS8, the signal adaptor function regulates the dynamics and architecture of the actin cytoskeleton (e.g., actin capping vs. actin bundling) (Fig. 5d). However, this regulatory function of actin cytoskeleton structural changes was recruited to trophoblasts via MLT1(G1)-directed EPS8L1. In the trophoblast niche, EPS8L1 established stable protein–protein interactions, as evidenced by the interaction partners of EPS8L1 that are specifically enriched in the trophoblast (e.g., *IGF2BP1* (insulin like growth factor 2 binding protein 1), *P4HB* (prolyl 4 hydroxylase beta), *CALR *(Calrectulin) (Fig. 5d).

The identification of EPS8L1 with its PPI partners in the enrichment categories of *extracellular vesicles*, *cadherin binding*, and *cell adhesion molecule binding* suggests that EPS8L1 exhibits molecular properties that are important for communication between two functionally distinct trophoblast cell types (e.g., STB and EVTB). In the overlapping PPI cluster of EPS8L1 of the two cell types, the enriched KEGGs signalling pathway is *protein processing in the ER*. The presence of *TXNDC5* (Thioredoxin Domain Containing 5), which is involved in the regulation of ER homeostasis, cell proliferation, and angiogenesis [59], and *PRDX1* (Peroxiredoxin 1), may confer EPS8L1 the ability to recognize and respond to cellular stress (Additional file 9: Table S3).

### Secreted EPS8L1 isoforms are elevated in PE and correlate with disease severity

EPS8L1 is predicted to have a subcellular localization in the cytosol, whereas EPS8 resides in the plasma membrane (https://www.proteinatlas.org). Furthermore, analysis of the six putative protein-coding isoforms of EPS8L1 (Additional file 9: Table S4) identified a transmembrane domain in one isoform EPS8L1-205 that has a unique exon 1 (Additional file 4: Fig. S7 and S8a). To determine whether the EPS8L1 isoform(s) are secreted in the maternal blood, we also performed western blot analysis on maternal plasma samples. The western blot identified two secreted protein isoforms (~ 72 kDa; ~ 50 kDa) (Additional File 4: Fig. S8b) that most likely correspond to EPS8L1-203 (659 aa) and either EPS8L1-207 (428 aa) or EPS8L1-209 (409 aa) that lack exons 3 or 4 and the SH3 (SRC homology) or PTB (Phosphotyrosine-binding) domains compared to full-length EPS8L1-201 (Additional file 4: Fig. S7).

That there are isoforms in maternal circulation is potentially of importance as it may aid plasma-based diagnostics. We therefore sought to better characterize this blood presence. Notably, two placental EPS8L1 isoforms were upregulated in the EO-PE patients compared to healthy controls (*n* = 4 vs. 4) (Fig. 5e), early onset disease being the most severe in outcome. The secreted isoforms were detectable by ELISA on maternal plasma (*n* = 12 vs. 12) collected in the Manchester cohort between 24 and 28 weeks of gestation from healthy controls and EO-PE patients, while EPS8L1 protein levels were low in sera from non-pregnant women (*n* = 6) (Fig. 5f). The assay detected upregulation of EPS8L1 in the blood of EO-PE patients upon PE onset (24–28 weeks) compared to same gestational age healthy controls (Fig. 5f).

**Fig. 6:**
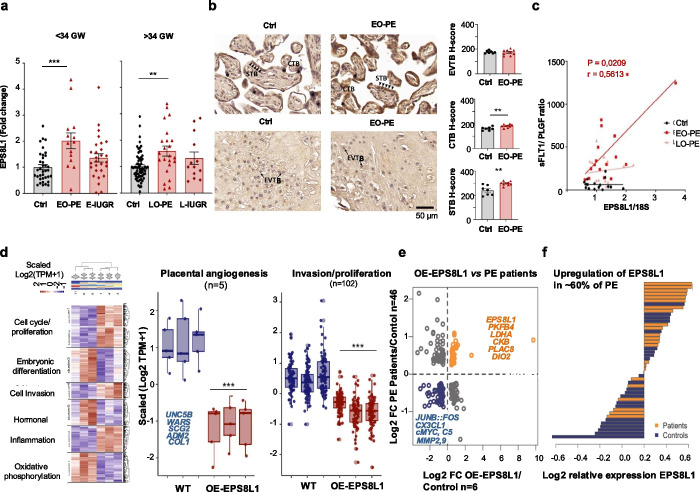
The in vitro overdosed EPS8L1 expression model mimics the transcriptome of PE patients. **a** EPS8L1 is specifically dysregulated in PE patients. qPCR specifically detects an increased EPS8L1 mRNA level in placentas of an early-onset PE (Charite/Aachen cohort; early-onset PE (*n* = 14) and late-onset PE patients (*n* = 23) compared to gestational-age matched healthy controls; control early (*n* = 32), control late (*n* = 65) (mean ± SEM; **P ≤ 0.01, ***P ≤ 0.001; Kruskal–Wallis test; Dunn’s multiple comparisons test). No significant differences were found in IUGR patients (IUGR early (*n* = 28), IUGR late (*n* = 13) as compared to the healthy controls (mean ± SEM; Kruskal–Wallis test; Dunn’s multiple comparison test). **b** EPS8L1 expression and localization in human placenta tissues of normal pregnancy and early-onset PE. Immunohistochemical (IHC) staining of EPS8L1 in term placenta tissue of normal pregnancy (control, gestational weeks mean 31.5 ± 3.33) and early-onset PE (gestational weeks mean 29.40 ± 2.67). The expression and localization of EPS8L1 in the syncytiotrophoblast (STB), villous cytotrophoblast (CTB), and extravillous trophoblast (EVTB) were shown with black triangles, asterisk, and arrows, respectively. Scale bar = 50 µm. (Right) Quantification of the expression of EPS8L1 in different types of trophoblast cells indicated by H-score. Note that the EO-PE patients showed significant increase in EPS8L1 expression levels in STB and CTB (*n* = 7 vs. 9, mean ± SEM; **P ≤ 0.01; Unpaired *t*-test). In contrast, no difference in the expression was found in EVTB of EO-PE patients as compared to the controls. **c** EPS8L1 expression correlates with the PE prognostic biomarker (sFLT1/PIGF ratio). Serum soluble fms-related tyrosine kinase-1 (sFLT1) and serum placental growth factor (PlGF). Oslo Cohort; EO-PE (dark red line) (*n* = 16), *r* = 0.5613, *P* = 0.0209; LO-PE (red line) (*n* = 16), *r* = 0.08529, *P* = 0.7545; Control (black line) (*n* = 17), *r* = − 0.1152, *P* = 0.6595; Spearman’s rank correlation. **d** Differentially expressed genes (DEGs, FDR < 0.05) in three replicates of EPS8L1-OE vs controls in SGHPl-4 cells is shown at scaled Log2 (TPM + 1) level. Seven clusters of DEGs are shown on the heatmap. Genes in Clusters 1, 2, 4, and 6 were downregulated, whereas clusters 3, 5, and 7 represent the upregulated gene sets upon EPS8L1 overexpression in trophoblast cells. Jittered boxplots represent the pairwise comparison of scaled expression (at Log2 scale) of genes. Every dot represents a single gene involved in pathways associated with (left) *angiogenesis* and (right) *invasion/proliferation*. **e** Comparison between DEGs in PE samples vs healthy controls and DEGs in OE-EPS8L1 SGHPL-4 cells overexpressing EPS8L1 vs controls. The top DEGs showing similar differential expression patterns involved in oxidative phosphorylation, angiogenesis, and invasion/proliferation are annotated. **f** Bar plots denoting the Log2 relative expression of EPS8L1 across the healthy (blue) and PE (orange) placenta samples. The relative expression of each sample is representing the difference of its EPS8L1 levels (normalized Log2 expression) relative to the mean value of EPS8L1 across the healthy and PE samples (*n* = 46). Note that around 60*–*65% of PE samples display higher expression of EPS8L1

To determine how specific the upregulation of EPS8L1 is to PE patients, we analyzed placental EPS8L1 levels in merged pool of additional cohorts (Aachen) that includes samples taken from patients with IUGR (intrauterine growth retardation). qPCR supported an elevated placental expression of EPS8L1 transcripts in early-onset PE patients (Fig. 6a). In contrast, we detected no significant differences in the samples derived from the IUGR patients of Charité-Aachen cohort (Fig. 6a). Staining placental tissue from healthy pregnancies and from early-stage PE (Essen cohort) reveals that in EO-PE placenta, EPS8L1 was differentially overexpressed only in STBs (*P* = 0.0016) and in CTBs (*P* = 0.0059), whereas no difference was observed in EVTBs compared to the control group (*P* = 0.7782) (Fig. 6b).

The circulating maternal angiogenic proteins originate mainly from the placenta and are measurable in maternal plasma. Currently, the ratio of sFLT1/PlGF (soluble FMS-like tyrosine kinase receptor-1/placental growth factor) is used as a prognostic biomarker for PE [60]. We found that placental EPS8L1 expression is positively correlated with the sFLT1/PlGF ratio in early PE patients (Fig. 6c). We do not know what, if any, effects the form (or forms) in maternal circulation might have on maternal physiology.

### Functional consequences of EPS8L1 overexpression in trophoblasts

To determine the cellular effects of EPS8L1, we created an in vitro EPS8L1 overexpression model using the *Sleeping Beauty* (SB100X) transposon system (Fig. S6) [61] in SGHPL-4 cells derived from the first trimester EVTB (Additional file 4: Fig. S9). RNA-seq was performed on these samples (OE-EPS8L1_SGHPL-4) and WT_SGHPL-4 controls [45].

Differential gene expression (DEG) analysis revealed 827 significant DEGs (adjusted *P* value < 0.05, and log2foldChange > 1) (Additional file 11: Table S5). The downregulated genes (615/827) were involved in biological processes such as *cell cycle/proliferation*, *cell invasion*, and *angiogenesis* in the placenta, whereas genes enriched in *oxidative phosphorylation* were upregulated (210/827) (Fig. 6d). To determine how closely our in vitro system mimics the EPS8L1 overexpression phenotype in PE patients, we compared the transcriptome of OE-EPS8L1_SGHPL-4 cells with transcriptome data from PE patients [31]. Transcriptomes of PE patients were significantly correlated with those of our OE-EPS8L1_SGHPL-4 cells (Fig. 6e,f). After overexpression of EPS8L1, both in vitro and in the patients, the significantly dysregulated genes included matrix metalloproteinases (MMP2, MMP9) (cell invasion), c-MYC (cell proliferation), and LDHA (oxidative phosphorylation) (Fig. 6e). Downregulation of matrix metalloproteinases and c-MYC (confirmed by qPCR) (Additional file 4: Fig. S10) is consistent with impaired invasion of trophoblast cells. Among the downregulated DEGs, we also found FOS and JUNB, the members of the dimeric transcription factor complex (AP-1 complex), known to regulate trophoblast invasion [53]. We have knocked-out (KO) EPS8L1 in both BeWo and SGHPL-4 cells (Additional file 4: Fig. S11 and Additional file 5), but the KO EPS8L1 cells are not viable. The SGHPL-4 cells were unable to grow at all, whereas the BeWo cells could survive 5–7 days of in vitro culture but eventually died, suggesting an essential function of EPS8L1 in human trophoblast. The same cells transfected with CRISPR-Cas9 but no guide RNA were without evidence phenotype.

**Fig. 7:**
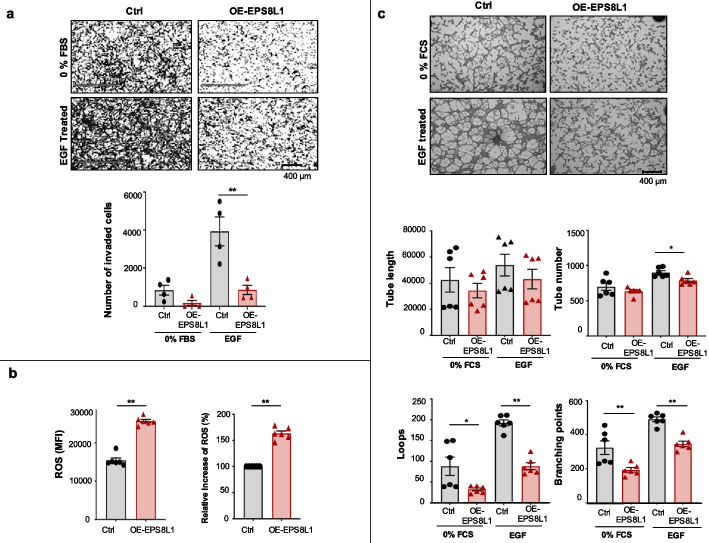
The in vitro overdosed EPS8L1 expression model of PE. OE-EPS8L1 cells form less tube-like structures, are less invasive, and have higher oxidative stress. **a** OE-EPS8L1 cells are less invasive. Matrigel-based *transwell invasion* assay showing reduced invasion capacity of OE-EPS8L1 SGHPL-4 cells as compared to control cells. Note that EPS8L1 overexpression reduces trophoblast invasion in both untreated (0% FBS) and treated (EGF) conditions. (*n* = 4, mean ± SEM, **P ≤ 0.01; Unpaired *t*-test). The positive control of the assay was EGF treatment (10 ng/mL), whereas 0% FBS was used as a negative control. (Bottom panel) Quantification of the *transwell invasion* assay. Representative images were taken from different areas of the well and ImageJ software was used to analyze and calculate the number of invaded cells (*n* = 4; mean ± SEM; **P ≤ 0.01; Shapiro–Wilk normality test followed by Unpaired *t*-test. **b** OE-EPS8L1 cells have higher oxidative stress. Reactive oxidative species (ROS) production is elevated upon EPS8L1 overexpression. ROS production in control and OE-EPS8L1 SGHPL-4 cells was determined using the dichlorodihydrofluorescein (DCF) assay. The fluorescent signal of the DCF is proportional to ROS production. Data is presented as a median fluorescent intensity (MFI) of the fluorescent signal. ROS production in OE-EPS8L1 SGHPl4 cells is elevated upon EPS8L1 overexpression in three biological replicates (*n* = 6; mean ± SEM; **P ≤ 0.01; Unpaired *t* test). **c** OE-EPS8L1 cells form less tube-like structures. The tube formation assay showing decreased capacity of OE-EPS8L1 SGHPL-4 cells to form endothelial-like tubes when compared to control cells. Control and OE-EPS8L1 SGHPL-4 cells were subjected to a medium containing 0% FCS and EGF on matrigel that induces endothelial-like tube formation. 0% FCS was used as a negative control. (Bottom panels) The *tube formation* assay was assessed using the Wimasis Image Analysis software by counting the numbers of branching points, tubes, loops, and tube length. Note that the total number of tubes, loops, and branching points were significantly decreased in OE-EPS8L1 SGHPL-4 cells (*n* = 6; mean ± SEM; *P ≤ 0.05, **P ≤ 0.01; Shapiro–Wilk normality test followed Mann–Whitney *U* test)

Based on the top dysregulated pathways identified by the transcriptome analysis (e.g., *cell invasion/proliferation, oxidative stress*, *angiogenesis*), we designed functional assays. To consider trophoblast invasion of SGHPL-4 cells, we used the *Transwell Invasion assay*. Compared to the unstimulated cells (0% FBS), stimulation by EGF readily increased the invasion of WT cells (Fig. 7a). Under similar conditions, by contrast, the number of invading OE-EPS8L1 cells was significantly less (*n* = 4, **P ≤ 0.01), when compared to control cells, suggesting that the elevated level of EPS8L1 overexpression reduced cell invasion of the trophoblasts.

To test whether EPS8L1 over-expression induces oxidative stress, we conducted an in vitro *DCFH-DA* (2’,7’- dichlorodihydrofluorescin diacetate) assay that measures intracellular reactive oxygen species (ROS). Our data indicated that overexpression of EPS8L1 increased the ROS level by more than 50% (*n* = 6, **P ≤ 0.01) (Fig. 7b).

Finally, to test whether EPS8L1 overdose affects angiogenesis we performed a *tube formation assay*. This assay revealed that the OE-EPS8L1 SGHPL-4 cells exhibited a significantly reduced number of tubes, loops, and branching points as compared to the WT controls (*n* = 6, *P ≤ 0.05; **P ≤ 0.01) (Fig. 7c).

## Discussion

### Dysregulated ERV3-derived (MLT1/2) trophoblast-specific enhancers in PE

Given the lineage-specific nature of ERVs, their ability to act as CREs, and prior evidence of their involvement in the evolutionary turnover of placentas, we hypothesized that they will play a role when dysregulated, in preeclampsia. We identified LTR-retrotransposons (or endogenous retroviral elements, ERVs) from all the three superfamilies of ERVs (ERV1,2,3) associated with strong enhancer like functioning in trophoblasts. There is an enrichment of ERV3-derived enhancers (MLT1/2) that were dysregulated in PE associated, with already known [40, 46, 47] as well as novel genes that have expression in the human trophoblasts. Experimental and omic data support the causal involvement of one such novel gene, EPS8L1 in PE, and, by extension, the functioning of the non-pathological human placenta. Indeed, further analysis over the genomic regions of the depicted set of ERVs identified transcription factor binding signals involved in trophoblast differentiation [51, 52]. These ERVs also were enriched in transcriptionally active enhancer marks (H3K4Me1), and their functionality was validated in the reporter assay.

An intriguing aspect of our results is that, while multiple ERV families were seen to potentially act as strong enhancers in trophoblast, in PE all ERVs belonged to the same family. This would be consistent with the hypothesis that this family uniquely might bind one TF that is dysregulated in PE. When we considered four TFs known to be relevant to placental function, each of our six genes showed evidence for binding to at least one (Fig. 3a), but no TF bound all six. The “one dysregulated TF” model thus is not supported, but we may be missing the important TF. We also do not exclude the possibility that other ERVs might function as CREs in PE, even though we found no such evidence. Our analysis trained sequences from known enhancers to derive those ERVs likely to be strong enhancers. Results may well differ if instead of enhancers we trained on CREs more broadly defined.

It is notable that our approach identified genes associated with placental development and PE that are not themselves highly lineage specific. *EPS8L1* is a case in point, Ensembl recognizing orthologs in fish, reptiles, birds, and mammals, but not in invertebrates, this being consistent with a duplication event early in vertebrate history. Similarly, while the ERV predates the mouse-human common ancestor, its effects are particular to sub-lineages. This is consistent with lineage-specific recruitment by serendipity. The resulting lineage-specificity suggests that a search for lineage-specific regulation is a desirable, if not necessary, component of any attempt to identify causes of lineage-specific traits and disorders. They also show that the lineage-specificity of the effects are uncoupled from the degree of lineage-specificity of the underlying substrates (ERV3).

### ERV-LTR-associated dysregulation of genes contributes to the heterogeneity of PE

What might our results mean for PE? First, the results support a previously unrecognized mode of dysregulation of genes previously known to be dysregulated in PE (such as CYP11A1). Second, while the diversity of cellular pathways that are affected is striking, this underscoring the complexity of PE, underpinning this diversity, the data suggest that a unifying feature of the ERV-LTR-associated dysregulation of genes in PE includes disturbed epigenetics. As these changes are detected early in pregnancy, the cause of epigenetic instability in even earlier pregnancy should be a focus of PE research.

Third, the detection of the dysregulation in early-onset PE is especially significant, the discovery of dysregulated EPS8L1 being particularly notable. In contrast to EPS8, EPS8L1 has secreted isoforms, detectable in maternal plasma. While non-pregnant women have very low levels of plasma EPS8L1, elevated EPS8L1 expression can be readily detected in many PE patients, including in the high-risk pregnancy cohort. EPS8L1 has a stable expression in the non-pathological human placenta and is independent of the gestational-age changes. Conversely, elevated levels of EPS8L1 correlated with classical markers of PE and were detectable in early-onset PE patients from four independent cohorts in around 60–65% of the PE-diagnosed patients. The dysregulated biological processes of *OE*-*EPS8L1* partially overlap with the OE-DLX5 phenotype of preeclampsia [31] supporting the view that PE is a heterogeneous disease [31, 62]. The upregulation of *EPS8L1* was specific to PE patients, and was not detectable in IUGR.

### EPS8L1 over-expression phenotype and the classical 2-stage model

The importance of the discovery of EPS8L1 as a trophoblast-specific gene dysregulated in PE goes beyond its potential application for EO-PE diagnostics. That EPS8L1 over-expression is associated with both weakened trophoblast invasion and increased oxidative stress in cell lines is important as both features are characteristic of PE [63, 64]. Its activity also augments the canonical model in which poor invasion leads to placental malperfusion and hence oxidative stress [36]. The oxidative stress is then seen as the downstream core phenotype of PE and the pathology in need of treatment. As the cell line over-expressing EPS8L1 cannot have oxidative stress owing to poor invasion (it is a cell line), its phenotype adds to the complexity of the development of the disease. While it is likely that poor invasion can lead to downstream oxidative stress, early over-expression of EPS8L1 is likely to also directly cause such stress prior to any failure of invasion. If so, it suggests an augmented model in which both oxidative stress and poor invasion occur in the early stages without the later necessarily causing the former, with causality for early-onset PE mediated by epigenetic disturbance prior to week 20. Such a revision reinforces the need to better understand the epigenetic regulation in PE and non-PE patients far prior to week 20. The discovery of EPS8L1 as potentially patho-physiologically important could support such analyses.

### Might maternal plasma levels of EPS8L1 aid early PE diagnosis?

Trajectory analysis indicates that EPS8L1 is already expressed in progenitor (stem-like) cytotrophoblasts (CTBs), suggesting a role in placental development, particularly during the early to mid-pregnancy window. Overexpression of EPS8L1 is detectable in both CTBs and STBs. Significantly, a soluble form of EPS8L1 was detectable in maternal plasma samples collected between 24 and 28 weeks of gestation. These findings raise the possibility that elevated EPS8L1 levels may support the early prediction of PE, potentially before the onset of clinical symptoms. Dysregulation of EPS8L1 could thus serve as an early biomarker for specific PE subtypes and may contribute to a broader biomarker panel for early PE detection. Future studies including plasma samples from earlier stages of pregnancy (e.g., before 16 weeks gestation, the current threshold for starting low-dose aspirin prophylaxis [34]) are desirable to test the predictive power of EPS8L1 with regard to the risk of early-onset PE. Whether other dysregulated genes associated with EO-PE are detectable in plasma (or urine) should also be further investigated.

## Conclusions

We conclude that ERV3-MLT1, which dates back to the common ancestor of mice and humans, exhibits a sporadic distribution among primates. It may serve as a trophoblast-specific cis-regulatory element (CRE) for several human genes and, when dysregulated, may contribute to the human-specific disorder PE. As EPS8L1 has a form in maternal circulation, it may have utility in diagnostics.

## Methods

### Identification of ERV-derived putative enhancers in the trophoblast

To identify ERV-derived putative enhancers in the trophoblast, we employed a one-dimensional convolutional neural network (1D CNN), optimized for analyzing DNA sequence data without the need for explicit feature selection [43, 44]. The training dataset included sequences experimentally validated as enhancers [65], while a negative set of non-enhancer sequences was also incorporated to improve model discrimination. Each sequence, whether derived from experimentally validated enhancers or ERV elements, was assigned a unique identifier and labelled (1 for enhancer, 0 for non-enhancer), then divided into training and testing sets. To generate input data for the model, ChIP-seq data were aligned using Bowtie2 and enriched peaks for histone modification marks were called with MACS2**.** Enrichment for H3K4me1 at transposable element (TE)/ERV coordinates was visualized using Deeptools (ComputeMatrix and PlotHeatmap). Additionally, GC content was computed for each sequence and incorporated as a feature in the model [42, 66]. Sequence annotations were extracted using RepBase to provide context for ERV family classification. This deep learning-based approach enabled the systematic identification of ERV-derived sequences with enhancer-like properties in the trophoblast. For further details on model architecture, training parameters, and evaluation, see [43, 44] and Additional File 1.

### Evolutionary conservation analysis of MLT1/2-derived functional enhancers

MLT1/2 elements (11 loci) identified as enhancers upstream of genes dysregulated in preeclampsia were analyzed for evolutionary conservation using multiple genome alignments of 30 mammals (including 27 primates), based on MultiZ alignments provided by the UCSC Genome Browser. Conservation was assessed using two methods from the PHAST package (phastCons and phyloP) both of which treat alignment gaps and unaligned nucleotides as missing data. The analyzed list of species and the phylogenetic three of the species are provided in Additional file 7.

### Patients and clinical data

During the patient recruitment, PE was defined as new-onset maternal hypertension after the 20^th^ week of pregnancy with proteinuria. PE was defined as women with the new onset of hypertension (systolic blood pressure of at least 140 mmHg or more and/or diastolic blood pressure of at least 90 mmHg) after 20-week gestation, accompanied by proteinuria (≥ 0.3 g protein in a 24-h urine specimen) [67]. Pregnant women with multiple gestations, smoking history, or chronic diseases (e.g., diabetes, thyroid disease, kidney dysfunction, autologous immune diseases) were excluded from the current study. Early-onset (EO)-PE is defined as delivery < 34 gestational weeks. Late-onset (LO)-PE is defined as delivery ≥ 34 gestational weeks. The following cohorts were used in the study: The Charité-Berlin (EO-PE *n* = 10; Healthy *n* = 8), Oslo (EO-PE *n* = 24; Healthy *n* = 27) and Essen (EO-PE *n* = 10; Healthy *n* = 7) cohorts contain placental samples from EO-PE and normotensive, healthy pregnancies. The Charité-Aachen cohort consists of preeclamptic (both EO-PE and LO-PE) and IUGR term placenta samples and gestational age-matched controls. The Manchester Antenatal Vascular Service cohort includes maternal plasma samples, collected from 24 to 28^th^ weeks of gestation from a high-risk cohort in the UK. The Graz-Aachen cohort consists of 141 healthy placental samples (*n* = 92 samples from early gestation and *n* = 49 samples at term). The Non-pregnant Female cohort consists of plasma samples from 6 non-pregnant healthy females. For the detailed characteristics of the cohorts, see Additional file 12 and references [31, 68, 69].

### Immunohistochemistry (IHC) staining of EPS8L1 in human placenta tissue

EPS8L1 expression was analyzed from first trimester and term placenta. Paraffin-embedded tissues (*n* = 7–8 per group) were cut into 4-μm sections, dewaxed, rehydrated, and subjected to a heat-induced epitope retrieval (HIER) method in 0.01 M citrate buffer (pH = 6). Endogenous enzyme activity and non-specific binding were blocked by hydrogen peroxide and 5% goat normal serum, respectively. Primary antibody (rabbit polyclonal anti-EPS8L1; CPA5398, Cohesion BioSci.) was diluted (1:100) in diluent (1% BSA + 2% goat normal serum in TBS) overnight at 4 °C. Antibody diluent without the primary antibody was used to identify non-specific staining. Subsequently, slides were washed in TBS and incubated with HRP-conjugated goat-anti-rabbit secondary antibody (111–035–047, Jackson ImmunoResearch) for 1 h at RT. The signal was detected in diaminobenzidine (DAB), followed by counterstaining of nuclei with 0.1% Mayer’s hematoxylin. The stained slides were dehydrated in ascending alcohols and cleared in xylene, and finally mounted with Entellan (Merck).

### EPS8L1 ELISA on human serum samples

Secreted EPS8L1 levels were analyzed in the maternal serum of control (*n *= 12) and PE women (*n *= 12) from the Manchester Cohort. Additional control serum samples from non-pregnant females (*n* = 6) were included. EPS8L1 ELISA was performed according to instructions (MyBioSource, MBS9317820, USA). Each sample was tested in duplicates and the mean of two measurements were used for statistical analysis. Data are presented as mean ± SEM and were analyzed with Shapiro–Wilk normality test followed by ordinary one-way ANOVA and Tukey’s multiple comparisons test. A *P*-value less than 0.05 was considered as significant.

### Primers

For the primer sequences used in the study—see Additional file 13.

### EPS8L1 overdosed in vitro PE model

SGHPL-4 trophoblasts stably overexpressing EPS8L1 were generated using *Sleeping Beauty* (SB100X) [61]. Human EPS8L1 coding sequence (ID: refNM_133180.2) was PCR amplified from placental cDNA and cloned into pT2B-puro construct. SGHPL-4 cells stably expressing EPS8L1 were selected via puromycin selection (3 μg/ml) for 15 days. Increased expression of EPS8L1 was confirmed at the mRNA and protein levels using qPCR and Western blotting, respectively. The expression of EPS8L1 was visualized by immunofluorescence staining.

### Invasion assay

Transwell invasion assay was performed to study the overdosing effect of EPS8L1 on the invasion of SGHPL-4 cells. Pre-chilled transwell inserts were coated with 50 μl of growth factor reduced matrigel (0. 25 mg/ml) diluted in Hams F10 w/o FBS. Serum starved wild-type and OE-EPS8L1 SGHPL-4 cells were plated onto the matrigel-coated wells. EGF (20 ng/ml) was used as a positive control and 0% FBS was used as a negative control. After 12–14 h of incubation, invaded cells were fixed and stained. Cells were fixed with 4% PFA and permeabilized by 100% methanol. Both invaded and non-invaded cells were then stained with 0.2% crystal violet. The non-invaded cells were scraped off by using cotton swabs. The images were taken under a light microscope (bright field) and the invaded cells were counted using the ImageJ software.

### Tube formation assay

Tube formation ability of OE-EPS8L1 SGHPL-4 cells was assessed by this assay. The µ-slide angiogenesis (Ibitreat) were used to perform the assay. The wells inside the slide were coated with growth factor-reduced matrigel. Wild-type and OE-EPS8L1 SGHPL-4 cells were coated on to the matrigel and were incubated for 6 h. To stimulate angiogenesis, EGF was used as a positive control and 0% FBS was used as a negative control. Images were taken after 6 h and were analyzed by using the Wimasis Image Analysis software. Different tube formation characteristics such as total number of tubes, tube length, loops, and branching points were analyzed and quantified.

### DCFH-DA assay

To measure the total reactive oxygen species (ROS), a total of 5 × 10^4^ OE-EPS8L1_SGHPL-4 cells and WT-SGHPL-4 cells (control) per well were seeded on a 24-well plate and incubated for 24 h in 37 °C, 5% CO_2_, and treated with 0.05 mM DCFH-DA for 45 min. DCFH-DA has the ability to diffuse rapidly into the cells and becomes deacetylated by cellular esterases and form DCFH (2’, 7’-Dichlorodihydrofluorescin) which is not fluorescent. In the presence of reactive oxygen species, DCFH is oxidized further to form green fluorescent (GF) DCF. The GF signal was analyzed on FACS calibur system using Cell Quest Software.

### Detection of EPS8L1 expression in the placental tissue

*Western blot analysis* was first performed on human placental samples of the Oslo Cohort (healthy (*n* = 4) and early-onset-PE samples (*n* = 4). Total protein was extracted from healthy and PE placenta tissue lysates. Around 40 µg protein lysate was loaded for each sample. The primary antibodies were rabbit anti-EPS8L1 (1:250, HPA041851, Atlas Antibodies) and mouse anti-ß-actin (1:1000, Dianova). The secondary antibodies were goat anti-rabbit (1:5000, Thermo Scientific) and goat anti-mouse (1:5000, Thermo Scientific) conjugated with horseradish peroxidase. Membranes were developed using the Amersham ECL prime detection reagent (GE Healthcare). Pre-stained protein ladder (26,616; Thermo Scientific) was used. The quantification data show the relative amounts as ratio of each isoform band relative to loading control (Actin). ImageJ software was used to quantify the protein bands of EPS8L1 isoforms. Data are presented as mean ± SEM and were analyzed with Shapiro–Wilk normality test followed by unpaired *t*-test. A *P*-value less than 0.05 was considered as significant.

### Detection of EPS8L1 expression in the maternal plasma

Different EPS8L1 isoforms were also detected using western blotting in the maternal plasma (healthy pregnant) as compared to the placenta tissue and BeWo cell lysates.

*Western blot analysis* was first performed on human plasma samples of the Oslo Cohort (healthy (*n* = 4) and early-onset-PE samples (*n* = 4). Plasma samples were diluted (1:10) in water before loading. Western blot confirmed the secreted isoforms of EPS8L1.

### Detection of PIGF and sFLT1 levels in the maternal plasma

The levels of serum PIGF and sFLT1 of patients were measured on Elecsys (Roche Diagnostics) by the HELIOS Klinikum GmbH.

### Isolation of primary human trophoblast cells and RNA sequencing

The human placenta tissue samples from healthy (*n* = 8) and preeclamptic women (*n* = 10) were collected at HELIOS Klinikum in Buch, Berlin. The Regional Committee of the Medical Faculty of Charité Berlin approved the sampling procedure: (EA4/069/15) (IRB of the Charité – Universitätsmedizin Berlin). The placenta tissue was processed within 2 h of delivery and primary trophoblasts were isolated according to the methods originally described in [70]. The detailed RNA isolation protocol and RNA sequencing analysis of human trophoblast cells has been described in ref [31].

### GFP-based enhancer reporter constructs and assay

In order to test the enhancer activity of the retroviral LTRs, GFP-based enhancer reporter constructs were generated by using Gibson assembly protocol (New England Biolabs). The predicted retroviral-LTR enhancer sequences of PE dysregulated candidate genes were downloaded from Repeat Masker. The retroviral LTR sequences were amplified from genomic DNA of human placenta tissue. The pT2-GFP reporter construct was used to clone the retroviral-LTR upstream of the CAG promoter. A construct without an LTR sequence was used as a negative control for the assay. For the assay, wild-type BeWo cells were cultured for 24 h in a 12-well plate. Retroviral LTR reporter constructs were transfected using lipofectamine according to the manufacturer’s protocol. As an internal control, mCherry construct having the same CAG promoter was transfected along with the retroviral LTR constructs. After 48 h of transfection, both GFP and mCherry signals were measured by flow cytometry for each LTR construct. FlowJo_V-10_CL software was used to analyze the FACS data and the cell population co-expressing GFP and mCherry was further analyzed. The median florescence intensity (MFI) was calculated for the GFP signal and was normalized to the MFI of the mCherry signal of each sample. Data analysis was performed with GraphPad Prism 9.0 (GraphPad Software, Inc.). Data are presented as mean ± SEM and were analyzed with Shapiro–Wilk normality test followed by unpaired two-tailed Student’s *t* test. *P ≤ 0.05 was considered as significant. Data is presented from four independent experiments.

### EPS8L1 mass spectrometry (MS) analysis

To identify the protein interaction partners of EPS8L1 in trophoblast, immunoprecipitation (IP) of HA-tagged EPS8L1 was used. HA-tagged EPS8L1 was transiently overexpressed in SGHPL-4 and wild-type BeWo cells. As a control, EPS8L1 construct without an HA-tag was used. The transfection was performed by Lipofectamine 3000 (Invitrogen) transfection reagent according to the manufacturer’s instructions. Three independent transfections were performed. The cells were collected, washed, and lysed in TRIS-lysis buffer after 48 h of transfection. The cell lysate was subjected to immunoprecipitation using anti-HA Agarose beads (Sigma-Aldrich EZview Red Anti-HA Affinity Gel) according to the manufacturer’s protocol. The protein pellet was analyzed by mass spectrometry. The list of potential interacting partners of EPS8L1 was generated (− log10 *P*-value > 1.25; *P*-value cut-off 0.056) that was further filtered by removing proteins that were significantly less abundant in the pull down than in the control (Additional file 9). Data was analyzed by ShinyGO v0.61 software: Gene Ontology Enrichment Analysis to characterize the biological and molecular function and also the enriched pathways of EPS8L1 interactors.

### Knock-out (KO) of EPS8L1 in trophoblast cells

EPS8L1 was knocked out (KO) in wild-type SGHPL-4 and wild-type BeWo cells using the CRISPR-Cas9 system [71] (Additional file 5). Three different guide RNAs (designed by the CRISPOR design tool) were used to target EPS8L1 (exon 2). The selected guide RNAs were the top three with low predicted CFD off-target scores, which are unlikely to be cleaved [72]. The guide RNAs containing constructs were transfected into BeWo or SGHPL-4 cells, along with a control plasmid without insert. After 48 h of transfection, GFP-positive cells were sorted by flow cytometry and cultured for 5*–*7 days. Western blot analysis of the total protein isolated from the cells was performed to confirm the KO of EPS8L1. After sorting, the SGHPL-4 cells were unable to grow as an individual clone and could not survive at all, in contrast to the BeWo cells, which could survive 5–7 days of in vitro culture but eventually died. Note that we could not perform further experiments with EPS8L1 KO cells as they could not survive in in vitro culture. The KO-EPS8L1 experiment was repeated at least three times with technical replicates.

### In vitro cell culture

BeWo is a human placenta choriocarcinoma cell line and SGHPL-4 cells are human extravillous trophoblast cells derived from first trimester. BeWo cells were maintained in DMEM/F12-GlutaMAX supplemented with 10% (v/v) FCS and 1% Antibiotic–Antimycotic (AA). SGHPL-4 cell were cultured in Ham’s F10 medium supplemented with 10% (v/v) FCS and 1% AA. The cells were cultured at 37 °C in 5% CO_2_.

### DNA methylation and HDAC treatments

BeWo cells were grown for 24 h, then fed with medium containing 50 µM 5-azacytidine (Sigma) for 72 h and 1 μM Trichostatin A (TSA) for 4, 8, 18, and 24 h respectively, before harvesting the total RNA. The expression of the 6 candidate genes EPS8L1, CYP11A1, CSF2RB, ALDH3B2, DACT2, and SPINT1 was quantified in the treated versus non-treated cells by qPCR analysis. The expression of DACT2 after the treatment with TSA was at the undetectable level. The data were collected from 4 independent experiments. Data analysis was performed with GraphPad Prism 9.0 (GraphPad Software, Inc.). Data are presented as mean ± SEM and were analyzed with Shapiro–Wilk normality test followed by unpaired two-tailed Student’s *t* test or ordinary one-way ANOVA followed by Dunnett’s multiple comparisons test or Kruskal–Wallis test followed by Dunn’s multiple comparisons test was. *P ≤ 0.05 was considered as significant.

### Overexpression of EPS8L1 by using the Sleeping Beauty (SB100X)-transposon system

SGHPL-4 trophoblasts stably overexpressing EPS8L1 was generated using the Sleeping Beauty (SB100X) system [73, 74]. Human EPS8L1 coding sequence (Sequence ID: refNM_133180.2) was amplified from placental cDNA and cloned into pT2B-puro *Sleeping Beauty* expression construct by using the specific primers. Cells were electroporated with 10:1 ratio of vector carrying EPS8L1 overexpression cassette (1 μg DNA) and plasmid pcGlobin2-SB100X containing hyperactive *Sleeping Beauty* transposase (SB100X; 100 ng DNA) with Neon Transfection System (Life Technologies) using parameters as follows: 1260 V/20 ms/2 as pulse voltage/pulse width/pulse number. SGHPL-4 cells stably expressing EPS8L1 were selected via puromycin selection (3 μg/ml) for around 15 days. Increased expression of EPS8L1 was confirmed at the mRNA and protein levels using qPCR and Western blotting, respectively. The expression and cellular localization of EPS8L1 protein in SGHPL-4 were carried out by immunofluorescence staining.

### Immunofluorescence (IF) staining of EPS8L1 in OE-SGHPL-4 cells

IF staining was used to confirm the overexpression of EPS8L1 in OE-EPS8L1 SGHPL-4 cells*.* To this end, an equal number of wild-type SGHPL-4 and OE-EPS8L1 SGHPL-4 cells were seeded on coverslips in 12-well cell culture plates and incubated overnight at 37 °C and 5% CO_2_. Cells were fixed by using 4% paraformaldehyde (Sigma) supplemented with Hoechst 33, 342 (1: 1250; Invitrogen) and permeabilized with 0.1% Triton X-100. After blocking (5% BSA for an hour), cells were incubated with primary antibodies for Actin and EPS8L1 (rabbit anti-EPS8L1 antibody, HPA041851, 1:100) and stored overnight at 4 °C. After washing, the cells were incubated with secondary antibodies Alexa Fluor 488 anti-rabbit (goat) and Alexa Fluor 647 anti-mouse (donkey) 1:200 for 1 h. Nuclei were stained with DAPI solution (Vectashield with DAPI) and coverslips were mounted using ProLong® Gold Antifade Reagent (Invitrogen). Images were taken using a Leica LSM710 point-scanning single photon confocal microscope [75].

### Immunohistochemistry (IHC) of EPS8L1 in human placenta tissue

Analysis of the IHC stained samples: Images were taken using a Leica LSM710 point-scanning single-photon confocal microscope [75]. Stained slides were digitally scanned by a high-resolution brightfield slide scanner (Pannoramic MIDI BF/FL, 3DHISTECH Ltd.), and staining intensity was scored by a subjective scale (0, negative; 1, weak; 2, moderate; 3, strong) on virtual scans using CaseViewer (3DHISTECH Ltd.) at × 100 magnification by two independent examiners blinded to group allocation. The immunohistochemical results were evaluated by a semiquantitative approach using the H-score. Data are presented as mean ± SEM and were analyzed with Shapiro–Wilk normality test followed by unpaired two-tailed Student’s *t*-test or the Mann–Whitney *U* test. A *p* value less than 0.05 was considered as significant.

### Transcriptome analysis of EPS8L1 overexpressing SGHPL-4 cells (OE-EPS8L1 SGHPL-4 cells)

Total RNA was isolated from wild-type and OE-EPS8L1 SGHPL-4 cells using RNeasy mini kit (Qiagen). Three independent transfections as biological replicates were used for the study. All the samples passed the quality check and were processed for sequencing (BGI, Honkong, China). The libraries were prepared according to protocol of BGISEQ-500 platform. The methodology to calculate the differentially expressed genes from the fastq files was similar to the pipeline described in the data analysis of RNA isolated from human primary trophoblasts. The transcriptome analysis was performed by BGI and [45].

### Bisulphite treatment and sequencing

To assess the CpG methylation sites in healthy and PE LTR sequences, Methylation Assay was performed. Genomic DNA was extracted from frozen placenta tissue from three healthy controls and three EO-PE patients by using the DNeasy Blood and Tissue Kit (Qiagen, cat. 69,504). The genomic DNA from both healthy and EO-PE patients were treated with sodium bisulphite and the methylation-specific PCR was designed according to the manufacturer’s instructions (EpiTect Bisulfite Kit, Qiagen, Cat. No./ID: 59,104). Sodium bisulphite converts all the cytosine residues to uracil and leaves 5-methylcytosine residues unaffected. The primers for the bisulphite sequencing PCR were designed using the MethPrimer software. Sodium bisulphite-treated genomic DNA was used as a template for the methylation-specific PCR. The EpiTect MSP Kit (Qiagen, Cat. No./ID: 59,303) was used to setup the methylation-specific PCR. We designed methylation-specific PCR primers for all LTR sequences of the candidate genes. However, due to the highly repetitive nature of the LTR sequences, we were only able to successfully amplify the CpG site on MLT1F2-2 upstream of CYP11A1 from the bisulfite-treated genomic DNA, which had only a single CpG site ([76]. See also Additional files 8 and 13.

### Trajectory and diffusion analysis

Trajectory analysis of the differentiation process from progenitors to committed cells was performed using the Monocle2 package [77], which generates a pseudotime plot that graphically illustrates the branched and linear differentiation processes. For pseudo-temporal analysis, the data was first imported the processed Seurat object into the Monocle2 workspace using “importCDS” function. The datasets were processed further using the series of default functions with negative binomial expression family parameters. The final dimensionality was reduced to two components. The dimensionality of the data was reduced by constructing a parsimonious tree using “DDRTree.” We employed the “differentialGeneTEst” function to find the top DEGs (*q*-value < 1e^−8^), these being fed as an input for unsupervised ordering of the combined set of cells. Note: the *q*-value threshold was set manually (10^−8^–10^−15^) to find the root, branching point, and leaves on the trajectory graphs. This approach identified the genes (100 to 1000) that were significantly differentially expressed. We then used the expression data of these genes to construct a diffusion map for the respective cell populations (DiffusionMap function in the Destiny package [78]). We calculated the diffusion pseudo-time (DPT function in the Destiny package). Finally, the cells on the diffusion maps and trajectories were annotated on the basis of their identity, previously determined from the Seurat analysis.

### Single-cell RNA-seq (scRNA-seq) data analysis

We reanalyzed single-cell RNA sequencing data generated on SMART-seq2 platform from human placenta ([79] (GSE89497). Reads were mapped to the human genome (hg19) using STAR (https://github.com/alexdobin/STAR) with defined settings, i.e., –align Intron Min 20 align Intron Max 1,000,000 –chim Segment Min 15 –chim Junction Overhang Min 15 –out Filter Multimap Nmax 20, and only uniquely mapped reads were considered for the calculation of expression. We obtained counts using feature Counts (http://subread.sourceforge.net/) at gene level with Ref Seq annotations. Gene expression levels were calculated at Transcript Per Million (TPM) from counts over the entire gene (defined as any transcript locating between TSS and TES). This we did using our in-house R script (available on request). We only chose cells that expressed at least 5000 genes and genes that were expressed in at least 1% of cells (log2 TPM > 1 threshold was regarded as expressed). We clustered the cells using the default parameters of “Seurat” version 2.3 (http://satijalab.org/seurat/) package from R. This applies the most variable genes to get top 10 principal components, and the most discriminating genes cluster the cells on tSNE or UMAP. We identified the major clusters corresponding to CTB, STB, EVTB, Macrophages, and stromal cells. We defined the cell type for each cluster according to the known markers.

### ChIP-seq and CUT&Tag data analyses

We obtained ChIP-seq datasets in raw FASTQ format for various trophoblast cell states and transcription factors (TFs): H3K27Ac in CTB and STB primary cultures ([55] GSE127288), H3K4Me1 in trophoblasts ([23], GSE118289), H3K4Me3 and H3K27Me3 in differentiated trophoblasts ([44], GSE105258), h3K4Me1 and H3K27Ac in human term placenta ([19], GSE118289), and GATA2/3 and TFAP2A/C ([52], (GSE105081)). ChIP-seq reads were aligned to the hg19 human reference genome using Bowtie2 (http://bowtie-bio.sourceforge.net/bowtie2/manual.shtml) with the –very-sensitive-local mode. Unmapped reads, reads with MAPQ < 10, and PCR duplicates were removed using Picard and Samtools (http://www.htslib.org/). Peak calling was performed with MACS2 (https://github.com/taoliu/MACS), using narrow mode for TFs and broad mode for histone modifications, with a false discovery rate (FDR) threshold of < 1%. ENCODE blacklisted regions (https://www.encodeproject.org/annotations/ENCSR636HFF) were excluded from the peak sets. Peaks were intersected with repeat elements annotated in the hg19 RepeatMasker track using Bedtools (https://bedtools.readthedocs.io/en/latest/) *intersectBed* with a minimum of 50% reciprocal overlap. For visualization in IGV, raw ChIP-seq signal tracks were generated using MACS2 with the following parameters: -g hs -q 0.01 -B. RefSeq genes (hg19) were used as genomic context. Conservation across non-human primates (NHPs) was visualized using the UCSC Genome Browser’s net/chain alignment tracks and integrated below the IGV signal tracks.

### Identification of potential AP-1 binding in MLT1 LTR genomic consensus

The position weight matrix (PWM) for the FOS::JUN (AP-1) transcription factor was obtained from the JASPAR database. The PWM was provided in raw count format and was first normalized to position-specific probabilities. To avoid zero probabilities, a pseudocount of 1 was added to each count prior to normalization. Normalization was performed independently for each position by dividing the adjusted counts by the column sum.

The scoring function (calculate_pwm_score) applied a logarithmic transformation to the normalized probabilities (np.log(pwm[nucleotide][i] + ε)) to emphasize nucleotides with high motif preference and to penalize mismatches more strongly. Here, ε denotes a small constant used to prevent undefined log values.

The DNA sequence of interest was retrieved from a FASTA file using a custom parser (read_sequence_from_fasta). A sliding-window approach was then employed to scan the sequence with a window size of 7 nucleotides, corresponding to the length of the FOS::JUN motif. For each window, a PWM-based log-likelihood score was computed, and subsequences with normalized scores above a threshold of 0.5 were designated as potential AP-1 binding sites.

#### Normalization and log scores

The provided PWM was assumed to be in count format. To normalize the PWM and address potential zero probabilities, we added 1 (pseudocount) to each count before calculating the position-specific probabilities. The scoring function (calculate_pwm_score) employed log transformation on the normalized probabilities (np.log(pwm[nucleotide][i] + 1)) to convert the linear relationship between probabilities and scores into a logarithmic one. This emphasizes the contribution of nucleotides with higher probabilities within the motif and penalizes mismatches more severely. (1) PWM Data: The normalized PWM for FOS::JUN was obtained from JASPAR. The DNA sequence of interest was retrieved from the FASTA file using the custom function read_sequence_from_fasta; (2) Sequence scanning and scoring: A sliding window approach was employed to scan the sequence for potential binding sites. The window size was set to 7, corresponding to the length of the FOS::JUN motif. For each window, a score was calculated using the calculate_pwm_score function, which combines the normalized PWM probabilities and log transformation. A threshold of 0.5 was applied to identify subsequences with high similarity to the motif.

### Intersection of PE patients and OE-EPS8L1 SGHPL-4 cell datasets

The transcriptome of overexpressing (OE)-EPS8L1 cells and PE patients were subjected to comparative analysis. Two PE microarrays data sets described in ref [31] were used for this analysis. A matrix of expression level for unique genes in each sample was generated and the two datasets from different platforms were merged by their unique gene name in total for 24 PE, 22 control samples. The batch effect arising from two different platforms was corrected by normalizing surrogate variances from “Combat” package from R Bioconductor. The corrected batch effect was confirmed by principal component analysis (PCA). Each gene expression value was further assigned as their relative abundance value, which is the ratio of expression level of a gene in each sample and mean expression values of the gene across the samples. Lastly, EPS8L1 gene from the obtained relative abundance matrix was fetched. Notably, EPS8L1 over expression is significantly represented in PE cohorts compared with controls (relatively higher in the ~ 60% of PE patients and ~ 25% of controls, hypergeometric *P*-value < 0.03). Correlation analysis (Pairwise Spearman Rank) was performed to identify the commonly dysregulated genes between the DEGs in PE vs controls, and EPS8L1-OE vs controls (DEGs obtained by setting the criterion of adjusted *P*-value < 0.05) on log2 fold change. Genes that were commonly differentially expressed (*n* = 230) between the two datasets are shown in the scatterplot.

### Cell type analysis of the nine PE candidate gene expression in human and mice

For the analysis mouse single-cell transcriptome data from the https://bis.zju.edu.cn/MCA and the human protein atlas https://www.proteinatlas.org/ were used. The expression data were sorted into the following cell types: *Glandular epithelial cells*, *Squamous epithelial cells*, *Specialized epithelial cells*, *Endocrine cells*, *Neuronal cells*, *Glial cells*, *Germ cells*, *Trophoblast cells, Endothelial cells*, *Muscle cells*, *Adipocytes*, *Pigment cells*, *Mesenchymal cells*, *Undifferentiated cells*, *Blood and immune cells*. The mice data were handled similarly. Note that the trophoblast lineages are *Cytotrophoblast (CTB)*, *Syntiotrophoblast (STB)*, *Extravillous trophoblast (EVTB)* and *Trophoblast giant cells* (TGC), *Spongiotrophoblast* (STP), in human and mouse respectively (see also Additional file 6).

### Statistical analyses

All the experimental were analyzed by using GraphPad Prism 9.0 (GraphPad Software, Inc.). Data are presented as either mean ± SEM or +/− SD depending on context (for normally distributed data, assessed by Shapiro–Wilk normality test) or median with inter-quartile range (for non-normally distributed data). Unpaired *t*-test, nonparametric Mann–Whitney test, ANOVA, or Kruskal–Wallis test were employed as appropriate with post hoc Bonferroni or Dunn’s tests. Correlation between nonparametric variables was analyzed using Spearman’s rank correlation. Two-tailed testing with normal-based 95% confidence interval was performed for each analysis, and *P*-value < 0.05 was considered statistically significant. Further details can be found in the figure legends.

## Supplementary Information

Additional file 1: Identification of ERV-derived enhancers in the trophoblast.

Additional file 2: Table S1. List ofERV-derived“strong”, “weak”, or “non-enhancers”.

Additional file 3: Table S2. List of 87 ERV enhancer-gene pairs.

Additional file 4: Supplementary Figures.

Additional file 5: Knocking out MLT1G1 upstream EPS8L1.

Additional file 6: Cell type specificity in human and mouse.

Additional file 7: Evolutionary conservation analysis of MLT1/2.

Additional file 8: Bisulfite sequencing.

Additional file 9: Table S3. Interactome of EPS8and EPS8L1.

Additional file 10: Table S4. Protein encoding isoforms of EPS8L1.

Additional file 11: Table S5. List of differentially expressed genes in OE-EPS8L1_SGHPL-4 cells.

Additional file 12: Patient and clinical data.

Additional file 13: Primers used in the study.

Additional file 14: Availability of Data and Materials.

Additional file 15: Detailed author’s contributions.

## Data Availability

All data are available in the main text or the supplementary materials. New gene expression data generated for this paper is deposited into GEO under accession number: GSE263305 [45]. The bisulfite sequencing data of the MLT1F2-2 element upstream of CYP11A1 is available at [76]. ERV-derived enhancer prediction was deposited to https://github.com/amitpande74/human-transposons-enhancer-predicition and 10.5281/zenodo.16022218 [43, 44]. Full versions of gels, IF or IHC images are available at 10.6084/m9.figshare.29738765 [75]. Third party data utilized in the study are listed in Additional File 14.
