# Supplementary material for: ERV3-MLT1 provides cis-regulatory elements for human placental functioning and are commonly dysregulated in human-specific preeclampsia
Source: Genome Biol. 2025 Nov 5;26:364. doi: 10.1186/s13059-025-03821-1 (PMC12587658; doi:10.1186/s13059-025-03821-1)
Supplement: Supplementary file 1 — Additional file 1: Identification of ERV-derived enhancers in the trophoblast. [file 13059_2025_3821_MOESM1_ESM.pdf]

Identification of ERV-derived enhancers in the trophoblast

To identify endogenous retrovirus (ERV)-derived enhancers in the trophoblast, we employed a one-dimensional convolutional neural network (1D CNN). This architecture was selected for its effectiveness in analyzing sequential data such as DNA sequences, and for its ability to operate without explicit feature selection (an advantage given that our training data consisted of sequences already validated as enhancers) [1]. To further refine the model's discriminatory power, we included non-enhancer sequences during training, enabling the network to learn distinguishing features specific to enhancers.

The human genome harbors a complex landscape interspersed with sequences derived from ancient endogenized retroviruses, known as endogenous retroviral (ERV) elements. Some of these ERVs have been co-opted to function as enhancers, influencing host gene expression. In this study, we investigated whether all ERV subclasses have the potential to act as enhancers and, if so, sought to classify them according to their relative enhancer strength.

• Data Collection

We obtained experimentally validated enhancer sequences from publicly available databases [1–5]. To ensure that our model could accurately distinguish true enhancer features from background genomic noise, we incorporated randomly selected non-enhancer sequences as negative controls in the training dataset. These negative sequences were generated from the hg19 reference genome using the gkmSVM package [6], matched for length distribution and GC content to avoid confounding biases. As a result, the final dataset comprised three categories: experimentally validated enhancers, shadow enhancers, and matched negative control sequences.

• Data Preparation

- o One-Hot Encoding: Each nucleotide sequence was transformed into a binary matrix representation using one-hot encoding. This process converts the nucleotide bases (A, C, G, and T) into a binary format, facilitating their use as inputs for the CNN.
- o Data Augmentation: To mitigate overfitting and enhance the model's ability to generalize, data augmentation was performed. This involved introducing random mutations at a rate of 0.01 into the sequences, thereby generating augmented data that simulates natural sequence variations.

• Model Architecture and Training

CNN Architecture: The model was constructed using the TensorFlow and Keras libraries. The architecture comprises the following.

1. ▪ Input Layer: Accepting one-hot encoded sequences.

2. ▪ Convolutional Layers (Conv1D) are utilized for feature extraction from sequences. The number of filters and the kernel size were subjected to hyperparameter tuning.

3. ▪ MaxPooling1D: The following convolutional layers were applied to reduce dimensionality.

4. ▪ Batch normalization: Employed to stabilize and accelerate training.

5. ▪ Dropout: Integrated to prevent overfitting, with rates determined through hyperparameter tuning.

6. ▪ flattening: Convert pooled feature maps into a single vector.

7. ▪ Dense Layers: For classification, culminate in a sigmoid activation function for binary classification.

- Hyperparameter Tuning: Conducted using Keras Tuner, optimizing parameters such as the number of filters, kernel size, dropout rate, and number of units in dense layers.

Training Parameters:

1. ▪ Loss Function: Binary cross-entropy suitable for binary classification tasks.

2. ▪ Optimizer: Adam, with a learning rate determined through hyperparameter tuning.

3. ▪ Batch Size: Set to 8, balancing computational efficiency and model performance.

4. ▪ Class Weights: Computed to address class imbalance in the training data.

5. ▪ Callbacks: EarlyStopping (patience of 10 epochs), ReduceLROnPlateau (reducing the learning rate by a factor of 0.2 after five epochs of no improvement), and ModelCheckpoint (saving the best model based on validation loss).

• Padding and Sequence Length: To handle sequences of varying lengths, padding was applied to standardize the length of all input sequences. The maximum sequence length was determined based on the longest sequence in the dataset, with shorter sequences padded at the end using a 'post' padding strategy.

• Model Evaluation
Cross-Validation: Stratified k-fold cross-validation (with k = 5) was employed to assess the model's performance. This approach ensures that each fold is representative of the overall dataset, providing a robust evaluation of the model's accuracy and generalizability.
Performance Metrics: Model performance was primarily evaluated in terms of accuracy. Additional metrics, such as precision, recall, and F1-score were also considered to provide a comprehensive assessment of the model's predictive capabilities.

Our model achieved a training accuracy of [98%] and a validation accuracy of [97%], indicating a robust ability to distinguish between enhancers and non-enhancers. Upon evaluating the HERV sequences (432 full-length), we found that [72%] were strong enhancers, [2.5%] were weak enhancers, and the remaining [25%] were non-enhancers.

Metric	Class 0.0	Class 1.0	Overall (Macro Avg)	Overall (Weighted Avg)
Precision	0.08	0.99	0.54	0.99
Recall	0.25	0.98	0.61	0.97
F1-Score	0.12	0.99	0.55	0.98
Support	28	3997	Nan	Nan

<u>Definitions of the metric columns of the table:</u>

1. Precision: Precision is the ratio of correctly predicted positive observations to the total predicted positive observations. For class 0.0, the precision is 0.08, meaning that only 8% of the instances predicted as class 0.0 are actually class 0.0 (enhancers). For class 1.0, the precision is very high (0.99), indicating that 99% of instances predicted as class 1.0 (non-enhancers) are actually class 1.0.

2. Recall: Recall (or sensitivity) is the ratio of correctly predicted positive observations to all observations in the actual class. For class 0.0, the recall is 0.25, meaning the model correctly identified 25% of all actual instances of class 0.0. For class 1.0, the recall is 0.98, indicating that the model correctly identified 98% of all actual instances of class 1.0.

3. F1-Score: The F1 Score is the weighted average of Precision and Recall. Therefore, this score takes both false positives and false negatives into account. F1-Score for class 0.0 is 0.12, and for class 1.0 its 0.99, indicating a very good performance for class 1.0.

4. Support: Support is the number of actual occurrences of the class in the specified dataset. For class 0.0, there are 28 instances, and for class 1.0, there are 3997 instances.

5. Accuracy: This is the ratio of correctly predicted observations to the total observations and is given as 0.97 or 97%.

6. Macro Average: The macro average calculates metrics independently for each class and then takes the average, treating all classes equally. The macro average for precision, recall, and F1-score are 0.54, 0.61, and 0.55, respectively.

7. Weighted Average: The weighted average takes into account the support of each class. This means the metrics are weighted by the number of instances in each class, making it more relevant for imbalanced datasets. The weighted averages for precision, recall, and F1-score are very high, mainly influenced by the high performance on the majority class (1.0).

The PRC curve (precision recall), ROC curve (receiver operating characteristic), Cumulative gains curve, Lift curve and confusion matrix analysis show the robustness of the model.

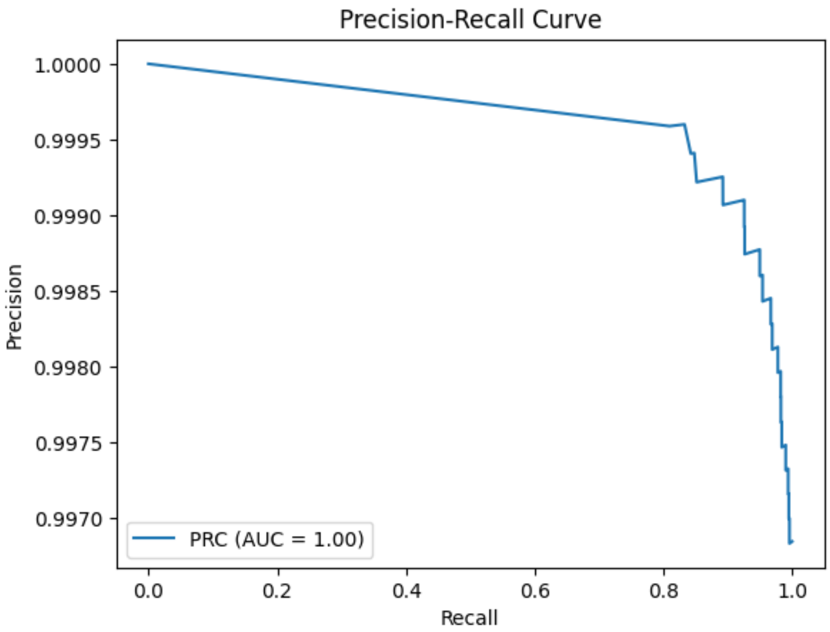

Precision-Recall Curve
Precision
Recall
PRC (AUC = 1.00)
1.0000
0.9995
0.9990
0.9985
0.9980
0.9975
0.9970
0.0
0.2
0.4
0.6
0.8
1.0

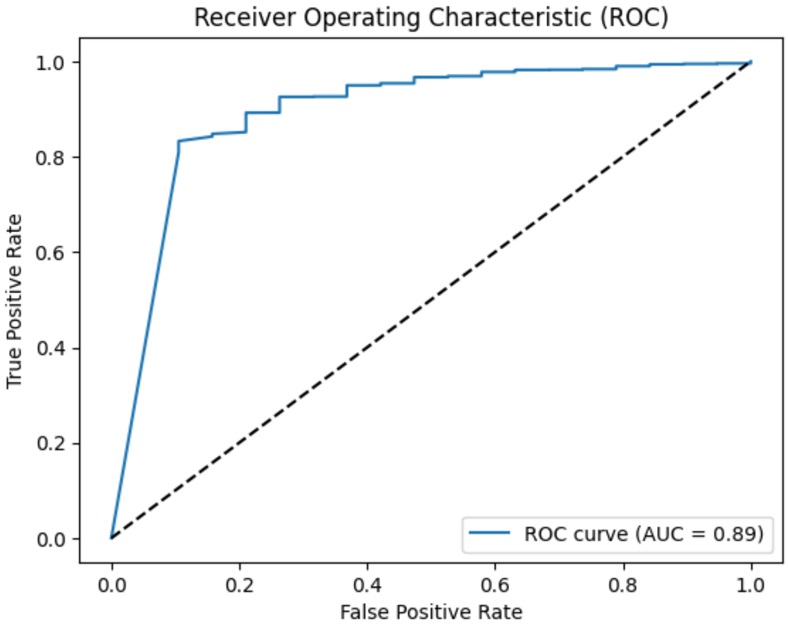

Receiver Operating Characteristic (ROC)
True Positive Rate
False Positive Rate
ROC curve (AUC = 0.89)
1.0
0.8
0.6
0.4
0.2
0.0
0.0
0.2
0.4
0.6
0.8
1.0

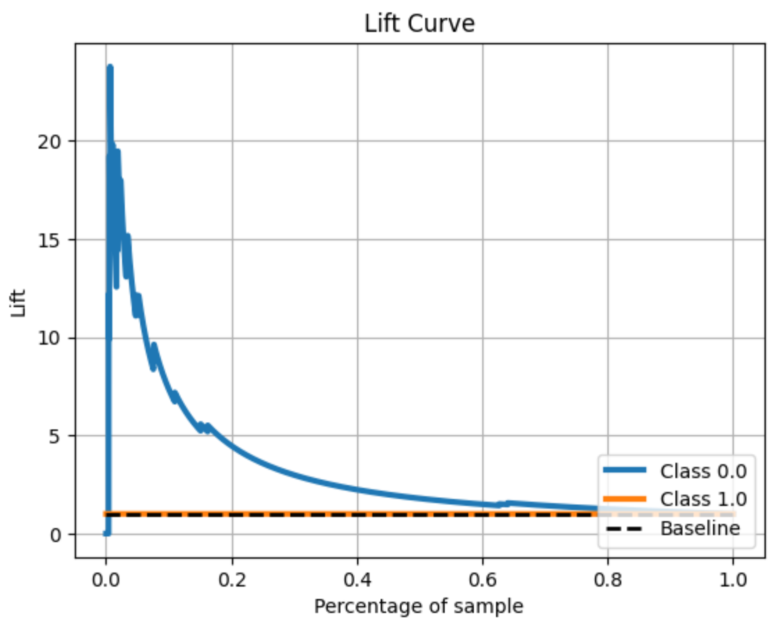

Lift Curve
Lift
0
5
10
15
20
0.0
0.2
0.4
0.6
0.8
1.0
Percentage of sample
Class 0.0
Class 1.0
Baseline

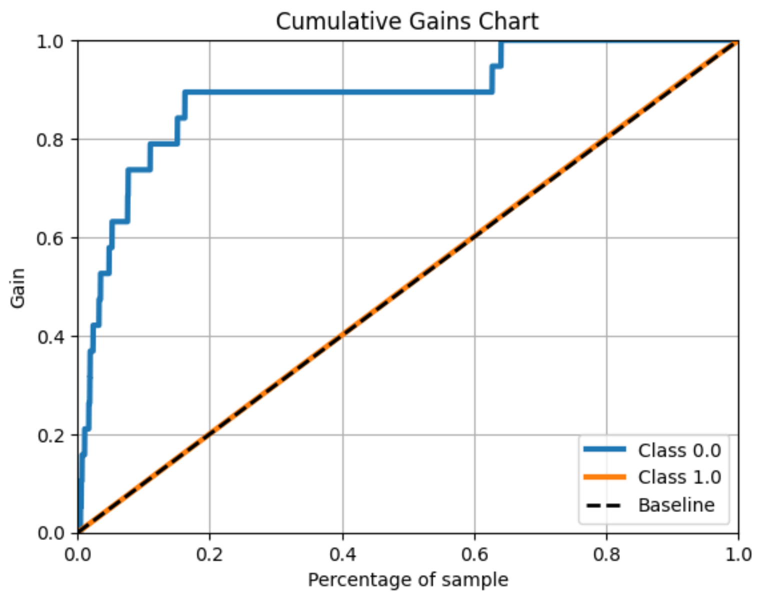

Cumulative Gains Chart
Gain
0.0
0.2
0.4
0.6
0.8
1.0
0.0
0.2
0.4
0.6
0.8
1.0
Percentage of sample
Class 0.0
Class 1.0
Baseline

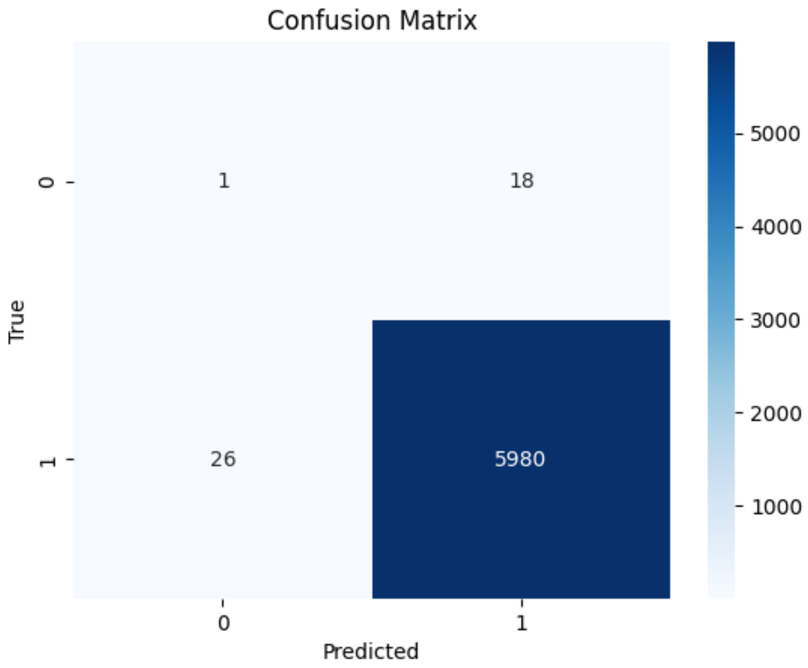

Strong Enhancers are GC rich

The sequence characteristics that differentiate enhancers from non-enhancers—and those that distinguish strong enhancers from weak ones—are clearly reflected in our dataset. A notable pattern is the high prevalence of guanine (G) and cytosine (C) bases in most enhancer sequences, in contrast to a marked depletion of adenine (A) and thymine (T) bases [1, 2]. This striking compositional difference highlights a fundamental biological distinction between enhancer and non-enhancer sequences, which contributes significantly to the performance of our classification model. In contrast, the differences between strong and weak enhancers are more nuanced, owing to overlapping biological features. As illustrated in the figure, strong enhancers are enriched for G and C bases, whereas weak enhancers tend to show the opposite pattern - being relatively A/T-rich and depleted of G/C content.

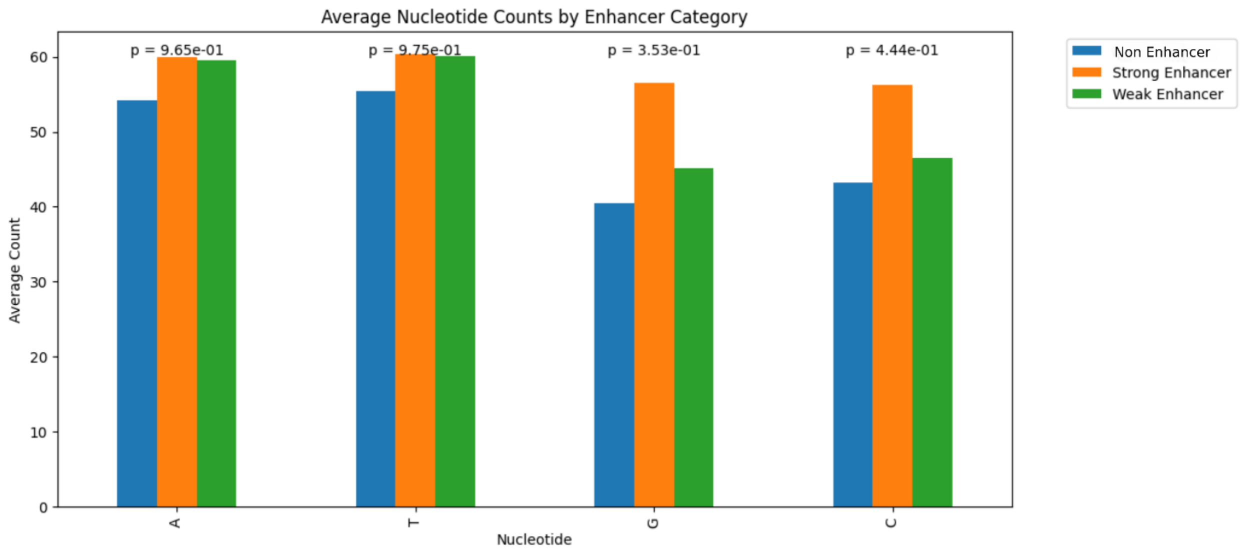

Our model offers comprehensive insights into the functional roles of ERV sequences, particularly highlighting their potential to act as enhancers. The substantial proportion of ERV-derived sequences exhibiting enhancer-like characteristics underscores their likely regulatory influence within the genome. Nonetheless, it is important to acknowledge potential biases in the initial training dataset, as well as the inherent limitations of modeling complex cellular environments in silico. Future investigations should focus on identifying the specific sequence motifs or structural features within ERVs that confer enhancer activity, thereby refining our understanding of their functional impact.

The code is available at Github:
https://github.com/amitpande74/human-transposons-enhancer-predicition
