# Supplementary material for: ERV3-MLT1 provides cis-regulatory elements for human placental functioning and are commonly dysregulated in human-specific preeclampsia
Source: Genome Biol. 2025 Nov 5;26:364. doi: 10.1186/s13059-025-03821-1 (PMC12587658; doi:10.1186/s13059-025-03821-1)

Fig. S1. Histone mark, P300 occupancy, and chromatin accessibility analyses of various ERV families in differentiated trophoblast cells (EVTBs differentiated from TSCs and trophoblast cells derived from H1_ESCs (Methods). Note that while many ERVs significantly enriched in the trophoblast H3K27Ac repertoire display both promoter-like (H3K4Me3 + H3K4Me1 + ATAC-seq) and enhancer-like (H3K4Me1 + ATAC-seq + P300) chromatin signatures, the MLT families are uniquely enriched in enhancer-associated (H3K4Me1 + ATAC-seq + P300) regions.

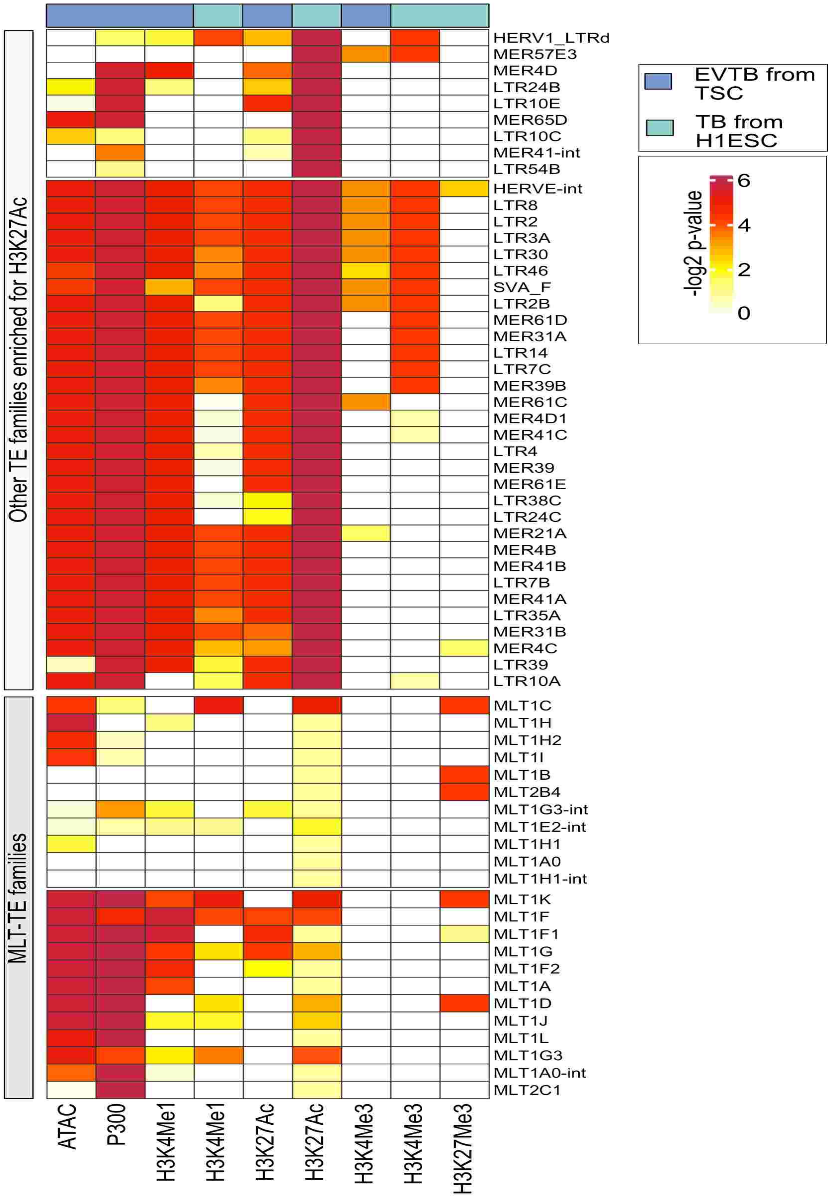

Fig. S2. Candidate genes (SLC22A11, PLEKHA8, NRK, RAD51, PHYHIPL, KIF23, C1QTNF6 and LY6E), whose significant dysregulation was not supported by qPCR in placental samples derived from pathological pregnancies (Oslo-cohort compared with healthy controls. EPS8 expression levels were analyzed in the same cohort (mean ± SEM; Control n = 27; Early-onset (EO)-PE n = 24; Late-onset (LO)-PE: n = 23) (**P ≤ 0.01; Kruskal–Wallis test; Dunn's multiple comparisons test.

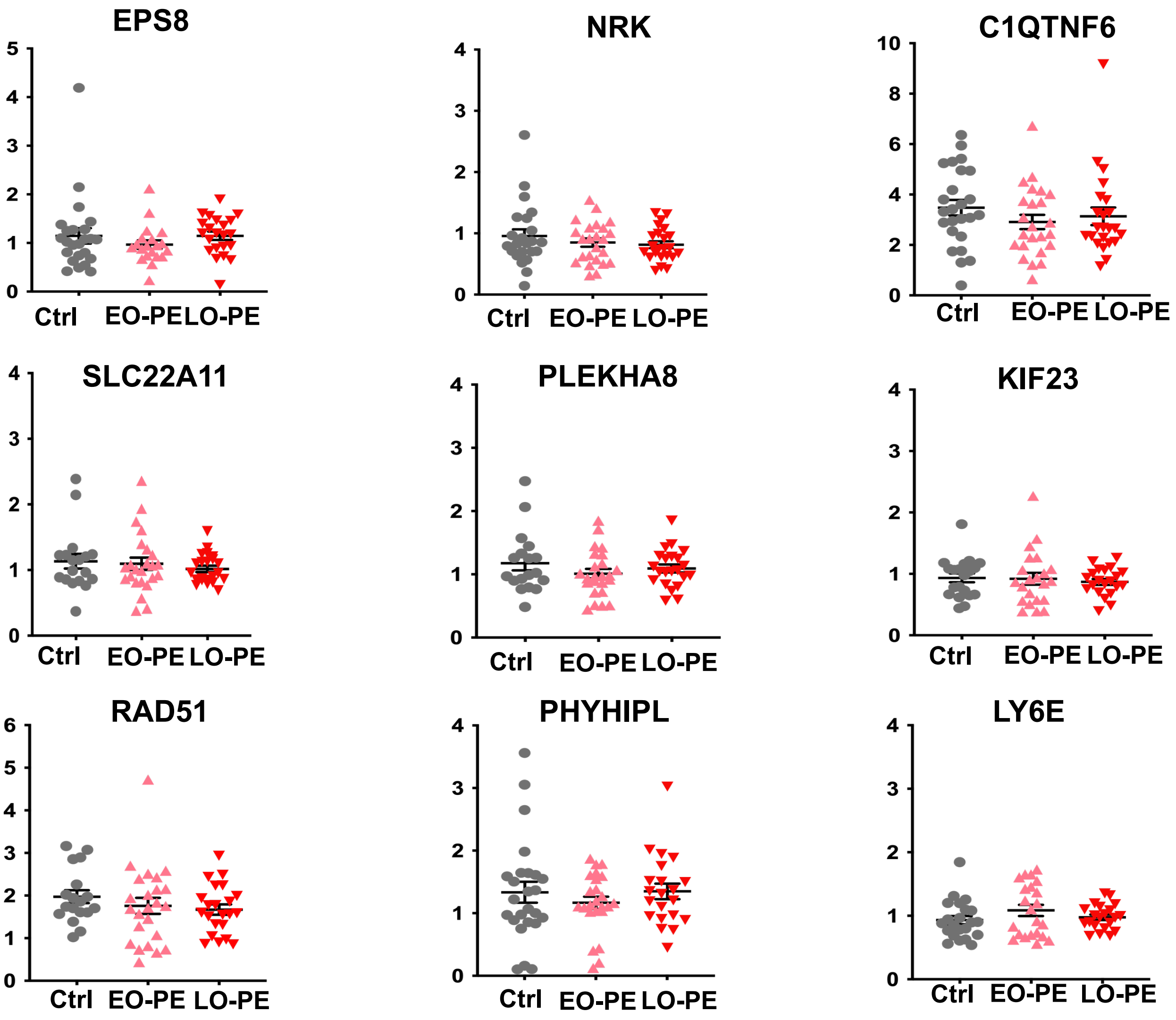

Fig. S3. (a) Mean CUT&Tag profiles for JUN and JUND and ChIP-seq profiles H3K4m1 and H3K27Ac histone modifications over MLT1 LTRs upstream of the six selected genes. (b) Determination of the functional enhancer potential of the ERV-LTR sequences identified as potential alternative regulatory sequences of the candidate genes. Schematic of GFP-reporter constructs to determine the functional enhancer potential of the ERV-LTR sequences, identified as potential alternative regulatory sequences of the candidate genes.

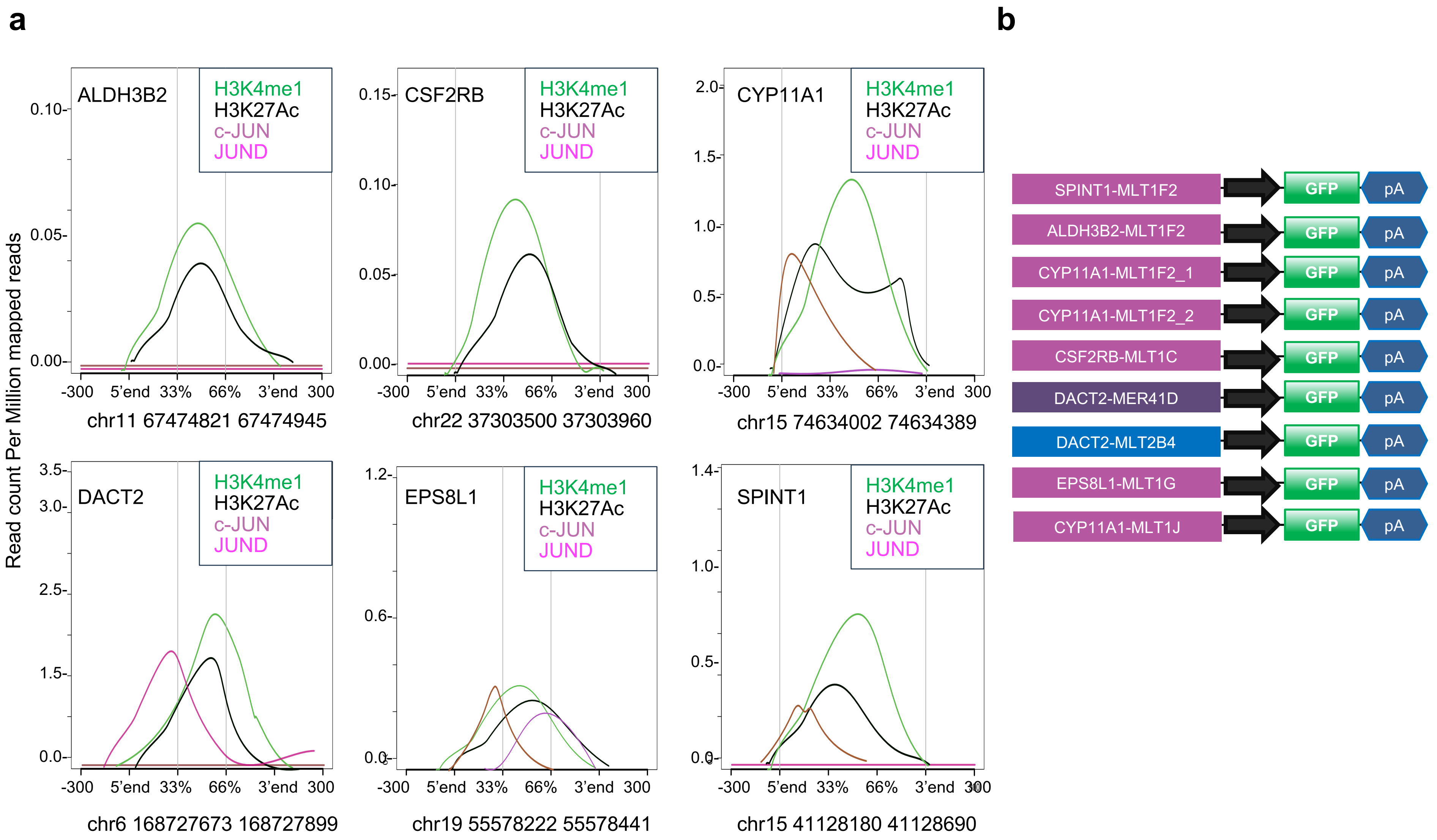

Fig. S4. Knocking out (KO) of MLT1G1 using CRISPR/Cas9 KO editing. (a) Schematic representation of sgRNAs targeting MLT1G1. (b) Genotyping analysis confirmed that sgRNA1 and sgRNA2 induce a ~200 bp deletion in the MLT1G1 locus. (c) Sanger sequencing analysis confirmed that sgRNA1 and sgRNA2 induced a ~200 bp deletion in the MLT1G1 locus.

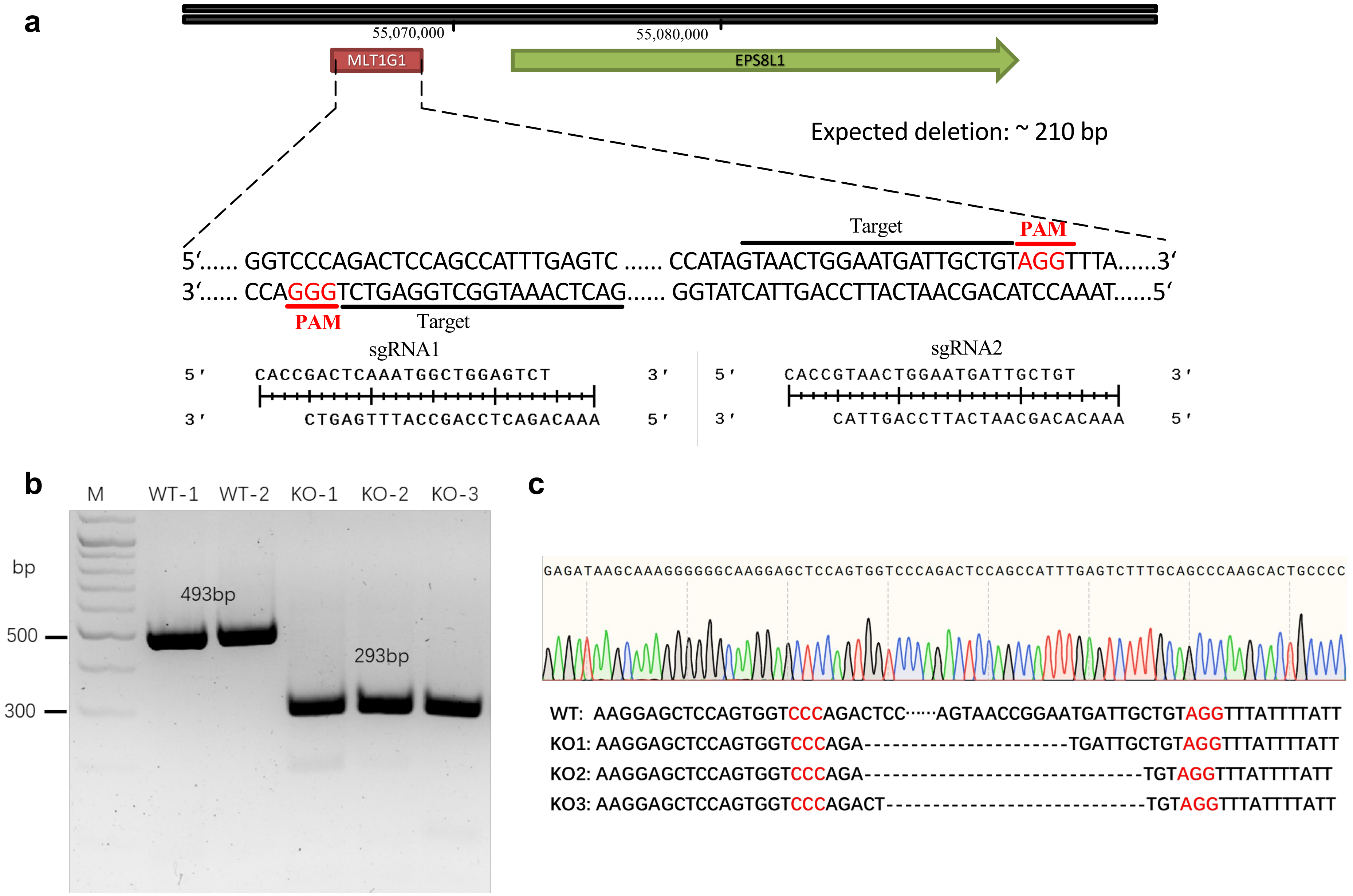

Fig. S5. CpG methylation analysis of the ERV3-MLT1(F2) enhancer from the *CYP11A1* locus. (a) Workflow of the CpG methylation analysis. (b) An example showing a variable CpG methylation at the ERV-MLT1(F2)_1 enhancer upstream of the *CYP11A1* locus. Sequencing analysis identified a demethylated CpG site in the tested EO-PE patients when compared to healthy control samples (n=3 vs. 3).

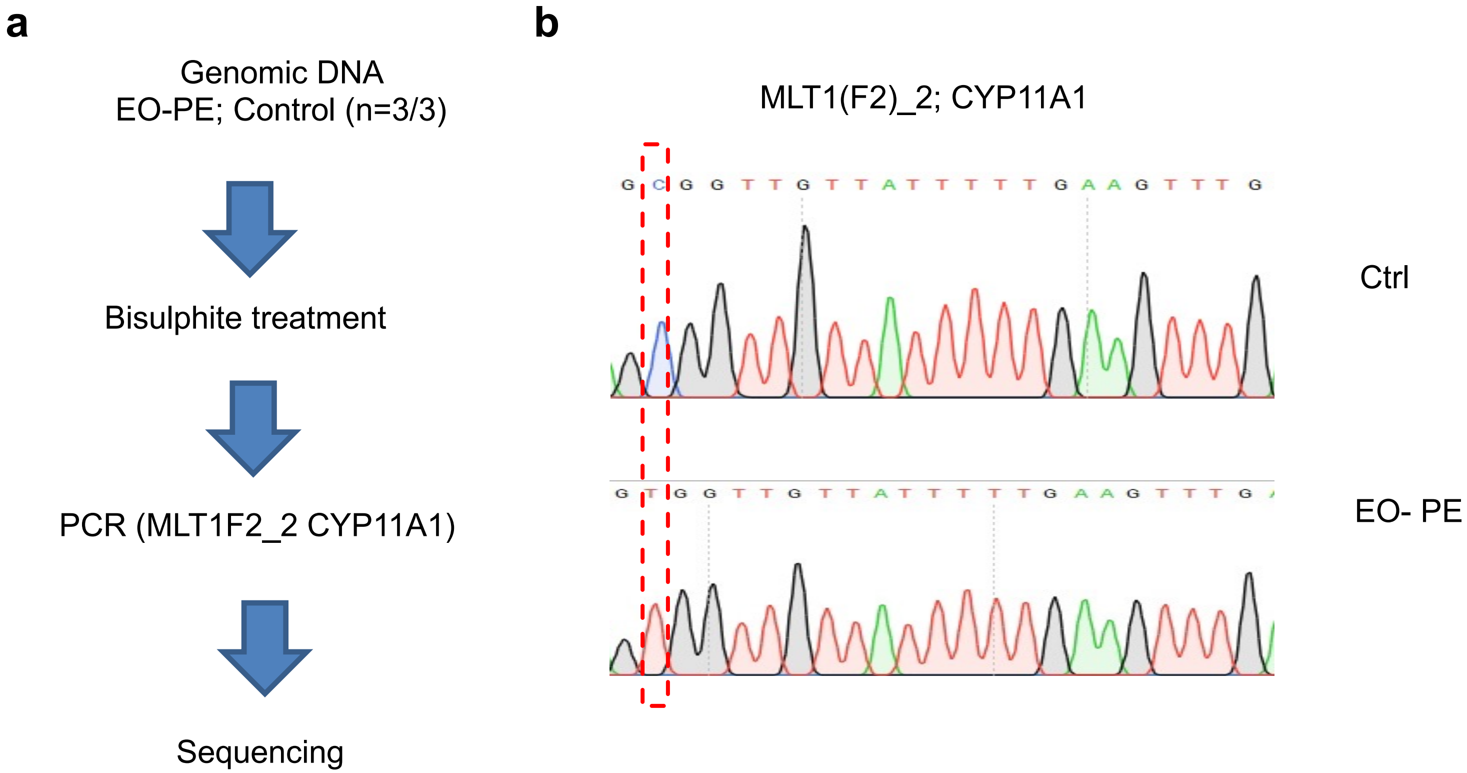

Fig. S6. Determination of the EPS8L1 interactome using quantitative mass spectrometry. (a) Schematics of the *Sleeping Beauty* transposon-based expression system used to transiently express EPS8L1-HA and EPS8L1 (control) in trophoblast cells for the interactome study. Inverted repeats; black arrows. (b) Significant EPS8L1 protein interactors in BeWo and SGHPL-4 cells.

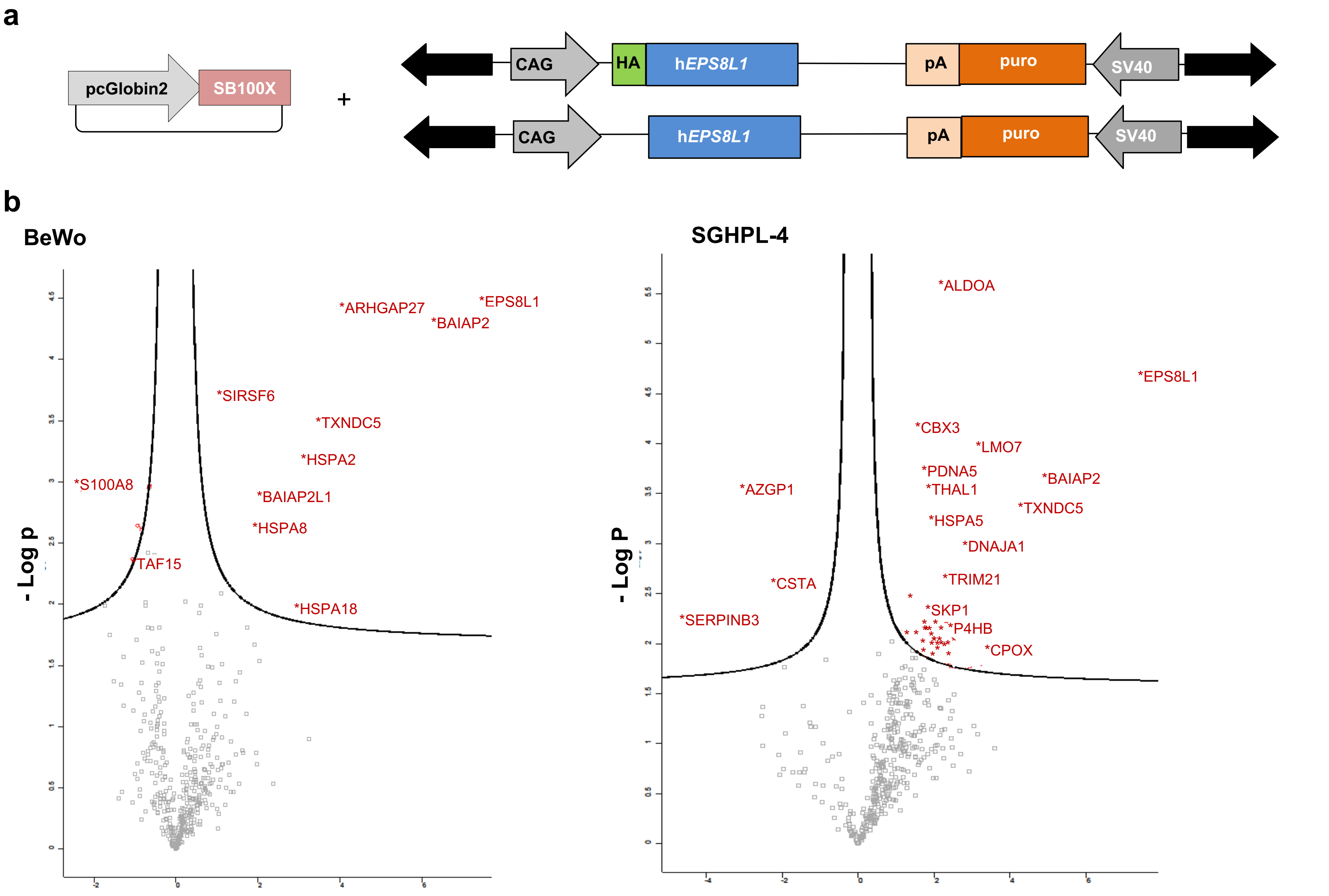

Fig. S7. EPS8L1 protein isoforms. Structure of the predicted EPS8L1 isoforms (Ensemble browser). The identified protein domains (Pfam) are indicated. The length (amino acid, aa), the molecular weight (MW) and the presence/absence of the putative transmembrane domain of the protein encoded isoforms are shown on the right. Note the unique exon 1 of the EPS8L1-205 isoform.

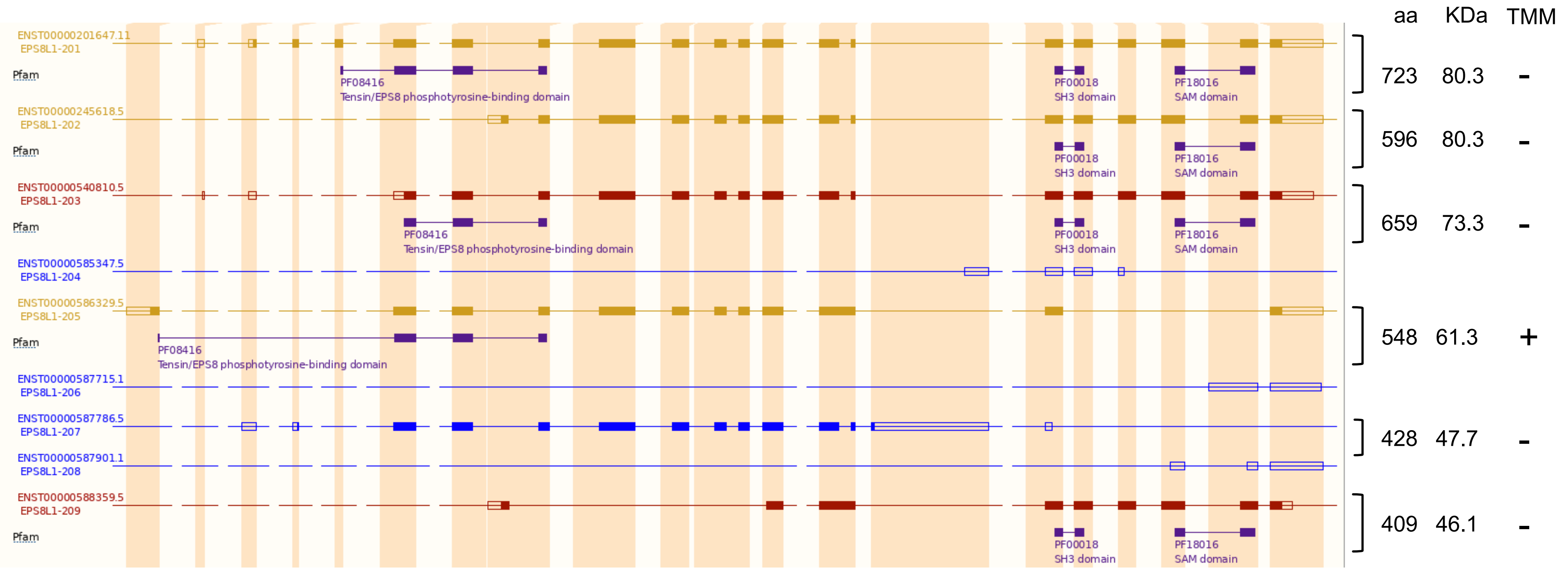

Fig. S8. EPS8L1 protein isoforms. (a) Transmembrane prediction (TMHMM posterior) probability analysis identifies a putative transmembrane domain in the EPS8L1-205 protein encoding isoform (black arrow), whereas the other isoforms, including EPS8L1-201 lacks this putative transmembrane domain. (b) Western blot analysis identified two secreted protein isoforms of EPS8L1 (black arrows) in the maternal plasma. 40 μg protein extracts were loaded for the placental lysates.

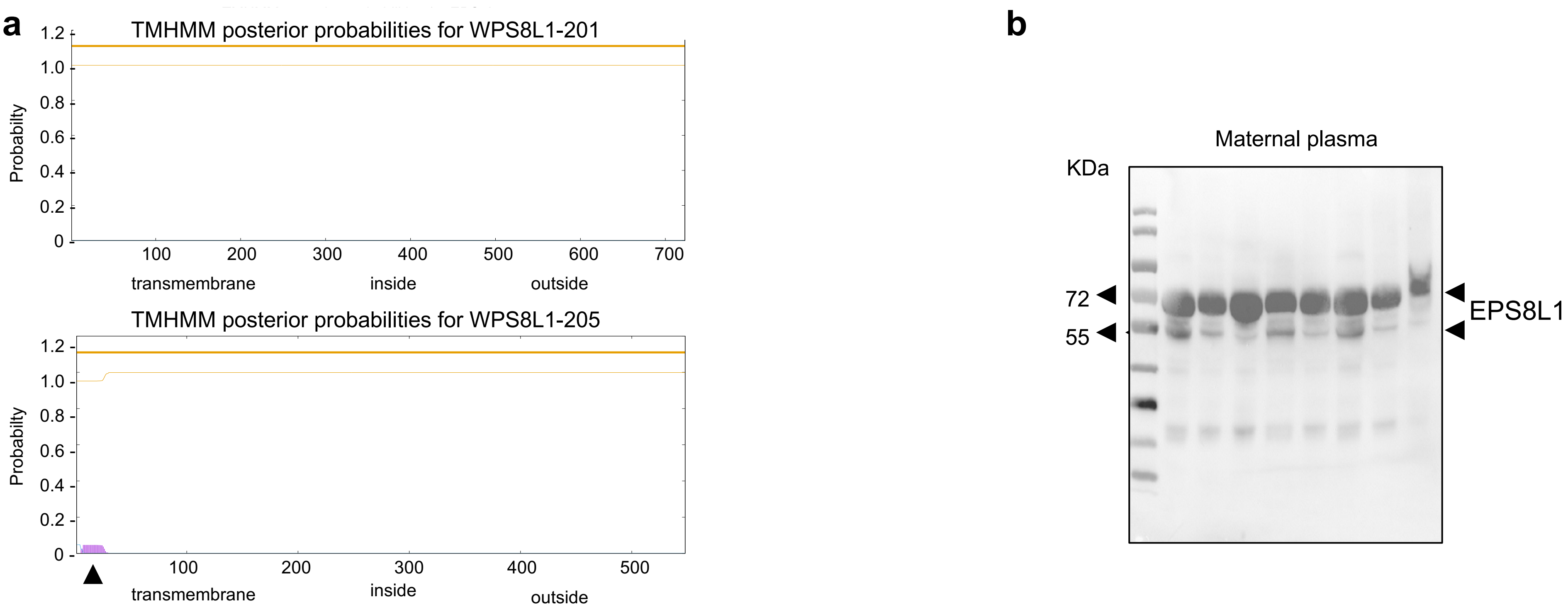

Fig. S9. Generation of the stable *in vitro* PE model. Fig. S8a. Schematic of the *Sleeping Beauty* transposon system (SB100X) used to (over)express the human HA-tagged EPS8L1 gene in SGHPL-4 cells. (b) Representative immunofluorescent staining of the control and OE-EPS8L1 overexpressing SGHPL-4 cells. EPS8L1, green; Actin, red; nucleus/DAPI, blue. Magnification, 60X. (c) Overexpression of HA-EPS8L1 was confirmed by qPCR (RNA-level) (n=3); mean ± SEM, **P ≤ 0.01, Unpaired t- test). (d) Overexpression of HA-EPS8L1 was confirmed by Western blotting (protein level). Anti-HA antibody (Lanes: replicates R1-R3; Ctrl1, WT lysate passage 15, Ctrl2, WT lysate, passage 16, ladder, L). Note the multiple EPS8L1 isoforms. Actin is used as a housekeeping protein.

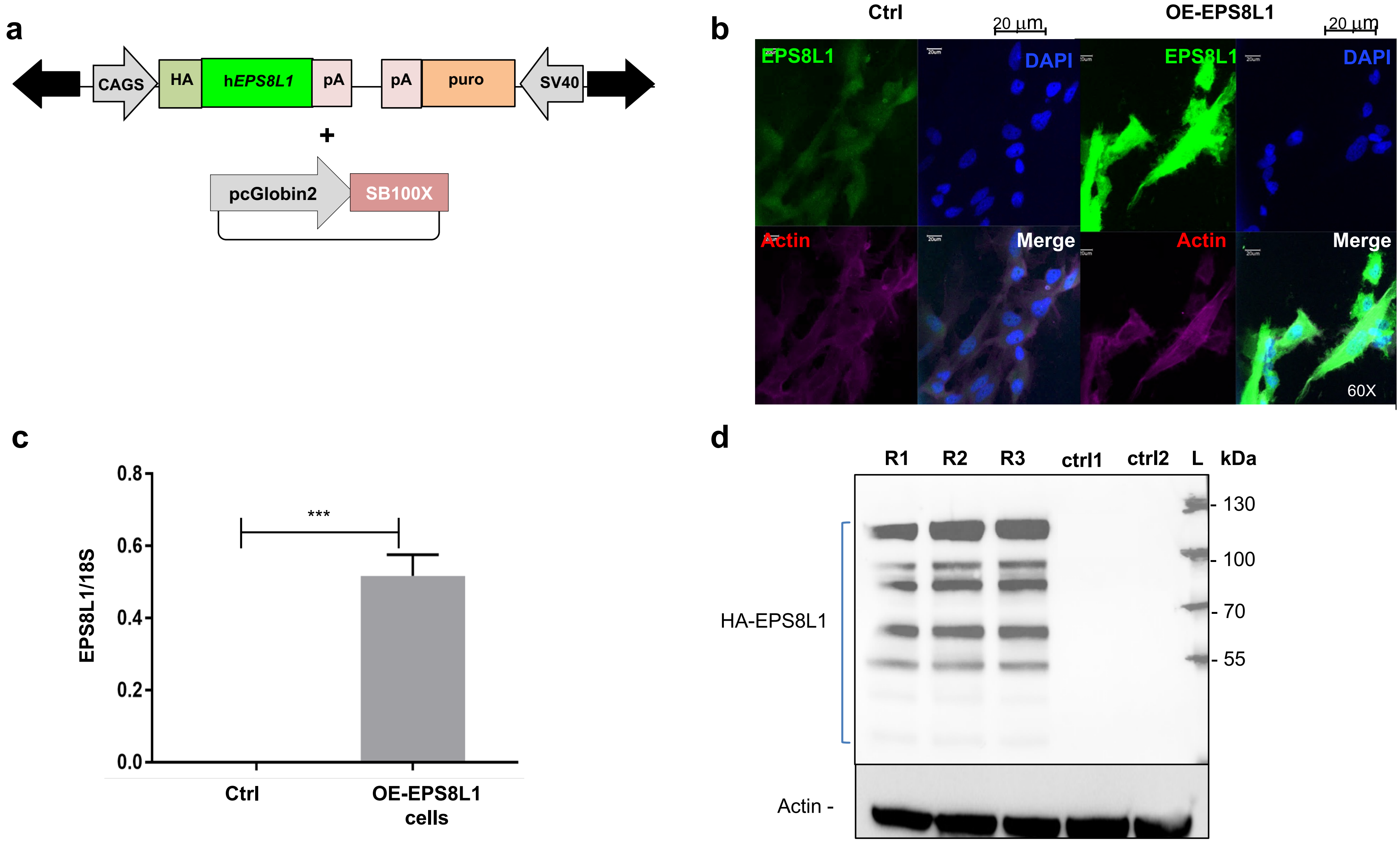

Fig. S10. Validation of differentially expressed genes (DEGs) identified in the *in vitro* PE model. qPCR validation of DEGs identified by RNA-seq in of OE-ESP8L1_ SGHPL-4 cells versus control SGHPL-4 cells (n=4). qPCR validated the significant downregulation of MMP2, MMP9, MYC and sFLT (n=4) and upregulation of CCND1 in the OE EPS8L1 SGHPL-4 cells, whereas no significant differences were found in the expression of MMP3 and PLGF in OE-EPS8L1_SGHPL-4 cells as compared to the control cells (n=4; mean ± SEM, *P ≤ 0.05, Unpaired t-test.)

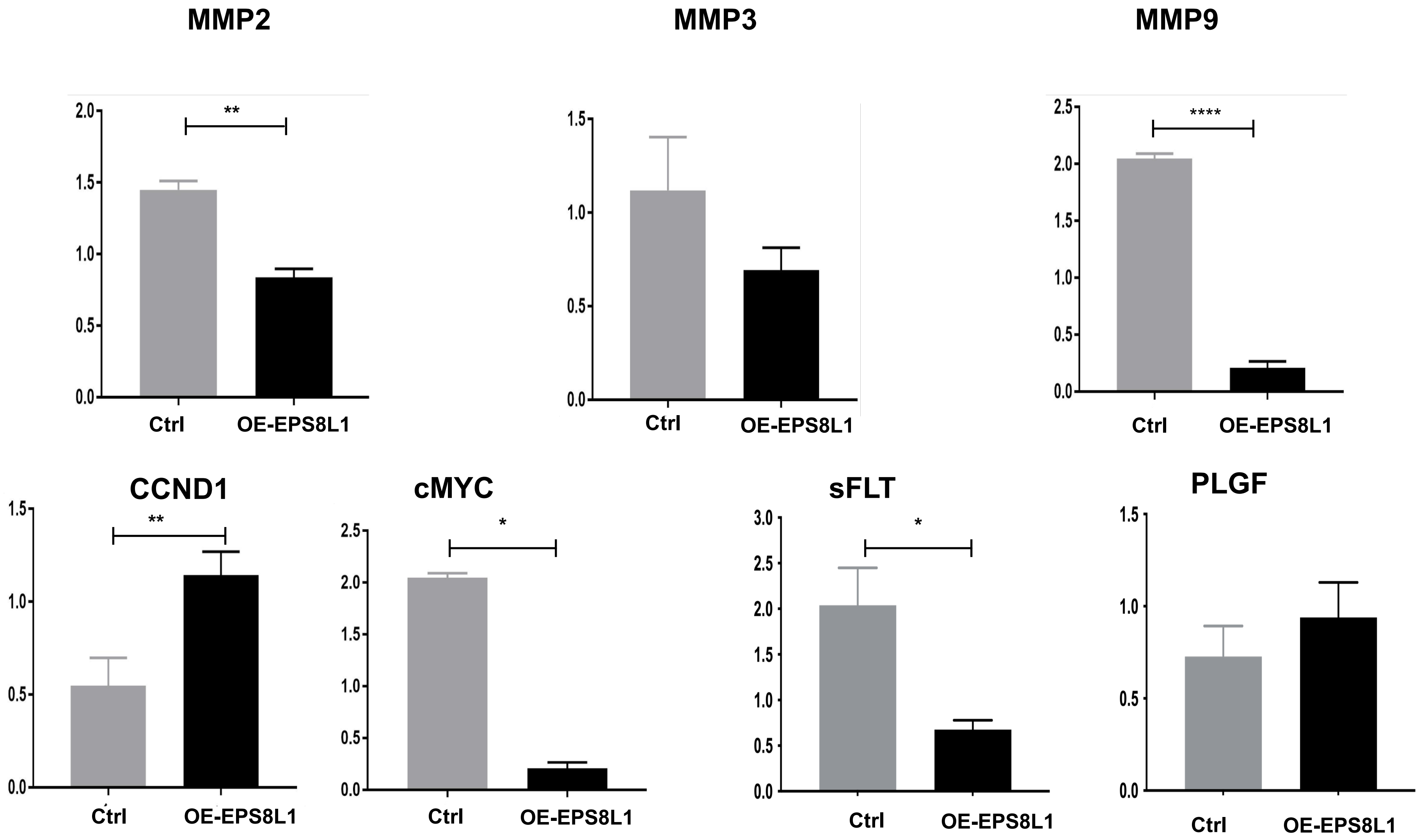

Fig. S11. Knocking out (KO) EPS8L1 in BeWo cells using the CRISPR/Cas9 technology. Western blot analysis was used to detect EPS8L1 expression in KO-EPS8L1 and control BeWo cells. sgRNA (sgRNA1-3) were transfected into BeWo cells, and EPS8L1 expression was analysed in comparison to controls: BeWo cells transfected with construct without sgRNA and non-transfected BeWo cells. Actin was used as an expression control (house-keeping gene). The experiment was repeated at least three times with technical replicates.

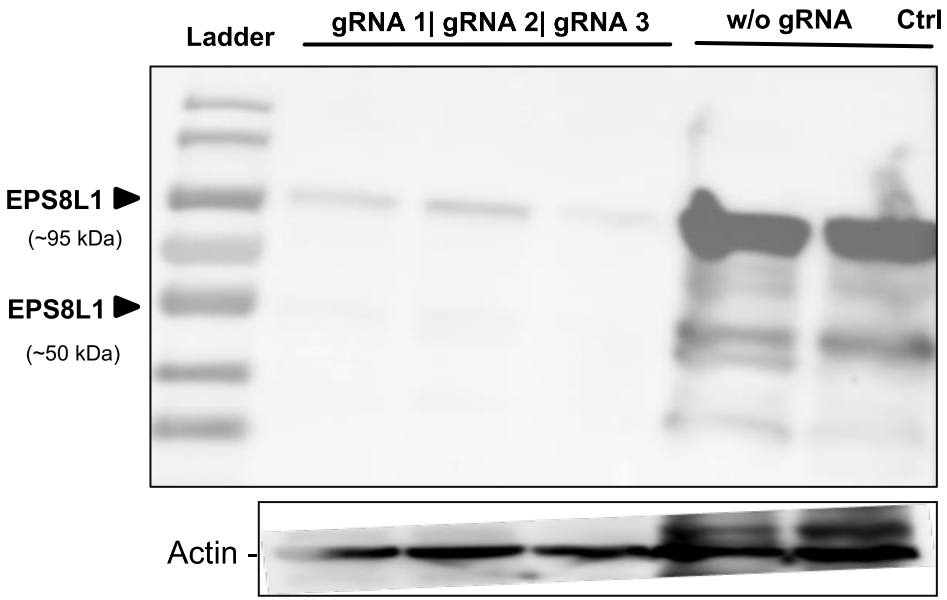

Supplement: Supplementary file 4 — Additional file 4: Supplementary Figures. [file 13059_2025_3821_MOESM4_ESM.pdf]
