# Supplementary material for: ERV3-MLT1 provides cis-regulatory elements for human placental functioning and are commonly dysregulated in human-specific preeclampsia
Source: Genome Biol. 2025 Nov 5;26:364. doi: 10.1186/s13059-025-03821-1 (PMC12587658; doi:10.1186/s13059-025-03821-1)
Supplement: Supplementary file 5 — Additional file 5: Knocking out MLT1G1 upstream EPS8L1. [file 13059_2025_3821_MOESM5_ESM.pdf]

MLT1G1 knock out strategy

gRNA design and plasmid construction

To generate targeted deletions at the MLT1G1 locus located upstream of the *EPS8L1* gene, two pairs of guide RNAs (gRNAs) were designed using the CRISPR Design Tool (https://crispor.gi.ucsc.edu/). One gRNA targeted the upstream region of MLT1G1, while the other was directed to the downstream region to enable efficient excision of the intervening sequence. For each gRNA, a pair of complementary 5′-phosphorylated oligonucleotides containing the gRNA sequence and BbsI restriction site overhangs were synthesized. The annealed oligonucleotides were ligated into the pX458 vector (Addgene #48138), which co-expresses SpCas9 and GFP, facilitating fluorescence-based selection. The accuracy of the gRNA insertions was verified by Sanger sequencing.

Generation of Knockout (KO) BeWo Cells

To generate MLT1G1 knockout (KO) BeWo cell lines, cells were transfected with the pX458-gRNA constructs using Lipofectamine 3000 (Thermo Fisher Scientific, Cat. #L3000015) following the manufacturer's instructions. After 48 hours, GFP-positive cells were enriched by fluorescence-activated cell sorting (FACS) using a BD FACSAria™ III cell sorter (BD Biosciences, San Jose, CA, USA). Sorted GFP-positive cells were plated into 6-well plates and cultured for 7–10 days to allow clonal expansion. Individual colonies were manually picked under a microscope in a laminar flow hood and genotyped after genomic DNA extraction using QuickExtract™ DNA Extraction Solution (Lucigen, Cat. #QE09050). The target region was amplified by PCR with locus-specific primers (F-MLT1G1 and R-MLT1G1), and products were analyzed via agarose gel electrophoresis. Colonies with homozygous deletions were further confirmed by Sanger sequencing and selected for downstream experiments. Wild-type bulk BeWo cells served as negative controls throughout the study.

Quantitative Real-Time PCR (qPCR) Analysis

To assess the regulatory role of MLT1G1 in endogenous *EPS8L1* expression, quantitative real-time PCR (qPCR) was performed to measure *EPS8L1* mRNA levels. Total RNA was extracted using the Direct-zol™ RNA MiniPrep Kit (Zymo Research, Cat. #R2025) following the manufacturer's protocol. One microgram of total RNA was then reverse-transcribed into complementary DNA (cDNA) using the High-Capacity RNA-to-cDNA™ Kit (Thermo Fisher Scientific, Cat. #4387406) according to the standard procedure. The resulting cDNA served as a template for qPCR, which was conducted using the SsoAdvanced™ Universal SYBR® Green Supermix (Bio-Rad, Cat. #1725271) on a CFX96 Touch™ Real-Time PCR Detection System (Bio-Rad, Hercules, CA, USA). Gene expression levels were calculated using the ΔΔCT method in CFX Maestro™ Software (Bio-Rad), with *GAPDH* used as the internal reference for normalization.

Results

To investigate the regulatory function of MLT1G1 in *EPS8L1* transcription, we designed two sgRNAs targeting regions upstream and downstream of the MLT1G1 locus (Fig. S3a). PCR amplification followed by Sanger sequencing confirmed a ~200 bp deletion between the two sgRNA target sites, validating successful genome editing (Fig. S3b-c). Owing to variability in Cas9 cleavage efficiency, some clones displayed minor differences in the precise deletion pattern within the target region (Fig. S3c). qPCR analysis revealed a significant reduction in *EPS8L1* mRNA levels in MLT1G1 knockout (BeWo-KO) cells compared to wild-type (BeWo-WT) controls ($P < 0.001$) (Fig. 3e). These results support the role of

MLT1G1 as a key regulator of *EPS8L1* transcription and suggest that its deletion directly affects gene expression.
