# Supplementary material for: ERV3-MLT1 provides cis-regulatory elements for human placental functioning and are commonly dysregulated in human-specific preeclampsia
Source: Genome Biol. 2025 Nov 5;26:364. doi: 10.1186/s13059-025-03821-1 (PMC12587658; doi:10.1186/s13059-025-03821-1)
Supplement: Supplementary file 6 — Additional file 6: Cell type specificity in human and mouse. [file 13059_2025_3821_MOESM6_ESM.pdf]

	**Human Protein Atlas (Human Cell Atlas / Single Cell Type Atlas)**
**1**	Glandular epithelial cells
**2**	Squamous epithelial
**3**	Specialized epithelial cells
**4**	Endocrine cells
**5**	Neuronal cells
**6**	Glial cells
**7**	Germ cells
**8**	Trophoblast cells - Cytotrophoblast (CTB)
**9**	Trophoblast cells - Syntiotrophoblast (STB
**10**	Trophoblast cells - Extravillous trophoblast (EVTB)
**11**	Endothelial cells
**12**	Muscle cells
**13**	Adipocytes
**14**	Pigment cells
**15**	Mesenchymal cells
**16**	Undifferentiated cellsBlood
**17**	Blood & immune cells

	**Mouse Cell Atlas**
**1**	Glandular epithelial cells
**2**	Squamous epithelial
**3**	Specialized epithelial cells
**4**	Endocrine cells
**5**	Neurons
**6**	Glial cells
**7**	Germ cells
**8**	N/A
**9**	Trophoblast subtypes - Trophoblast giant cells (TGC)
**10**	Trophoblast subtypes - Spongiotrophoblast (STP)
**11**	Endothelial cells
**12**	Muscle cells (skeletal, cardiac smooth)
**13**	Adipocytes (white and brown)
**14**	Melanocytes
**15**	Mesenchymal / stromal stem cells
**16**	Stem / progenitor cells
**17**	Heamtopoetic and immune cells

Note that the Human Cell Atlas (HCA) uses broad, conserved categories for classifying cell types. While most of these categories are well-conserved between humans and mice, placentation differs significantly between the two species. As a result, direct mapping to human trophoblast subtypes (STB, CTB, and EVTB) is not possible, although functionally analogous cell types do exist in

mice. These include trophoblast giant cells, spongiotrophoblasts, and syncytiotrophoblasts types I and II.
