# Supplementary material for: ERV3-MLT1 provides cis-regulatory elements for human placental functioning and are commonly dysregulated in human-specific preeclampsia
Source: Genome Biol. 2025 Nov 5;26:364. doi: 10.1186/s13059-025-03821-1 (PMC12587658; doi:10.1186/s13059-025-03821-1)

Multiz Alignments Configuration

Species selection: [+] [-]

[x] chimp	[x] bonobo	[x] gorilla	[x] orangutan	[x] gibbon
[ ] proboscis monkey	[ ] black snub-nosed monkey	[ ] golden snub-nosed monkey	[ ] angolan colobus	[x] crab-eating macaque
[x] rhesus	[x] baboon	[ ] pig-tailed macaque	[ ] sooty mangabey	[x] green monkey
[ ] drill	[x] squirrel monkey	[ ] ma's night monkey	[x] marmoset	[ ] white-faced sapajou
[x] tarsier	[ ] sclater's lemur	[ ] black lemur	[ ] coquerel's sifaka	[x] mouse lemur
[x] bushbaby	[x] mouse	[ ] dog	[ ] armadillo	

Multiple alignment base-level:

- [x] Display bases identical to reference as dots
- [x] Display chains between alignments

Codon Translation:

Default species to establish reading frame: hg38

- ( ) No codon translation
- (•) Use default species reading frames for translation
- ( ) Use reading frames for species if available, otherwise no translation
- ( ) Use reading frames for species if available, otherwise use default species

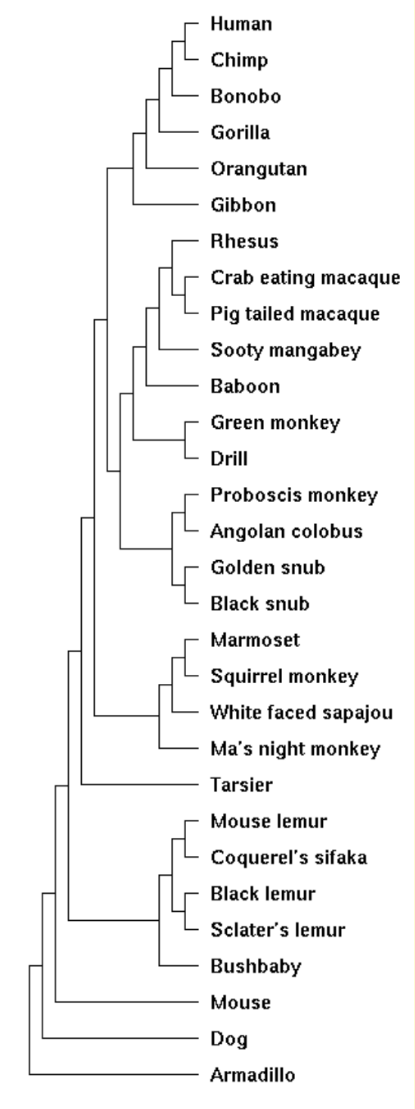

Table (a) Genome assemblies included in the 30-way Conservation track.

MAF format Multiz Alignment			
**Organism**	**Species**	**Release date**	**alignment type**
Human	Homo sapiens	Dec. 2013 (GRCh38/hg38)	MAF Net
Chimp	Pan troglodytes	May 2016 (Pan_tro 3.0/panTro5)	MAF Net
Bonobo	Pan paniscus	Aug. 2015 (MPI-EVA panpan1.1/panPan2)	MAF Net
Gorilla	Gorilla gorilla gorilla	Mar. 2016 (GSMRT3/gorGor5)	MAF Net
Orangutan	Pongo pygmaeus abelii	July 2007 (WUGSC 2.0.2/ponAbe2)	MAF Net
Gibbon	Nomascus leucogenys	Oct. 2012 (GGSC Nleu3.0/nomLeu3)	MAF Net
Rhesus	Macaca mulatta	Nov. 2015 (BCM Mmul_8.0.1/rheMac8)	MAF Net
Crab-eating macaque	Macaca fascicularis	Jun. 2013 (Macaca_fascicularis_5.0/macFas5)	MAF Net
Baboon	Papio anubis	Feb. 2013 (Baylor Panu_2.0/papAnu3)	MAF Net
Green monkey	Chlorocebus sabaeus	Mar. 2014 (Chlorocebus_sabeus 1.1/chlSab2)	MAF Net
Marmoset	Callithrix jacchus	March 2009 (WUGSC 3.2/calJac3)	MAF Net
Squirrel monkey	Saimiri boliviensis	Oct. 2011 (Broad/saiBol1)	MAF Net
Tarsier	Tarsius syrichta	Sep. 2013 (Tarsius_syrichta-2.0.1/tarSyr2)	MAF Net
Mouse lemur	Microcebus murinus	Feb. 2017 (Mmur_3.0/micMur3)	MAF Net
Bushbaby	Otolemur garnettii	Mar. 2011 (Broad/otoGar3)	MAF Net
Mouse	Mus musculus	Dec. 2011 (GRCm38/mm10)	MAF Net

MLT1G1- SPINT1 chr15:40838377-40838886

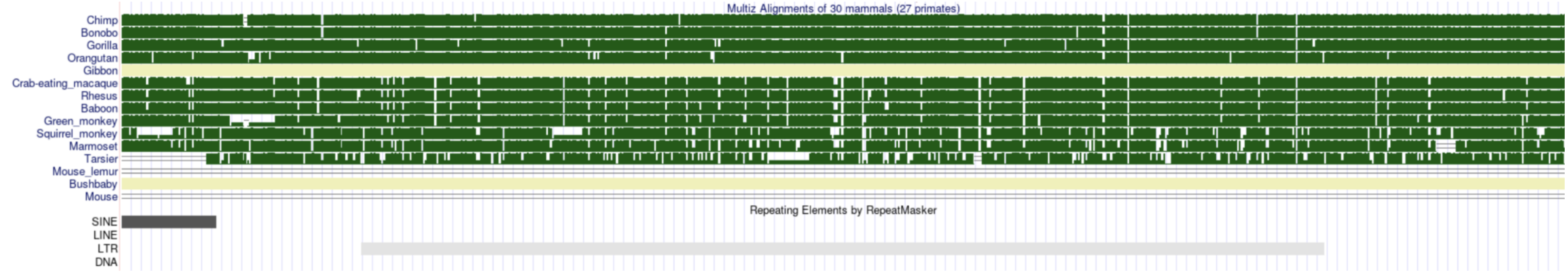

MLT1G1 EPS8l1 chr19:55066855-55067073

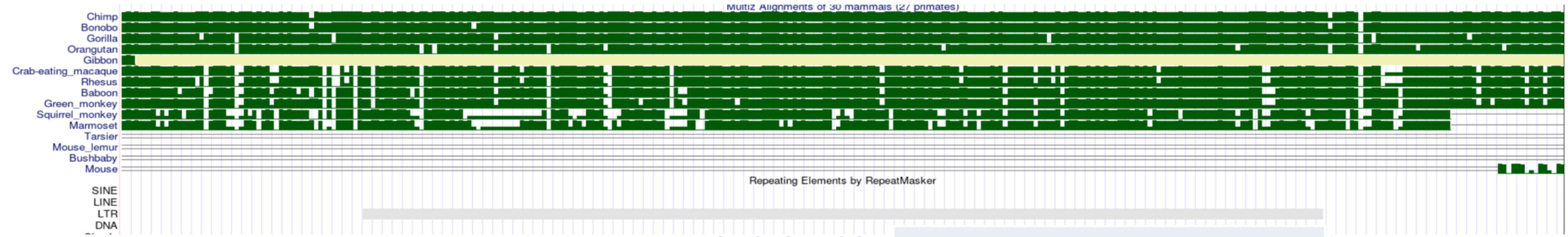

MLT1F2- SIGLEC6 chr19:51569654-51569772

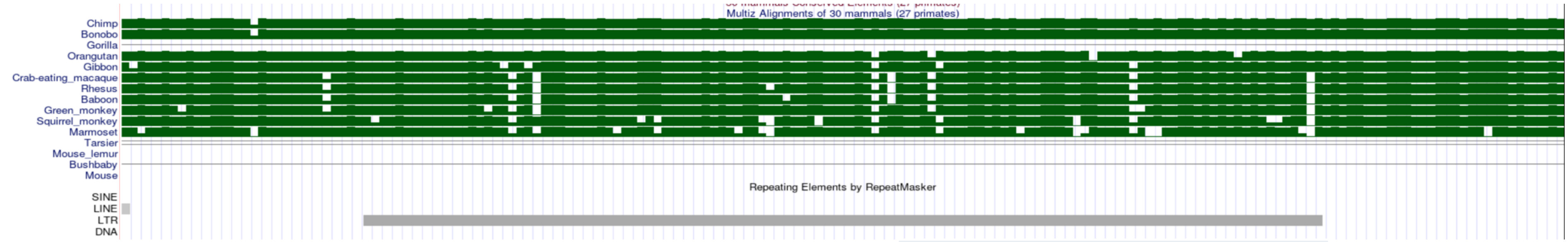

MLT2B4 - DACT2 chr6:168326994-168327219

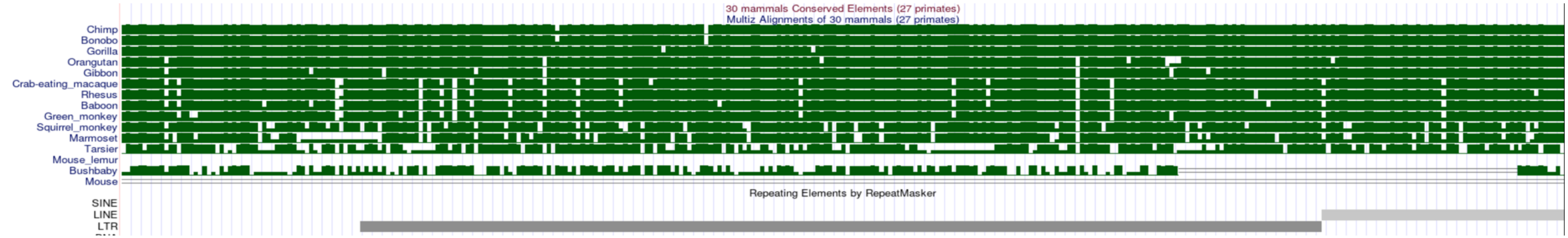

MLT1F2_2_CYP11A1 chr15:74369956-74370080

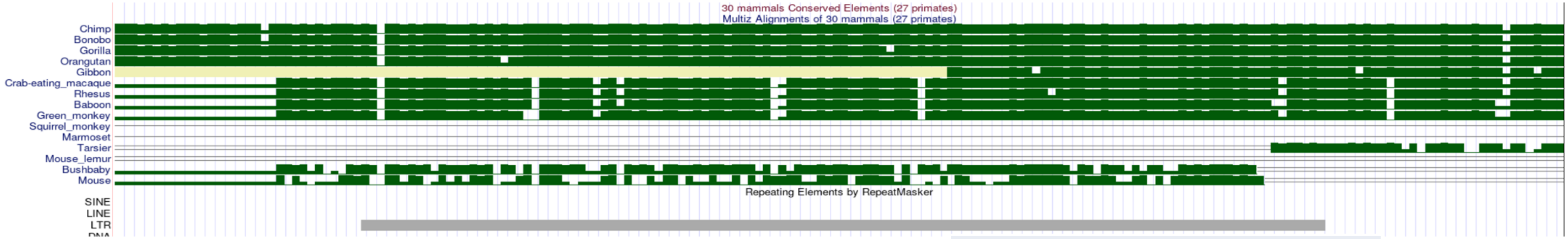

MLT1F2_1_CYP11A1 chr15:74369785-74369897

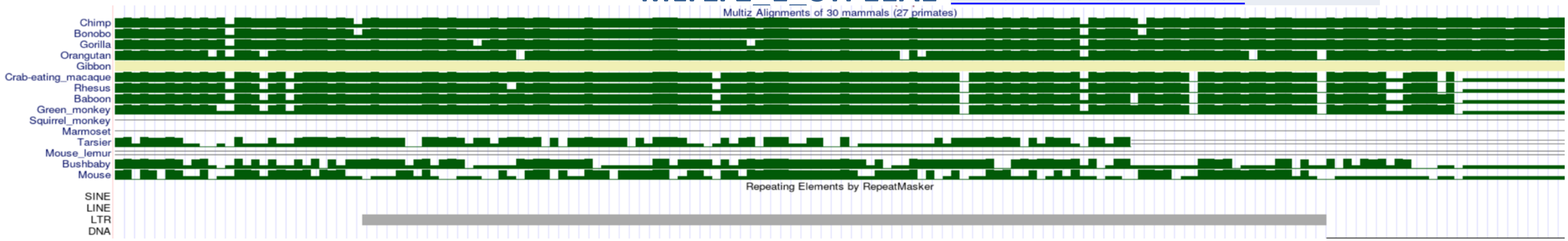

MLT1J_CYP11A1 chr15:74370498-74370834

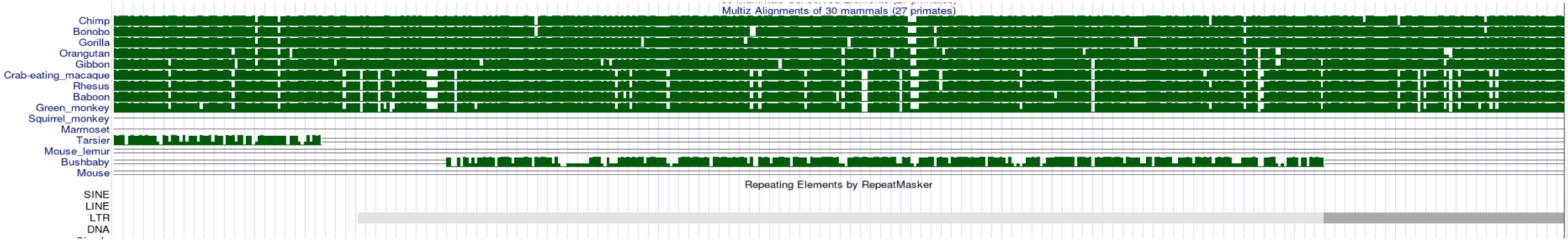

MLT1C_CSF2RB chr22:36907459-36907918

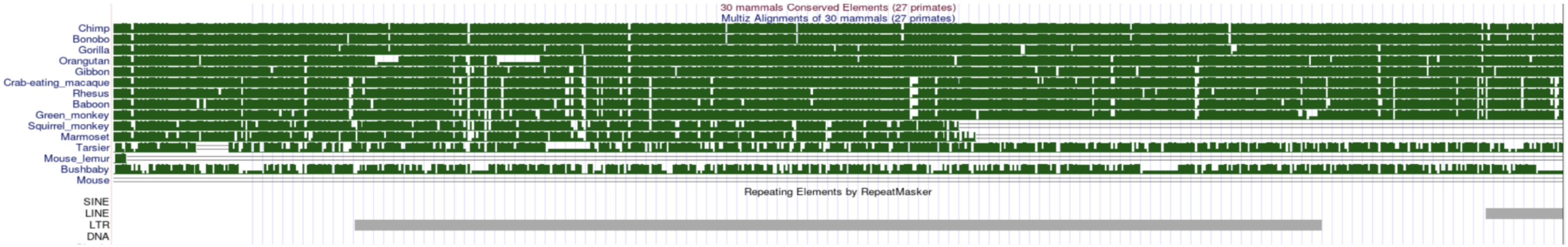

MLT1B1_CRH chr8:66185388-66185780

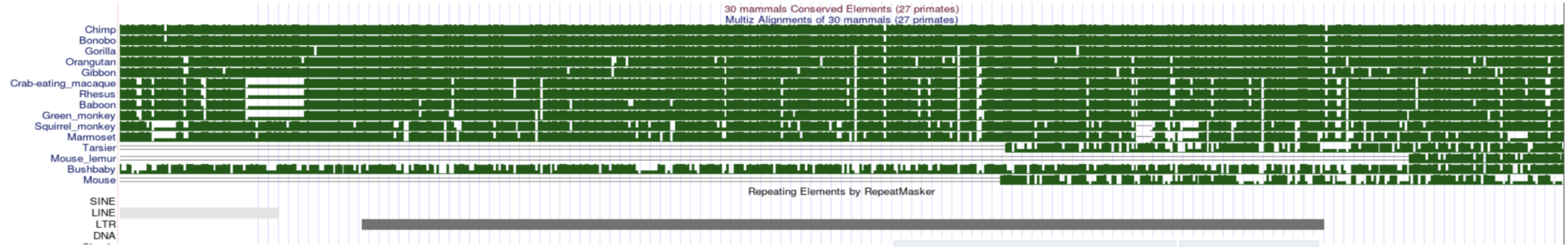

MLT1G3_CGA chr6:87092163-87092349

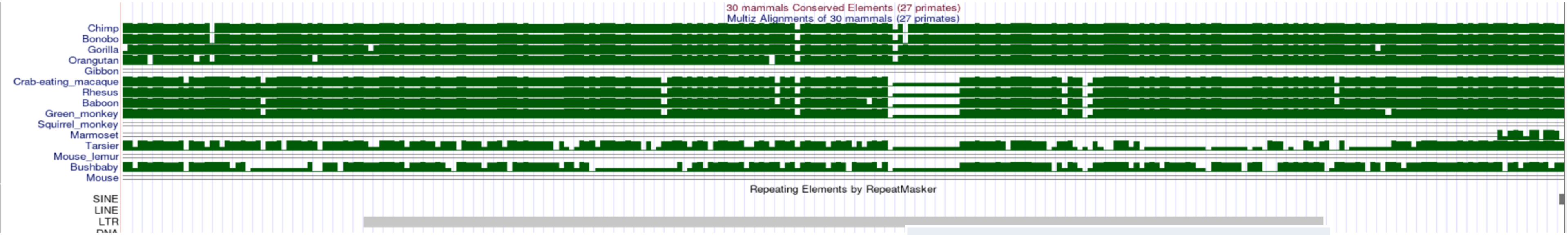

MLT1F2_ALDH3B2 chr11:67696398-67696552

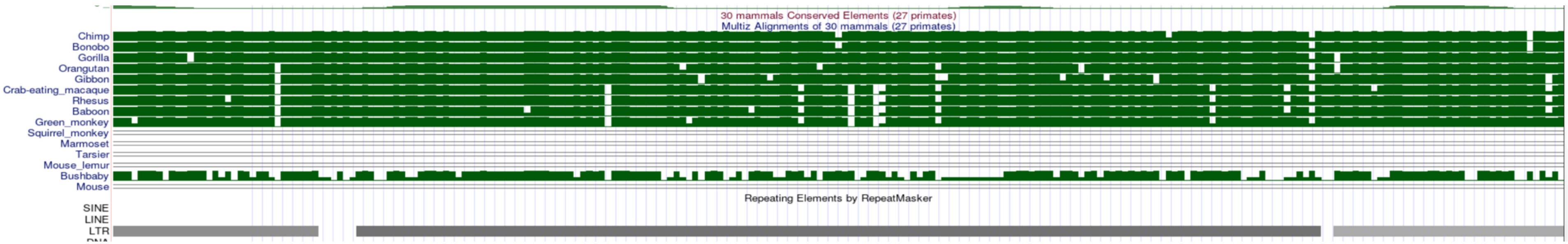

Supplement: Supplementary file 7 — Additional file 7: Evolutionary conservation analysis of MLT1/2. [file 13059_2025_3821_MOESM7_ESM.pdf]
