# Supplementary material for: ERV3-MLT1 provides cis-regulatory elements for human placental functioning and are commonly dysregulated in human-specific preeclampsia
Source: Genome Biol. 2025 Nov 5;26:364. doi: 10.1186/s13059-025-03821-1 (PMC12587658; doi:10.1186/s13059-025-03821-1)
Supplement: Supplementary file 8 — Additional file 8: Bisulfite sequencing. [file 13059_2025_3821_MOESM8_ESM.pdf]

MLT1F2 Length of sequence: 149 bp

MLT1F2 sequence:
GGGTGGCTGGGTGGCTGAGTGGCTCATGCTCAGGGTTTCTCACAAG**CG**GCTGCCATCCCTGAAGCTTGA
CCAGGGTGTAGGATCAGCTTCCAAGCTCAGTGGATTAAACTGGCCCTGCTTCAAAACTTTTCAGTGGCTG
TTTCAGGACT

MLT1F2 bisulfite converted sequence:
GGGTGGTTGGGTGGTTGAGTGGTTTATGTTTAGGGTTTTTTATAAG**CG**GTTGTTATTTTTGAAGTTTGAT
TAGGGTGTAGGATTAGTTTTTAAGTTTAGTGGATTAAATTGGTTTTGTTTTAAAATTTTTTAGTGGTTGTT
TTAGGATT

BS-CYP11A1-MLT1F2
Forward primer GGGTGGTTGGGTGGTTGAGTGGTTTATGTTTAG
Reverse primer CCTAAAACAACCACTAAAAAATTTTAAAACAAAACC

Sanger sequencing after cloning into pJET plasmid- Representative sequences after cloning sequences

PE placenta tissue
>TGGTTGGGTGGTTGAGTGGTTTATGTTTAGGGTTTTTTATAAG**TG**GTTGTTATTTTTGAAGTTTGACTAG
GGTGTAGGATTAGTTTTTAAGTTTAGTGGATTAAATTGGTTTTGTTTTAAAATTTTTTAGTGGTTGTTTTA
GGATCTTTCTAGAAGATCTCCTACAATATTCTCAGCTGCCATGGAAAATCGATGTTCTTCTTTTATTCTCTC
AAGATTTTCAGGCTGTATATTAAAACTTATATTAAGAACTATGCTAACCACCTCATCAGGAACCGTTGTA
GGTGGCGTGGGTTTTCTTGGCAATCGACTCTCATGAAAACTACGAGCTAAATATTCAATATGTTCCTCTT
GACCAACTTTATTCTGCATTTTTTTTGAACGAGGTTTAGAGCAAGCTTCAGGAAACTGAGACAGGAATTT
TATTAAAAATTTAAATTTTGAAGAAAGTTCAGGGTTAATAGCATCCATTTTTTGCTTTGCAAGTTCCTCAG
CATTCTTAACAAAAGACGTCTCTTTTGACATGTTTAAAGTTTAAACCTCCTGTGTGAAATTATTATCCGCTC
ATAATTCCACACATTATACGAGCCGGAAGCATAAAGTGTAAAGCCTGGGGTGCCTAATGAGTGAGCTAA
CTCACATTAATTGCGTTGCGCTCACTGCCAATTGCTTTCCAGTCGGGAAACCTGTCGTGCCAGCTGCATTA
ATGAATCGGCCAACGCGCGGGGAGAGGCGGTTTGCGTATTGGGCGCTCTTCCGCTTCCTCGCTCACTGA
CTCGCTGCGCTCGGTCGTTCGGCTGCGGCGAGCGGTATCAGCTCACTCAAAGGCG
GTAATACGGTTATCCACAGAATCAGGGGATAACGCAGGAAAGAACATGTGAGCAAAAGGCCAGCAAAA
GGCCAGGAACCGTAAAAAGGCCGCGTTGCTGGCGTTTTTCCATAGGCTCCGCCCCCCTGACGAGCATCA
CAAAAATCGACGCTCAAGTCAGAGGTGGCGAAACCCGACAGGACTATAAAGATACCAGGCGTTTCCCCC
TGGAAGCTCCCTCG

Healthy placenta tissue
>TGGTTGGGTGGTTGAGTGGTTTATGTTTAGGGTTTTTTATAAG**CG**GTTGTTATTTTTGAAGTTTGATTAG
GGTGTAGGATTAGTTTTTAAGTTTAGTGGATTAAATTGGTTTTGTTTTAAAATTTTTTAGTGGTTGTTTTA
GGATCTTTCTAGAAGATCTCCTACAATATTCTCAGCTGCCATGGAAAATCGATGTTCTTCTTTTATTCTCTC
AAGATTTTCAGGCTGTATATTAAAACTTATATTAAGAACTATGCTAACCACCTCATCAGGAACCGTTGTA
GGTGGCGTGGGTTTTCTTGGCAATCGACTCTCATGAAAACTACGAGCTAAATATTCAATATGTTCCTCTT
GACCAACTTTATTCTGCATTTTTTTTGAACGAGGTTTAGAGCAAGCTTCAGGAAACTGAGAC

AGGAATTTTATTAAAAATTTAAATTTTGAAGAAAGTTCAGGGTTAATAGCATCCATTTTTTGCTTTGCAAG
TTCCTCAGCATTCTTAACAAAAGACGTCTCTTTTGACATGTTTAAAGTTTAAACCTCCTGTGTGAAATTATT
ATCCGCTCATAATTCCACACATTATACGAGCCGGAAGCATAAAGTGTAAAGCCTGGGGTGCCTAATGAG
TGAGCTAACTCACATTAATTGCGTTGCGCTCACTGCCAATTGCTTTCCAGTCGGGAAACCTGTCGTGCCA
GCTGCATTAATGAATCGGCCAACGCGCGGGGAGAGGCGGTTTGCGTATTGGGCGCTCTTCCGCTTCCTC
GCTCACTGACTCGCTGCGCTCGGTCGTTCGGCTGCGGCGAGCGGTATCAGCTCACTCAAAGGCGGTAAT
ACGGTTATCCACAGAATCAGTGGATAACGCAGGAAAGAACTGTGGAGCAAAAGGCCAGCAAAAGGCCA
GTAACCGTAAAAAGGCCG
