# Supplementary material for: ERV3-MLT1 provides cis-regulatory elements for human placental functioning and are commonly dysregulated in human-specific preeclampsia
Source: Genome Biol. 2025 Nov 5;26:364. doi: 10.1186/s13059-025-03821-1 (PMC12587658; doi:10.1186/s13059-025-03821-1)
Supplement: Supplementary file 12 — Additional file 12: Patient and clinical data. [file 13059_2025_3821_MOESM12_ESM.pdf]

## Patients and clinical data

The following cohorts were used in this study:

*Oslo cohort:* The placental samples are comprising of patient samples from bio-bank collection at Oslo University Hospital, Norway. The Oslo cohort consists of placental tissues collected during elective Cesarean sections in 24 early onset (EO) PE (delivery < 34 gestational weeks) and 27 controls with normotensive and uncomplicated pregnancies. The uncomplicated pregnancy group consisted of healthy, normotensive women undergoing elective Cesarean section due to breech presentation or other reasons. The Oslo Pregnancy Biobank samples stem from an ongoing recruitment of pregnant patients. The study (and the present research collaboration) is approved by the Regional Committee of Medical Research Ethics South Eastern Norway (ref: 2013/2092 and 529-02162). Informed written consent was obtained from each participant.

| Oslo Characteristics at delivery | Controls (n=27) | EO-PE (n=24) | Statistics |
|---|---|---|---|
| Maternal age (Years) | 31.2 ± 4.2 | 31.6 ± 5.6 | P=0.63 |
| BMI (kg/m$^2$) | 28.6± 3.4 | 31.5 ± 5.2 | **P ≤ 0.01 |
| Gestational week | 39.0 ± 0.9 | 33.7 ± 3.7 | ***P ≤ 0.001 |
| Systolic BP (mm Hg) | 119 ± 11.6 | 165 ± 16.2 | ***P ≤ 0.001 |
| Diastolic BP (mm Hg) | 72.1 ± 10.9 | 101.1 ± 6.7 | ***P ≤ 0.001 |
| Baby weight (g) | 3492± 402 | 2170 ± 1005 | ***P ≤ 0.001 |

*Essen cohort:*

EO-PE (n-=10) and healthy control (n=7) placental samples were collected. For this study pregnant women were recruited at the Department of Gynecology and Obstetrics, University Hospital Essen, Germany. The respective ethics committee approved the study according to the Helsinki Declaration on ethical principles for medical research involving human subjects by obtaining consent forms (Amendment No: 12-5212-BO). Multiple pregnancies, fetal anomalies, and infections were excluded from the study. Clinical characteristics of EO-PE and healthy

control cases from the study population are shown in the table below. Data are presented as mean ± standard error of mean (SEM).

| Essen Characteristics at delivery | Control early (n=7) | EO-PE (n=10) | Statistics |
|---|---|---|---|
| Maternal age (Years) | 32.4 ± 2.2 | 31.0 ± 1.5 | P=0.5917 |
| BMI ($kg/m^2$) | 29.4 ± 2.2 | 31.7 ± 3.1 | P=0.7727 |
| Gestational week | 32.0 ± 1.4 | 30.6 ± 0.6 | P=0.3229 |
| Systolic BP (mm Hg) | 115.9 ± 5.9 | 162.4 ± 5.5 | ***P ≤ 0.001 |
| Diastolic BP (mm Hg) | 67.1 ± 4.7 | 95.8 ± 4.8 | ***P ≤ 0.001 |
| Baby weight (g) | 1888 ± 350.9 | 1290 ± 119.4 | P=0.0841 |

**Graz-Aachen Cohort:** A total of 141 healthy placental samples, 92 samples from early gestation (Graz) and 49 samples at term were collected in the University Hospital of the RWTH Aachen, Germany. Sampling was approved by the local ethical committee (EK 148/07) and informed consent was obtained from each participating woman. Placenta tissue from early gestational age (with GA 5 – 11 weeks) were collected from electively terminated gravidities with informed consent of healthy patients. Exclusion criteria were a maternal age under 18, maternal BMI >25, maternal pathologies (self-reported). Ethical approval was obtained from the Medical University of Graz Ethics Committee (31-019 ex 18/19; 26-132 ex 13/14). Immediately after delivery, a random tissue sample (1x1x1cm) of the medial third of the placenta was cut from vital cotyledons that were macroscopically free of infarct areas or other obvious pathologies. Amnion and decidua were dissected and remaining tissue were rinsed twice in NaCl 0.9% 4°C to remove blood and then snap frozen in liquid nitrogen and stored at -80°C until processing. Table shows values in mean (SD). were analysed by qPCR for the EPS8L1 expression throughout the gestation.

| Graz-Aachen Characteristics | All (n=141) | Early (n=92) | Term (n=49) |
|---|---|---|---|

| at delivery | | | |
|---|---|---|---|
| Maternal age (Years) | 28 ± 6.0 | 26 ± 5.0 | 32 ± 5.0 |
| BMI (kg/m$^2$) | 22.52 ± 3.48 | 21.61 ± 2.39 | 24.22 ± 4.46 |

*Charité-Aachen cohort:*

Preeclamptic and IUGR term placenta samples and gestational age matched controls were collected in the University Hospital of the RWTH Aachen, Germany (as described above) and at Charité – Universitätsmedizin Berlin. Sampling was approved by the local ethical committees (Aachen: EK 148/07; Berlin: EA2/132/12) and informed consent was obtained from each participating woman. Clinical characteristics of the PE, IUGR and control cases from the study population are listed in the table.

| Charite/Aachen Characteristics at delivery | Control early (n=36) | Control late (n=65) | IUGR early (n=28) | IUGR late (n=13) | PE Early onset (n=14) | PE late onset (n=23) |
|---|---|---|---|---|---|---|
| Maternal age (Y) | 31.4 ± 5.7 | 32.5 ± 5.6 | 27.7 ± 6.4 | 27.6 ± 5.8* | 33.0 ± 5.3 | 31.2 ± 6.2 |
| BMI (kg/m$^2$) | 25.0 ± 6.1 | 23.6 ± 4.6 | 24.0 ± 4.8 | 26.2 ± 7.0 | 26.0 ± 5.1 | 27. ± 7.0* |
| Gestational days | 203.3 ± 22.1 | 264.7 ± 13.0 | 208.6 ± 19.0 | 264.3 ± 11.8 | 206.1 ± 21.3 | 254.5 ± 12.0 |
| Systolic BP (mm Hg) | 115 ± 9.7 | 116.6 ± 10.8 | 122.9 ± 13.7*** | 104.5 ± 15.5* | 152.6 ± 12.4*** | 153.5 ± 14.5*** |
| Diastolic BP (mm Hg) | 65.0 ± 8.0 | 69.7 ± 8.1 | 70.7 ± 11.7*** | 65.2 ± 10.3 | 95.9 ± 8.7*** | 94.8 ± 8.8*** |

Data are presented as mean ± standard deviation. *P ≤ 0.05, ***P ≤ 0.001 vs. control.

***Charité*-Berlin cohort:** In addition to the placenta samples collected from patients in doi: 10.1161/CIRCULATIONAHA.117.028110, a further three control and five EO-PE placentae were obtained from HELIOS Klinikum Berlin-Buch with the approval of the Regional Committee of the

Medical Faculty of Charité Berlin (making a total of ten EO-PE and eight healthy placentae). EO-PE patients were defined as having hypertension (systolic blood pressure (SBP) ≥140 mmHg or diastolic blood pressure (DBP) ≥90 mmHg) and proteinuria (≥0.3 g in a 24-hour urine specimen) before the 34(th) week of pregnancy. The gestational age was 30.5 ± 4.5 weeks. Control patients were defined as having had an uncomplicated term pregnancy. All samples were collected and processed within two hours of Caesarean section delivery.

*Manchester Antenatal Vascular Service Cohort:*
Maternal plasma samples included in this study were collected from 24th - 28th weeks of gestation from a high-risk cohort in the United Kingdom, the Manchester Antenatal Vascular Service (The MAViS clinic). Informed written consent was collected from each participant. The study was approved by the NRES (National Research Ethics Service) Committee North West 11/NW/0426. The inclusion criteria for women in the MAViS study were: (i) chronic hypertension BP ≥140/90 at ≤20 weeks; (ii) chronic hypertension requiring antihypertensive treatment ≤20 weeks; (iii) pre-gestational diabetes mellitus with evidence of vascular complications (hypertension, nephropathy); (iv) history of ischaemic heart disease; and (v) previous early onset preeclampsia.

*Non-Pregnant female Cohort:* Plasma samples from 6 non-pregnant healthy females were used from this cohort for the EPS8L1 ELISA. The women in this cohort have no self-reported medical history and the age of all the women were 25-30 years old. The samples were collected at Berlin. Sampling was approved by the local ethical committee (EA2/046/17) and informed consent was obtained from each participating woman. Women taking any medications (birth control pills) were not included in this study.

## Samples, used in the current study

- Charité-Berlin cohort: trophoblast samples, purified by Julia Zadora, Rabia Anwar and Katarina Stevanovic. These samples were subjected to bulk RNA sequencing.
- Oslo cohort: *cDNA, genomic DNA samples*, provided by Anne C. Staff. Note that the differential expression of the short-listed genes was assessed in this cohort. cDNA from fat (n=5), decidua (n=5), macrophages (n=5) and muscle tissues (n=5) were also collected from the same cohort. The levels of serum PIGF and sFLT1 of patients were measured on Elecsys (Roche Diagnostics) by the HELIOS Klinikum GmbH.
- Manchester Antenatal Vascular Service Cohort: *Plasma samples*- provided by Jenny Myers. A total of 24 samples were used from this cohort, including 12 controls and 12 PE pregnant serum samples.

- Non-Pregnant female Cohort: Plasma Berlin NPS Cohort: Serum samples from non-pregnant healthy females (n=6) were used from this cohort for the EPS8L1 ELISA.
- Charité - *cDNA samples*, provided by Stefan Verlohren.
- Graz-Aachen Cohort- *cDNA samples,* provided by Ulrich Pecks, Martin Gauster and Olivia Nonn.
- Essen cohort- *FFPE tissue blocks*- provided by Sandra M. Blois and Alex Gellhaus.
