# Supplementary material for: ERV3-MLT1 provides cis-regulatory elements for human placental functioning and are commonly dysregulated in human-specific preeclampsia
Source: Genome Biol. 2025 Nov 5;26:364. doi: 10.1186/s13059-025-03821-1 (PMC12587658; doi:10.1186/s13059-025-03821-1)
Supplement: Supplementary file 13 — Additional file 13: Primers used in the study. [file 13059_2025_3821_MOESM13_ESM.pdf]

Primers used in the study

	Gene	Primer	Sequence (5' > 3')
q-PCR	*ALDH3B2*	Forward	ATGAAGGATGAACCACGGTCC
		Reverse	GTTCCAGGGTGCGATGATGA
	CSF2RB	Forward	CTCGTCAACGTGACCCTCAT
		Reverse	CGACAAAACTCTGGCAGGGA
	CYP11A1	Forward	CTTCACCCCATCTCCGTGAC
		Reverse	GTCTTTGCTCAGCCATCGG
	PHYHIPL	Forward	GTGCAGACTGCCTCAAAACA
		Reverse	AAAACTTAAGCATGCGTCCTG
	SPINT1	Forward	AAGGTACAACCCCAGGAACC
		Reverse	CCTCTGGGTGGTCTGAGCTA
	DACT2	Forward	CGGTCGGTTGATGAGACTACT
		Reverse	CAGGGCTCTGTCAAGATCACC
	SLC22A11	Forward	TATTAAGGGCAAACCAGACCAAG
		Reverse	CCAGCCCATAGTAGGAGATCAA
	EPS8L1	Forward	GAGAGCTTTGTATCGAGGCTG
		Reverse	GCCCGAAAAGGAAGTGCAAC
	PLEKHA8	Forward	AGCCTCGATGGTTCCTTCTCT
		Reverse	TCAGGTCCATGCGTGTATTATCT
	RAD51	Forward	CAACCCATTTCACGGTTAGAGC
		Reverse	TTCTTTGGCGCATAGGCAACA
	KIF23	Forward	TGGTTCCTACATTCAGAAATGAGA
		Reverse	CGTTCTGATCAGGTTGAAAGAGTA
	NRK	Forward	CATTGGCCTTGGTACTTATGGC
		Reverse	GTCTTACGAGCGTTCATCACTT
	C1QTNF6	Forward	GAAAGGGTCTTTGTGAACCTTGA
		Reverse	CTGCGCGTACAGGATGACAG
	EPS8	Forward	TGAATGGCTACGGATCATCACC
		Reverse	CACTGTCCCGTGCATAATTCT
	MYC	Forward	GTCAAGAGGCGAACACACAAC
		Reverse	TTGGACGGACAGGATGTATGC

	CCND1	Forward	GCTGCGAAGTGGAAACCATC
		Reverse	CCTCCTTCTGCACACATTTGAA
	MMP9	Forward	AGACCTGGGCAGATTCCAAAC
		Reverse	CGGCAAGTCTTCCGAGTAGT
Housekeeping gene	*18S*	Forward	ACATCCAAGGAAGGCAGCAG
		Reverse	TTTTCGTCACTACCTCCCCG
	GAPDH	Forward	AGCCACATCGCTCAGACAC
		Reverse	GCCCAATACGACCAAATCC
HA-*EPS8L1* construct	h*EPS8L1*-EcoRV	Forward	acgtcccagactacgctgatatcATGAGCACCGCCACAGGC
	h*EPS8L1*-NotI	Reverse	aataaacaagttaacaacgcggccgcTCAAATGACCTCCATTTCCACCTCG
NonHA-*EPS8L1* construct	h*EPS8L1*-SgrAI	Forward	CCCGGAGCGCCGGCGGCTGTC
	h*EPS8L1*-EcoRV	Reverse	CGGGATATCGGTGAATTCTTT
LTR-construct (Gibson Assembly)	*CYP11A1*-MLT1F2-1	Forward	catggctttagaagcttgatGGGTGGCTGGGTGGCTGA
		Reverse	cactagtgaattccatggatAGTCCTGAAACAGCCACTGAAAAGTTTTG
	CYP11A1-MLT1F2-2	Forward	catggctttagaagcttgatTGTATTAGCTGTTTACTGCTG
		Reverse	cactagtgaattccatggatAGTCACCCAGAAAAGCTG
	CYP11A1-MLT1J	Forward	catggctttagaagcttgatATGTGGTTAAGTTCCCAC
		Reverse	cactagtgaattccatggatACAGAAAATCCAAATCACAG
	ALDH3B2-MLT1F2	Forward	catggctttagaagcttgatAATGCTGCAGCCAAGCTG
		Reverse	cactagtgaattccatggatACTAATACAATGGCAAAAAGTAACAGG
	SPINT1-MLT1F2	Forward	catggctttagaagcttgatATTCCAAAAGTTACCAGCTTG
		Reverse	cactagtgaattccatggatTGTGGTAGGTTGCTTCTAAATG
	CSF2RB-MLT1C	Forward	catggctttagaagcttgatTGTTATGAGGTTGGCTTG
		Reverse	cactagtgaattccatggatTGTGTCAATTTCCTAGGG
	DACT2-MLT2B4	Forward	catggctttagaagcttgatTGTGATGATTAATTTTATGTGTCAAC
		Reverse	cactagtgaattccatggatAAGAATCCCTCTTGGTGAAG
	DACT2-MER41D	Forward	catggctttagaagcttgatTTAGGGAATCAGGAGCCC

		Reverse	cactagtgaattccatggatTGTTACCAGAAAAGCAGTC
	EPS8L1-MLT1G	Forward	catggctttagaagcttgatAAGGAGCTCCAGTGGTCC
		Reverse	cactagtgaattccatggatTTCCAGTTACTATGGCCATATAACAAATTATTC
Sequencing primers	h*EPS8L1*-F1	Forward	ACAATTACCGCTCGGGCCGC
	h*EPS8L1*-F2	Forward	GAGGCCGAGTACACCGACGT
	h*EPS8L1*-F3	Forward	ATCTGAGCCTCAGCTGGAGT
	HA Seq-F3	Forward	ATGGTAATCGTGCGAGAG
Bisulphite sequencing	MLT1F2-2	Forward	TGTATTAGTTGTTTATTGTTGTTTAATAAATTA
		Reverse	AATCACCCAAAAAAACTACTCCTAAATACTACA
	BS-CYP11A1-MLT1F2	Forward	GGGTGGTTGGGTGGTTGAGTGGTTTATGTTTAG
		Reverse	CCTAAAACAACCACTAAAAAATTTTAAAACAAAACC
EPS8L1-KO	gRNA-1		ACAATTACCGCTCGGGCCGC
	gRNA-2		CACAATTACCGCTCGGGCCG
	gRNA-3		CAATTACCGCTCGGGCCGCG
