# Supplementary material for: ERV3-MLT1 provides cis-regulatory elements for human placental functioning and are commonly dysregulated in human-specific preeclampsia
Source: Genome Biol. 2025 Nov 5;26:364. doi: 10.1186/s13059-025-03821-1 (PMC12587658; doi:10.1186/s13059-025-03821-1)
Supplement: Supplementary file 14 — Additional file 14: Availability of Data and Materials. [file 13059_2025_3821_MOESM14_ESM.pdf]

Availability of Data and Materials	Reference
**RNA-seq data** on human tissues.	Ebi.ac.uk/BioStudies. Illumina Body Map 2.0; E-MTAB-513
**RNA-seq data** on human tissues.	GTEx data; PMID: 25954002
**Single cell RNA-seq data** on human placenta tissue from 8th and 24th week of gestation.	GEO accession: GSE89497; PMID: 30042384
**ChIP-seq data on** binding sites of<br>• GATA2, GATA3, TFAP2A, TFAP2C transcription factors in trophoblast progenitors derived from human embryonic stem cells (H9)<br>• H3K9Ac, H3K27Ac (active) and H3K27Me3 (repressive) histone marks in differentiated syncytiotrophoblasts<br>• H3K4Me1 and H3K27Ac in human term placenta tissue.	GEO accession: GSE105081; PMID: 29078328<br>GEO accession: GSE127288; PMID: 31294776<br>GEO accession: GSE118289; PMID: 30231016
**Microarray data** on human placenta tissue (Oslo-Cohort).	PMID: 28904069
**RNA-seq** data of 10 EO-PE and 8 healthy placental samples	Anwar, R. Pande, A. Hurst, L.D. Izsvak, Z. Analysis of retroviral MLT1 enhancer-driven genes identifies over-expression of EPS8L1 as an early-stage initiator of both oxidative stress and

	poor trophoblast invasiveness in the pregnancy disorder preeclampsia. Datasets. NCBI. https://www.ncbi.nlm.nih.gov/geo/query/acc.cgi?acc=GSE263305
**RNA-seq data** on EPS8L1-OE-SGHPl-4 cells and WT-SGHPl-4 cells - generated in this study	Anwar, R. Pande, A. Hurst, L.D. Izsvak, Z. Analysis of retroviral MLT1 enhancer-driven genes identifies over-expression of EPS8L1 as an early-stage initiator of both oxidative stress and poor trophoblast invasiveness in the pregnancy disorder preeclampsia. Datasets. NCBI. https://www.ncbi.nlm.nih.gov/geo/query/acc.cgi?acc=GSE263305
**Bisulphite** sequencing	Anwar, R. Datasets. FigShare.<br>Doi: 10.6084/m9.figshare.29866184
**Enhancer prediction**	https://github.com/amitpande74/human-transposons-enhancer-predicition<br>Reference: Pande, Amit, Eve, Hallett Enhancer prediction. https://doi.org/10.5281/zenodo.16022218 (2025)
Full versions of **gels, IF and IHC images**	DOI:10.6084/m9.figshare.29738765
