# Supplementary material for: ERV3-MLT1 provides cis-regulatory elements for human placental functioning and are commonly dysregulated in human-specific preeclampsia
Source: Genome Biol. 2025 Nov 5;26:364. doi: 10.1186/s13059-025-03821-1 (PMC12587658; doi:10.1186/s13059-025-03821-1)
Supplement: Supplementary file 15 — Additional file 15: Detailed author’s contributions. [file 13059_2025_3821_MOESM15_ESM.pdf]

Contributing authors:

All the authors accepted the final version of the manuscript for submission.

Rabia Anwar = rabia.nust@gmail.com
Experimental design, performed most of the experiments, analysis, writing

Amit Pande = Amit.Pande@mdc-berlin.de
Bioinformatic analysis, ERV-derived enhancer prediction

Manvendra Singh = manvendra.singh@mpinat.mpg.de
Bioinformatic analysis

Zhi Huang = Huang, Zhi Zhi.Huang@mdc-berlin.de
Knocking out MLT1G1

Eve Hallett = ekh36@bath.ac.uk
Bioinformatic analysis, ERV-derived enhancer prediction

Yiran Xie = xieyiran1218@gmail.com
Performing the spatial EPS8L1 placental analysis, IHC of EPS8L1 and analyses. Data analysis, discussion.

Alexandra Gellhaus = Alexandra.Gellhaus@uk-essen.de
Provided the Essen PE Cohort including samples and clinical data. Contributed with data analysis and discussion

Florian Herse = florian.herse@charite.de
Handled cohorts and supervised/designed expression studies and primary cell isolation. Contributed with data analysis and discussion

Tamás Raskó = rasko_t@ukw.de
Designed and supervised for sample preparation for EPS8L1 protein interactome study. Contributed with data analysis discussion Supervised ROS experiments

Martin Gauster = martin.gauster@medunigraz.at
Provided the Graz Cohort, consisting of placental villous tissue from the first trimester. Contributed with data analysis and discussion

Olivia Nonn = olivia.nonn@charite.de
Graz Cohort design/recruitment, qPCR analysis of Graz Cohort. Contributed with data analysis

Matthias Selbach = matthias.selbach@mdc-berlin.de
Supervised/performed protein interactome studies

Stefan Verlohren = s.verlohren@uke.de
Provided the Charité PE Cohort including samples and clinical data. Contributed with data analysis discussion.

Anne Cathrine Staff = a.c.staff@medisin.uio.no
Provided the Oslo PE Cohort including samples and clinical data. Contributed with data analysis discussion, revised manuscript.

Ulrich Pecks = Pecks_U@ukw.de
Provided the Aachen PE Cohort including samples and clinical data.

Ralf Dechend = ralf.dechend@charite.de
Co-supervised the experimental part of the study, contributed with data analysis and discussion

Sandra M. Blois = s.blois@uke.de
Supervised the work on spatial EPS8L1 placental analysis. Contributed with data analysis, discussion, revision of the manuscript

Laurence D. Hurst = bssldh@bath.ac.uk
Writing, correspondence, lead communicating

Zsuzsanna Izsvák = zizsvak@mdc-berlin.de
Supervising the study, writing, correspondence
